# Oral Health Status, Oral Health Behaviours and Oral Health Care Utilisation Among Migrants Residing in Europe: A Systematic Review

**DOI:** 10.1007/s10903-020-01056-9

**Published:** 2020-07-19

**Authors:** Amandeep Pabbla, Denise Duijster, Alice Grasveld, Caroline Sekundo, Charles Agyemang, Geert van der Heijden

**Affiliations:** 1 Department of Social Dentistry, Academic Centre for Dentistry Amsterdam (ACTA), University of Amsterdam and VU University, Gustav Mahlerlaan 3004, 1081 LA Amsterdam, The Netherlands; 2 Department of Conservative Dentistry, Clinic for Oral, Dental and Maxillofacial Diseases, University Hospital Heidelberg, Heidelberg, Germany; 3 Department of Public Health, Academic Medical Centre (AMC), University of Amsterdam, Amsterdam, The Netherlands

**Keywords:** Migrants, Oral health status, Europe, Oral health behaviours, Oral health care utilisation

## Abstract

As the reported data on oral health status among the migrants in Europe is fragmented, we systematically reviewed the published literature on the oral health status, behaviours and care utilisation among migrants residing in Europe. For this, we retrieved publications from PubMed and EMBASE, supplemented by manual citation screening and grey literature search on Google scholars. Two independent reviewers screened the studies, extracted data and critically appraised the publications. A total of 69 studies included showed higher dental caries among migrant children. But some studies on adolescents and adults reported similar or even better oral health among migrants compared to the host population, while other reported the opposite. Poor oral health behaviours were generally reported among the migrants and they frequently made use of emergency service utilisation compared to the host population. We shed light on the gaps in dental literature and make some recommendations for the future.

**Electronic supplementary material:**

The online version of this article (10.1007/s10903-020-01056-9) contains supplementary material, which is available to authorized users.

## Introduction

The last few decades have seen the consolidation and expansion of free movement in the European Union (EU) regime. This has generated a migratory movement of people from both within and across the globe, many of whom are highly skilled and actively contributing to the economic and labour market of Europe. The United Nation Migration Agency (IOM) defines a migrant as any person who is moving or has moved across an international border or within a State away from his/her habitual place of residence, regardless of (1) the person’s legal status; (2) whether the movement is voluntary or involuntary; (3) what the causes for the movement are; or 4) what the length of the stay is [1]. With approximately 22.3 million migrants residing within the EU, they are now increasingly becoming a part of the European society [2]. However, the migration phenomenon itself is not without challenges. Dealing with the social, economic and emotional uprooting can negatively influence the quality of life of migrants, which can be detrimental to their health, including their oral health [3]. Foreseeably, implications of poor oral health among migrants are steadily gaining recognition as an important issue in research and policy making [4].

It has been generally observed that when migrants from low and middle income countries migrate to high income countries such as the USA, Canada, Australia and Europe, they are at higher risk of poor oral health [5–8]. For instance, a systematic review showed inadequate oral health knowledge, attitudes and practices among South Asian migrants, mainly influenced by culture, social norms and religiosity [5]. Poor oral health behaviours were reported by another study, where migrants brushed their teeth once daily and had higher frequency of sugar consumption compared to the host population [6]. Acculturation is another important facet of migration that can be detrimental or beneficial for oral health. Highly-acculturated migrant populations demonstrated better oral health outcomes and behaviours, high oral health care utilisation and improved dental knowledge, compared to the those who were poorly acculturated [7, 8]. Therefore, given its vast array of possible consequences for physical, social and economic well-being, poor oral health can become a major deterrent for the migrant population. Yet, research on migrant oral health is far from comprehensive.

Reporting of oral health status among the migrant population in Europe is sparse and fragmented. In addition, data on existing oral health among migrants and their determinants have not been systematically evaluated. As a consequence, migrants usually fail to get mentioned in oral health development goals such as the Global goals for oral health 2020 [9] or the European Global Oral Health Indicators Development Project [10]. Subsequently, any possibility of improving oral health amongst migrants becomes disparaged and ambivalent. Therefore, we aimed to systematically assess the current oral health status, oral health behaviours and oral health care utilisation among migrants residing in Europe. To the best of our knowledge this is the first systematic review to focus comprehensively on oral health status and determinants of oral health, including access of oral health care and utilisation patterns among the migrant population in Europe.

## Methods

### Eligibility Criteria

In this systematic review, we included all original studies addressing one or more of the three oral health aims, namely self-reported or clinically examined oral health status (dental caries, periodontal diseases, oral cancer, orthodontic problem) and/ or self-reported oral health behaviours (tooth brushing, fluoride use, sugar consumption, feeding practices, tobacco and alcohol consumption) and/ or oral health care utilisation (dental attendance/ barriers faced) among the migrants. The included studies were restricted to the research conducted on the migrants living in Europe. We included all the studies that referred to migrants using various terminologies such as minority groups, ethnic groups, immigrants, Black and minority ethnic groups (BME), the studies referring to only one ethnic group such as South Asians, African Caribbean’s, Chinese, Turkish, Moroccan or Eastern Europeans. We restricted this review to studies published from the year 2000 onwards to ensure that oral health status of current European migrant groups could be assessed with little possibility of generational comparisons among migrants.

Reviews, clinical case studies, qualitative studies, case reports, letters and editorials were excluded. Also, studies focusing on refugees or asylum seekers or undocumented migrants were not included as the factors governing this group are different from the regular migrant population.

### Data Sources and Search Strategy

**Fig. 1:**
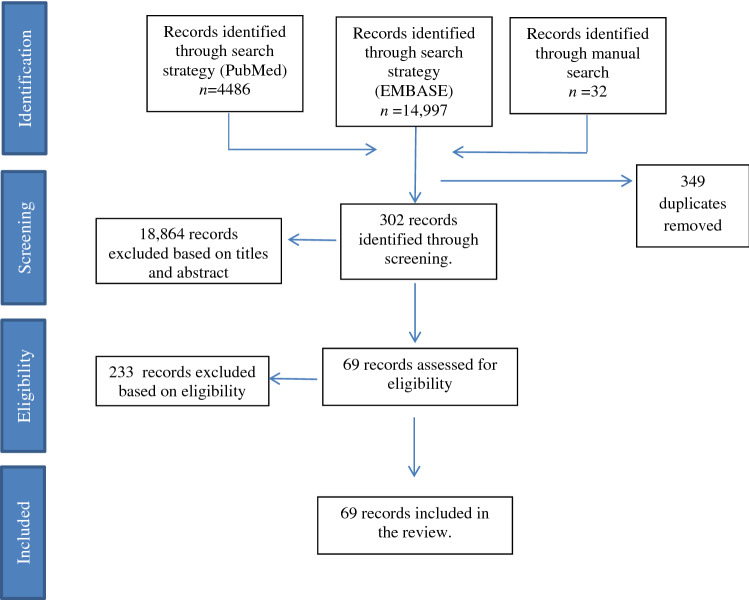
Flow chart of literature according to PRISMA flowchart: the identification, screening and inclusion of studies

We followed the PRISMA guidelines ‘Reporting Systematic Reviews and Meta-Analyses’ [11]. A comprehensive search was conducted up to October 20, 2019 using the electronic bibliographic databases PubMed and EMBASE. We established a set of relevant MeSH terms and text key words for a search in PubMed and adapted these for a similar search in EMBASE using emtree (Appendix Table S1). Selection of relevant the studies was performed by two independent reviewers who screened the titles and the abstracts to select potentially relevant records. A full text screening of relevant titles and abstracts was then performed according to the inclusion criteria. Additionally, we used manual cross-reference screening to find any potentially relevant studies that were missed with the electronic searches. Finally, we conducted a grey literature search by using the same keywords on Google Scholar and Google search engine. Search results were exported to Mendeley and duplicates were removed. A flow chart of the selection of the studies is presented in Fig. 1.

### Screening and Data Extraction

Data from the selected studies were extracted under four headings: 1) *general study characteristics*: aim, mentioned ethnicity or migration status, sampling method and sample size, age of the target population and study design, 2) *oral health outcomes*: oral health status measured using clinical indices or self-reported via questionnaires or interviews, 3) *oral health behaviours*: behaviours including tooth brushing habits, fluoride use, dietary sugar consumption, smoking and drinking reported through self-reported questionnaires or as interviews, and 4) *oral health care utilisation*: dental visits or dental attendance, type of dental treatment and barriers, if mentioned. For oral health status, behaviours and care utilisation, data on migrants were extracted and inter and/ or intra ethnic comparisons were made.

### Quality Assessment

We used the AXIS critical appraisal tool: AXIS CAT [12] to systematically assess the studies. This scale is especially designed for appraising cross sectional studies and includes 20 items that measure three domains: the quality of study design (7 components), quality of reporting (7 components) and risk of bias (6 components). The AXIS tool does not include a numerical scale for cumulative values for appraisal, instead the tool assesses the individual characteristics of a study through these components in a descriptive manner.

All three steps; data screening, data extraction and quality assessment were carried out independently by two reviewers at all given steps. Initial disagreements between the reviewers were resolved by back and forth discussion until consensus was reached. Five studies from Germany were not in English, but one co-author was consulted as native speaker to help with the data extraction and critical appraisal of these five studies.

### Syntheses of Results

Due to the heterogeneity of the outcome measures and different qualities, we were unable to perform meta-analysis. Hence, findings of these studies were evaluated in a descriptive manner.

## Results

### General Characteristics of the Studies

We included 69 studies that met the final inclusion criteria. Summary results are reported in Tables 1, 2 and 3 and detailed results per study are shown in Appendix Table S2 to S4. These studies were from the United Kingdom (n = 29), Germany (n = 10), Sweden (n = 9), Norway (n = 5), Italy (n = 5), Spain (n = 4), The Netherlands (n = 2), Denmark (n = 2), Greece (n = 2) and Austria (n = 1). In these studies, the method of recording ethnicity was self-assessed (n = 40) or by visual method (n = 2) or via official records (n = 13). In 14 studies, the method of recording ethnicity was not clear. The target population varied: 38 studies studied immigrants/ or migrants in general, 18 studies used the term BME, which included South Asians and African Caribbean’s and 12 studies included specific ethnic groups such as only South Asian or Chinese or Turkish or Moroccan population. One study from Denmark examined ethnic groups including Somalian, Albanian, Arabian and Pakistani migrants. Henceforth, for the sake of clarity, we will use the term ‘migrants’ for all target groups stated above and the term ‘host population’ for the native population or comparison group, wherever required. Age-wise distribution of the target population was children: 0–12 years (n = 35), adolescents: 12–16 years (n = 18) and adults: 16 years and above (n = 30). The sampling techniques used in these studies were random sampling (n = 27), convenience sampling (n = 25) and secondary data through medical records (n = 10). In more than half of these studies, comparisons were made with the host population (n = 38) (see Table 1 and Appendix Table S2).

**Table 1:** Summary of general characteristics of the studies

Countries n	Overall aims addressed	Ethnicity	Population characteristics*	Sampling methodology	Study design
United Kingdom (n = 29)	Clinically examined oral health status (n = 14) Dental caries (n = 11) Periodontal diseases (n = 2) Oral cancer, including oral lesions (n = 2) Orthodontic (n = 2) Cleft problems (n = 1) Self-reported oral health (n = 5) Oral health behaviours (n = 12) Tooth brushing, dietary practices (n = 4) Smoking, alcohol consumption (n = 9) Oral health knowledge (n = 2) Oral cancer awareness (n = 4) Oral health care utilisation (n = 6) Dental attendance (n = 6) Barriers (n = 2)	Method of recording ethnicity Self-assessed (n = 15) Official records (n = 8) Visual method (n = 2) Not clear (n = 4) Categorization of ethnicity Black and ethnic minority group BME: (n = 21) Immigrants/ migrants (n = 1) One particular ethnic groups such as South Asians or Turkish or Chinese (n = 7)	Children: 0–12 years (n = 11) Adolescents: 12–16 years (n = 4) Adults: 16 years and above (n = 7)	Convenience (n = 13) Random (n = 10) Secondary data (n = 4) Not clear (n = 2)	Cross sectional, comparisons with host population made (n = 11) Cross sectional, no comparisons with made (n = 5) Cross sectional, only intra ethnic comparisons made (n = 6) Cross sectional, registry based (n = 3) Cross sectional, intra ethnic comparisons and comparisons with host population made (n = 4)
Germany (n = 10)	Clinically examined oral health status (n = 6) Dental caries (n = 6) Periodontal diseases (n = 1) Orthodontic (n = 1) Use of sealants (n = 3) Oral health behaviours (n = 3) Tooth brushing, dietary practices (n = 2) Oral health knowledge (n = 1) Oral health care utilisation (n = 6) Dental attendance (n = 6) Barriers (n = 2)	Method of recording ethnicity Self-assessed (n = 3) Official records (n = 1) Not clear (n = 6) Categorization of ethnicity Immigrants (n = 9) Turkish (n = 1)	Children: 0–12 years (n = 4) Adolescents: 12–16 years (n = 4) Adults: 16 years and above (n = 5)	Convenience (n = 3) Random (n = 2) Secondary data (n = 2) Not clear (n = 2) All included (n = 1)	Cross sectional, comparisons with host population made (n = 7) Cross sectional, registry based (n = 2) Longitudinal study, comparisons with host population made (n = 1)
Sweden (n = 9)	Clinically examined oral health status (n = 9) Dental caries (n = 6) Oral cancer (n = 1) Self-reported oral health (n = 2) Oral health behaviours (n = 5) Tooth brushing, dietary practices (n = 5) Oral health knowledge (n = 2) Oral health care utilisation (n = 3) Dental attendance (n = 3) Barriers (n = 1)	Method of recording ethnicity Self-assessed (n = 5) Official records (n = 2) Not clear (n = 2) Categorisation of ethnicity Immigrants/migrants (n = 9)	Children: 0–12 years (n = 5) Adolescents: 12–16 years (n = 4)Adults: 16 years and above (n = 4)	Convenience (n = 2) Random (n = 5) Registry based (n = 2)	Cross sectional, comparisons with host population made (n = 6) Cross sectional, no comparisons with host population (n = 1) Longitudinal registry based, comparisons with host population made (n = 1) Cross sectional, registry based (n = 1)
Italy (n = 5)	Clinically examined oral health status (n = 4) Dental caries (n = 4) Oral health behaviours (n = 1) Smoking, alcohol consumption (n = 1)	Method of recording ethnicity Self-assessed (n = 4) Not clear (n = 1) Categorisation of ethnicity Immigrants/migrants (n = 4) Non-westerns/foreigners (n = 1)	Children: 0–12 years (n = 4) Adults: 16 years and above ( n = 1)	Convenience (n = 1) Random (n = 3) Not clear ( n = 1)	Cross sectional, comparisons with host population (n = 4) Cross sectional, only intra ethnic comparisons made (n = 1)
Norway (n = 5)	Clinically examined oral health status: (n = 4) Dental caries (n = 4) Oral health behaviours (n = 4) Tooth brushing, dietary practices (n = 4) Oral health knowledge (n = 3)	Method of recording ethnicity Self-assessed (n = 4) Not clear (n = 1) Categorisation of ethnicity Immigrants/migrants (n = 5)	Parents of children aged 3–5 years (n = 5)	Convenience (n = 3) Random (n = 2)	Cross sectional, comparisons with host population (n = 2) Cross sectional, follow up (n = 3)
Spain (n = 4)	Clinically examined oral health status (n = 4) Dental caries (n = 3) Periodontal diseases (n = 1) Self-reported oral health (n = 1) Oral health behaviours (n = 1) Tooth brushing, dietary practices (n = 1) Oral health care utilisation (n = 1) Dental attendance (n = 1)	Method of recording ethnicity Self-assessed (n = 4) Categorisation of ethnicity Immigrants/migrants (n = 4)	Children: 0–12 years (n = 2) Adolescents: 12–16 years (n = 3) Adults: 16 years and above ( n = 1)	Convenience (n = 1) Random (n = 2) Registry based (n = 1)	Cross sectional, comparisons with host population (n = 3) Cross sectional, registry based (n = 1)
The Netherlands (n = 2)	Clinically examined oral health status (n = 1) Dental caries (n = 1) Oral health behaviours (n = 1) Tooth brushing, dietary practices (n = 1) Oral health knowledge (n = 1)	Method of recording ethnicity Self-assessed (n = 2) Categorisation of ethnicity Non-Dutch (n = 1) Moroccan/Turkish (n = 1)	Children: 0–12 years (n = 2)	Convenience (n = 1) Total population taken (n = 1)	Cross sectional, comparisons with host population (n = 1) Cross sectional: case–control study, comparisons with host population made (n = 1)
Denmark (n = 2)	Clinically examined oral health status (n = 2) Dental caries (n = 2) Oral health behaviours (n = 1) Tooth brushing, dietary practices (n = 1) Oral health knowledge (n = 1)	Method of recording ethnicity Self-assessed (n = 1) Official records (n = 1) Categorisation of ethnicity Immigrants/migrants (n = 1)	Children: 0–12 years (n = 2) Adolescents: 12–16 years (n = 2)	Convenience (n = 1) Registry based (n = 1)	Cross sectional, comparisons with host population (n = 1) Cross sectional, registry based (n = 1)
Greece (n = 2)	Clinically examined oral health status (n = 2) Dental caries (n = 2)	Method of recording ethnicity Official records (n = 2) Categorisation of ethnicity Immigrants/migrants (n = 1) Non-Greek (n = 1)	Children: 0–12 years (n = 2)	Random (n = 2)	Cross sectional, comparisons with host population (n = 2)
Austria (n = 1)	Clinically examined oral health status (n = 1) Dental caries (n = 1)	Method of recording ethnicity Self-assessed (n = 1) Categorisation of ethnicity Immigrants/migrants (n = 1)	Children: 0–12 years (n = 1)	Random (n = 1)	Cross sectional, comparisons with host population (n = 1)

*n *total number of studies

*Overlap due to multiple methods used in these studies

### Critical Appraisal

Detailed results on the critical appraisal per study are in Appendix Table S5 to S7 and a summary of the appraisal is presented in Appendix Table S8. For the first domain, ‘quality of study design’, the sample size and sample frame were justified by approximately half the studies (60.8% and 56.5% respectively). Overall, only three studies fulfilled all seven components of this domain [13–15]. Regarding the second domain, ‘quality of reporting’, 20 studies fulfilled all the seven components mentioned under this domain. Lastly, the third domain reported the ‘risk of bias’. The survey response rate of ≥ 60% was taken as the cut off as this addresses the non-response bias, based on representativeness of the sample [16]. In our review, we found that only 34.7% of the studies raised no concerns (response rate > 60%). Only one study fulfilled all the six components in this domain. Overall critical appraisal of these 69 studies revealed that only one study fulfilled all the components in all three domains addressing the criteria set by this appraisal tool [13].

### Oral Health Status

53 studies (n = 53) reviewed oral health status among the migrant population in Europe (Table 2and Appendix Table S3). These studies assessed oral health through self-reported questionnaires or interviews (n = 8) and/ or through clinical assessment (n = 41) or through secondary data from hospital records (n = 6). Irrespective of the source of data collection, these studies focused on various oral diseases including dental caries status (n = 40), periodontal diseases (n = 5), oral cancer including oral lesions (n = 3), orthodontic problems (n = 3), gingival bleeding (n = 2) and cleft issues (n = 1). Most frequently assessed oral health status was dental caries experience, often expressed using the decayed, missing and filled teeth (DMFT) or surfaces (DMFS) index, which was significantly higher among migrant children compared to host population (n = 29). On the other hand, dental caries experience among adolescent migrants (n = 11) varied. Studies from the United Kingdom, Sweden and Denmark showed lower dental caries among migrant adolescents compared to the host population. However, the studies from Germany and Spain reported higher dental caries among migrant adolescents compared to the host population. A total of seven studies reported the dental caries status among the adult population. Similar discrepancies in results were noted among adult population as well. Clinically examined dental caries experience was reported to be better among adult migrants compared to the host population in the studies from the United Kingdom, whereas studies from Germany and Sweden reported dental caries to be higher among migrants compared to the host population. Studies from the United Kingdom showed better observed self-reported oral health among migrants compared to the host population. But the studies from Sweden and Spain reported otherwise.

**Table 2:** Summary of oral health status

Countries	Self-reported oral health (questionnaire/ interviews)	Objective oral health (clinical examination)			
		Dental caries (DC)	Periodontal diseases	Oral cancer	Others
United Kingdom (n = 20)	4 Studies Pakistani reported toothache and Chinese reported sensitivity as most frequent problem Satisfaction with teeth and gums was highest among Africans compared to Asians Overall, impact of oral pain was highest among Chinese and lowest among Black Africans 1 Study on oral health related quality of life (OHRQoL) Migrants had similar or even better OHRQoL compared to the HP	6 Studies (children), including 1 study through records Dental caries experience among 5 year old migrant children was statistically higher compared to the host population Eastern European children had significantly higher dental caries compared to other ethnic groups Among BME group, Black African children had lesser dental caries experience compared to all other groups Intra-ethnically, Pakistani and Muslim 5 year old children had significantly worst dental health compared to other ethnic groups and non-Muslim children 2 Studies (adolescents) In the age group of 12 years and above, migrant teenagers report similar or even better oral health compared to the host population South Asian adolescents showed significantly lesser dental caries and tooth erosion compared to the host population 4 Studies (adults), including 1 study through records Overall, migrants exhibited lower dental caries experience and lower edentulousness compared to the host population Intra-ethnically, prevalence of untreated decay was highest among the South Asians compared to Black Caribbean’s	1 Study (children) Higher gingival bleeding and plaque accumulation was reported among Bangladeshi children compared to the host population 1 Study (adults) Asians had higher pocket depth compared to White British Loss of attachment was higher among Eastern European, Black Africans and Bangladeshi group	1 Study (oral lesions) Leukoplakia was significantly associated with paan chewing with or without tobacco among migrants 1 Study (oral cancer) Highest rates of *oral cancer* were seen among Bangladeshi females compared to other ethnic groups * Oropharyngeal cancer* was lowest in all ethnic groups compared to the host population * Nasopharyngeal cancer* was highest among Chinese	2 Studies (adolescents) Orthodontics Black children perceived their need for orthodontics differently to other populations 1 Study (children) Cleft Cleft speech characteristics were not related to ethnicity in children
Germany (n = 6)	Not studied	4 Studies (children) Dental caries experience was statistically higher among migrant children was compared to the host population. Fissure sealants were lower among migrant children compared to the German children 3 Studies (adolescents) Higher dental caries experience was reported among migrants compared to the host population Caries free teeth increased statistically among migrant adolescents compared to the host population 2 Studies (adults) Dental caries experience among adult migrants was similar and even lower compared to the host population. Overall, migrants had lower DMFT compared to the host population	Not studied	Not studied	1 Study (adolescents) Orthodontic needs Higher needs were seen among migrant adolescents compared to the host population 1 study (adults) plaque accumulation and bleeding gums Higher accumulation and bleeding of gums reported among migrant adults compared to the host population
Sweden (n = 9)	2 Studies Odds of chewing problems and denture wearing were higher among migrants compared to the host population Overall, signs of reported poor oral health were seen among migrants compared to the host population	3 Studies (children) Higher dental caries among was reported migrant children compared to the host population 1 Study (adolescents) Dental caries became lower and almost equal between migrants and the host population in adolescents Newly arrived migrants had more initial caries that is at enamel level Migrant adolescents arriving after the age of 7 years or more had 2–3 times higher dental caries compared to the host population 1 Study through records (adolescents) Approximal caries increment showed increase among Eastern European and Asian migrants	Not studied	1 Study (adults) Higher risk of nasopharyngeal and hypopharyngeal cancer was observed among migrants, especially South Asians (Chinese) and north Africans	Not studied
Italy (n = 4)	Not studied	4 Studies (children) Early childhood caries and levels of untreated caries was significantly higher among the migrant children (3 times more probability) compared to the host children	Not studied	Not studied	Not studied
Norway (n = 4)	Not studied	5 Studies (children) Higher dental caries was seen among 3 and 5 year old migrant children compared to the host population	Not studied	Not studied	Not studied
Spain (n = 4)	1 Study Self-reported dental caries and tooth loss was among migrants was higher compared to the host population Gingival bleeding was higher among Spanish women compared to migrant women	2 Studies (children) Higher dental caries experience was observed among 12 year old migrant children compared to the host population 3 Studies (adolescents) Dental caries experience among 15 year old migrant adolescents was higher compared to the host population	Not studied	Not studied	Not studied
The Netherlands (n = 1)	Not studied	1 Study (children) Higher dental caries experience was seen among migrant children compared to the host population	Not studied	Not studied	Not studied
Denmark (n = 2)	Not studied	2 Studies (children) Dental caries among Danish children aged 3, 5 and 7 was statistically lower compared to the migrant children Only Somalin children showed lowest dental caries experience compared to all other groups 2 Studies (adolescents) Dental caries prevalence was similar among all groups Only Albanian 15 year old had statistically higher dental caries experience compared to Danish teenagers	Not studied	Not studied	Not studied
Greece (n = 2)	Not studied	2 Studies (children) Comparatively higher dental caries experience was observed among migrant children compared to the host population	Not studied	Not studied	Not studied
Austria (n = 1)	Not studied	1 Study (children) Higher dental caries experience was observed among migrant children compared to the host population	Not studied	Not studied	Not studied

*n * total number of studies

Periodontal health status, including gum bleeding and plaque accumulation (n = 5) among adult migrants was reported to be poor, with the studies from the United Kingdom, Germany and Sweden reporting poor periodontal health among migrants compared to the host population. In children and adolescents, gingival bleeding and plaque accumulation was seen to be higher among migrants, especially in Bangladeshi children. Similarly, higher pocket depths were also observed among Bangladeshi adolescents compared to the host population. Only one study from Spain reported higher bleeding gums among Spanish women compared to the migrant population [14]. Oral cancers including oral lesions were reported by three studies showing higher rates of oral lesions (leucoplakia) among Bangladeshi migrants and overall higher oral cancer rates among the South Asian community. Among other oral diseases such as orthodontic problems, migrant children had lower rates of completed orthodontic treatments compared to the host population.

### Oral Health Behaviours and Attitudes

28 studies (n = 28) reviewed oral health behaviours (Table 3 and Appendix Table S4). These studies focused on oral hygiene practices such as tooth brushing and sugar consumption (n = 18), which were generally poor among migrants compared to the host population. Most migrant parents depicted low supervision towards maintaining the oral health of their child compared to the host population, such as the brushing teeth of their children only once daily and providing more sweet snacks to their children. Adult migrants added more sugar to their hot drinks although their frequency to consume sweets and cakes was lesser compared to the host population. Oral health knowledge and beliefs (n = 14) were also generally poor among the migrants compared to the host population. Especially most South Asian Muslim migrant parents in Norway believed that oral hygiene did not influence dental caries and deciduous teeth were not important [6]. Overall, important reasons stated for poor oral hygiene and attitude towards oral health were language insufficiency, lesser confidence in their ability to assist their child in tooth brushing, over indulgence (excessive intake of sugar in food and beverages), other priorities than oral health and different diet patterns of migrants.

**Table 3:** Summary of oral health behaviours and oral health care utilisation

Countries	Oral hygiene practices (tooth brushing and sugar consumption)	Habits- tobacco and alcohol consumption	Oral health knowledge and beliefs	Oral health care utilisation patterns
United Kingdom (n = 12)	4 Studies Host population brushed their teeth twice daily compared to the migrants who only brushed once daily Migrants added sugar to their hot beverages more frequently whereas Black Africans consumed fizzy drinks more often and the host population showed higher consumption of sweets and cakes Overall, migrants consumed more sugar compared to the host population	9 Studies Most papers were intra-ethnic in comparing habits High consumption of alcohol was seen among Black Africans in BME group Amongst Indians, largest consumer of alcohol were Hindus and Sikhs Highest consumers of chewing tobacco were South Asians Muslims Smoking was similar in all ethnic groups although Black African started at a younger age	2 Studies Poorer knowledge and attitude towards oral hygiene practices were seen among migrants compared to the host population 4 Studies on oral cancer awareness Most papers were with intra-ethnic comparisons Low oral cancer awareness was seen among Indians compared to other groups Negative attitude towards chewing tobacco was observed among Bangladeshi adolescents Main reasons stated by migrants were that tobacco was stress reliever and had good taste	6 Studies Lower dental attendance among migrant was seen compared to the host population. groups Barriers stated were language insufficiency and lower information on available oral health options Similar or better dental attendance among women and Asians was seen compared to men and the host population Reason for dental visits were mostly emergency (tooth ache and denture repair) among migrants compared to the host population (preventive and routine dental check-ups) Private dental visit were more frequent among the host population compared to the migrants Recommendations to improve accessibility, affordability and acceptability were made by the migrants
Germany (n = 3)	2 Studies Lower tooth brushing (once daily) was seen among the migrants compared to the host population (twice brushing) Also, migrant parents started brushing the teeth of their children late, had lesser time of supervision compared to the host population	Not studied	1 Study Negative attitude towards dentist was expressed by Turkish parents (dentist made them feel guilty about the poor oral hygiene of their child) German parents had better awareness of the information on cost and available dental services compared to the migrants	6 Studies Lower dental visits per year were reported by the among migrants compared to the host population Mostly older migrants made use of dental services compared to the younger migrants Barriers were cost concerns, language, anxiety and problems in making appointments Reasons for dental visits were emergency care among migrants compared to preventive care in the host population
Sweden (n = 5)	5 Studies Host population brushed their teeth twice daily compared to the migrants who only brushed once daily Low supervised tooth brushing was seen among migrant parents compared to the host population Higher sugary snack intake among migrant children was seen compared to the host population	Not studied	2 Studies Migrant parents attended dental awareness meetings less frequently compared to the host population	5 Studies Dental visits were less frequent among migrants compared to the host population Barriers reported were higher socio economic stress and unemployment among migrants
Italy (n = 1)	Not studied	1 Study on Asian adults Intra ethnically, higher BQC (betel quid chewing) was seen among Pakistani and Indian males compared to other migrant groups Most Asians believed that, BQC relieves stress	Not studied	Not studied
Norway (n = 4)	4 Studies Migrant parents were more indulgent and provided sugary snacks for their children compared to the host population Also, bed time snacking was more frequent among migrant children compared to the host population	Not studied	3 Studies Migrants believed that dental caries happened by luck, oral hygiene did not influence dental caries and deciduous teeth were not important Intra ethnically, Muslim parents were less bothered about the oral health of children compared to non-Muslim patents	Not studied
Spain (n = 1)	1 Study Lower tooth brushing habits were seen among migrant children compared to the host population Sugary foods consumption among 15 year old migrant adolescents was higher compared to the host population	Not studied	Not studied	1 Study Dental visits were less frequent among migrants compared to the host population
The Netherlands (n = 1)	1 Study Consumption of sugary foods between meals was more frequently reported among migrant children compared to the host population	Not studied	1 Study Dental caries was associated with higher dental efficacy and more internal LoC and higher SES as seen among Dutch parents compared to the migrants	Not studied
Denmark (n = 1)	1 Study Migrant children started brushing their teeth at a much higher age compared to the host population Migrant parents provided kisser support in brushing the teeth of their children compared to the host population Also, higher consumption of sugary foods was seen among migrant children compared to the host population	Not studied	1 Study Migrants believed that taking care of the teeth of their children was the responsibility of the kindergartens	Not studied

*n *total number of studies

Tobacco and alcohol consumption (n = 10) were mainly studied in the United Kingdom, with mostly intra-ethnic comparisons made. Among the BME group, Black African population was a heavy consumer of alcohol compared to other migrant groups. No differences in tobacco smoking rates were reported between migrant groups, although the Black Africans started the habit at a younger age compared to other migrant groups. Consumption of smokeless forms of tobacco such as chewing tobacco was comparatively higher among South Asian migrants, especially the Bangladeshi migrants and Muslim South Asians compared to other South Asian groups and non-Muslim South Asians. Awareness towards risks of oral cancer was lower among migrants. Most migrants associated chewing tobacco as being a stress buster and having good taste.

### Oral Health Care Utilisation

16 studies (n = 16) reported on the oral health care utilisation among migrants (Table 3 and Appendix Table S4). These studies evaluated dental attendance and barriers encountered, reasons stated for dental visits and type of dental service used. Overall, utilisation of oral health services was seen with respect to dental visits in the last 12 to 24 months. Intra-ethnically, South Asian migrants were more likely to have visited the dentist in the last two years than Black African migrants. The host population was more accustomed to preventive treatments and regular dental visits whereas the migrants had more emergency treatment approach with tooth ache and denture repair being the most frequent reasons for dental visits. Apart from this, the host population made use of private dentists more often than the migrants. Studies from Germany and Sweden showed that the barriers reported for dental healthcare utilisation patterns by migrants were dental cost and financial burden associated with dental visits. Other barriers reported were lower education level of the migrants, unemployment and language difficulties. In addition dental inaccessibility and unawareness regarding the existing health care delivery systems, affordability and different belief systems than the host population were also reported as barriers.

## Discussion

The key findings of our review reveal that throughout these studies, dental caries prevalence was reported to be higher among migrant children compared to the host child population. However, we observed discrepancies among adolescents and adults dental caries experience across different countries. Oral cancer was reported to be higher among South Asian communities compared to host population and other migrant groups. Oral health behaviours among migrants were generally poor compared to the host population, with intra-ethnic comparisons showing that Muslim South Asian migrants have poorer oral health behaviours compared to the non-Muslim South Asian migrants. Habits such as tobacco and alcohol consumption were mainly reported intra-ethnically with the Black African population being heavier consumer of alcohol compared to other migrant groups. Chewing tobacco was predominantly reported to be higher among Bangladeshi and Muslim South Asian communities. With regards to the utilisation of oral health services, use of emergency services was higher among migrants compared to the host population which made more use of preventive services.

Oral health status was mainly measured as dental caries experience (DMFT/DMFS) clinically and was consistently reported to be higher among migrant children. Reported determinants that may explain these ethnic inequalities in childhood dental caries varied from country to country. Higher dental caries prevalence was related to religion (United Kingdom) [17], maternal education level (Germany, The Netherlands) [15, 18] and parental attitudes and over indulgence (Norway) [6, 19]., However, the status of oral health among adolescents and adults was inconclusive. It has been established that adolescents reorient their oral behaviours (self-help in tooth brushing) and have more freedom in purchasing decisions, such as buying snacks and beverages. However, certain underlying factors such as age at migration, parental background, dietary changes and cultural norms seems to play an influencing role in determining the oral health status of adolescents [20]. As a result, this shift from age 5 to 15 among migrants merits further research as rationales supporting these findings are ambiguous [20, 21]. Among adults, the higher dental caries prevalence among Eastern European migrants sheds light on the variation within different ethnic background. Other studies reflect that migrants adapt to unhealthy diet patterns depending upon their stay in the host country [22, 23]. This is indicative that oral health of the migrants is associated with changes in lifestyle and socioeconomic position of the receiving country as well as the sending country.

Oral hygiene practices were relatively poor among migrants compared to the host population. Most commonly stated reasons were the inability of the migrant parents to supervise their children in tooth brushing, over indulgence in providing sugary foods to their children, lower attendance in meetings that provide dental information and poor knowledge and belief in oral health care of the host country [17, 18]. It is noteworthy that some studies have reported similar or even better oral health status among migrants compared to the host population, despite their poor oral hygiene practices. Hence, these findings can serve as a pointer for incorporating scientifically robust behavioural models and concepts while studying the oral health of the migrants. Health belief models such as locus of control [24] or sense of coherence [25] can aid in establishing concrete observations on oral health behaviours of migrants. Smoking and alcohol consumption among migrants varied intra ethnically indicating younger age of starting habits, ethnic composition, socioeconomic status, cultural and religious beliefs being associated with the formation of these habits [26, 27].This calls for preventive strategies including oral health promotion and education that need to be tackled sensitively from cultural aspect of a community.

The findings regarding the utilisation of oral health services are rather varied and hence the interpretation needs to be done cautiously. Dental visits did not always reveal the place of visit (home or host country), which is an important determinant to be considered when focusing on migrants [28]. Apart from ethnicity, gender predilection was also observed. Being a woman with a migrant background indicated more frequent dental visits and better awareness towards oral health compared to migrant men [13, 29]. In addition, lack of proficiency in the host language was an important determinant observed [28, 29]. Most studies reported migrants to be more inclined towards emergency care and visited private dentists less frequently compared to host population [28]. Although financial and language barriers seem obvious factors, they may stem from a broader and deeper source, such as the education level and acculturation. To study such intricate associations, long term follow up designs with qualitative approach are required. Another interesting finding was the lower utilisation of oral health services by migrant children [28] as most European countries provide free dental treatments for all children uptill the age of 19 years. This implies that oral health care utilisation patterns among the migrants need to be researched from three aspects. From the patient level, important barriers that need to be investigated are cultural specific perceptions and beliefs about oral health and illness, oral health literacy levels (including the ability to find, read and understand oral health information) and lower awareness of existing oral health care system in the host country (including type of insurance available, reimbursement forms). Similarly from the provider as well as the system level, a culturally sensitive approach is required, not only when dealing with migrants’ oral health issues but also when planning preventive programs or providing dental treatments to them.

This review highlights the oral health status of the migrants mainly through cross-sectional research designs. However, to establish temporal associations, we need to build on the available observations though longitudinal and/ or qualitative studies. Research in assessing general health among migrants, for instance has made use of three approaches: (1) by comparing the migrants with the host populations in the countries of settlement—*ethnic inequality*; (2) by comparing similar migrant populations living in different countries—*the role of contexts*, and (3) by comparing migrants with the population in their home countries—*the role of migration* [30]. We still need to incorporate these approaches to study oral health of migrants and possibly fill the existing gaps in dental literature. In addition these studies have established repeatedly that migrants are not a homogenous group; rather heterogeneity is an essential characteristic of this target population. This observation of ‘labelling’ migrants under an umbrella term, leading to debatable results has been often pointed out in literature [31]. We also observed that the influence of acculturation and migration status in oral health utilization needs to be explored in the light of relevant health concepts such as Berry’s acculturation model [32] or Andersen’s health utilization model for vulnerable population [33]. Also, studies on the underlying psychosocial determinants, such as locus of control [24], sense of coherence [25], social support structures [34] will help in understanding oral health and oral health behaviours of the migrants. This will pave the way for robust oral health promotion and preventive interventions focusing on improving the oral health status of the migrants.

Our systematic review also has certain limitations. We included the studies that addressed the oral health of the migrants in Europe, although it was difficult to assess their legal status. Hence, only the studies with direct mention of refugees or asylums or undocumented migrants were excluded. We purposely included the studies published from the year 2000 onwards. This was done to avoid confusions of mixing generations and incorporating ‘descendants’ into our review. This would have led to mixing in migrant composition which would have deviated our aim of observing the existing oral health status of migrants. Also, we came across some qualitative studies that explored the use of health care services, but these studies were excluded. This was done because it would have become difficult to draw comparisons from these studies as most of the included studies were quantitative, cross sectional in design. Lastly, for the critical appraisal for this systematic review, we made use of the AXIS tool which is designed for cross sectional studies. As there is no numerical scale provided to assess the quality of papers, this leads to subjectivity. However, this tool does allow the user to give the overall assessment of the quality of paper based on evaluating all aspects of the tool.

## Conclusion

This is the first systematic review showing poor oral health among migrant children, but inconsistent results in adolescent and adult migrant population across various countries. Oral health behaviours were consistently poor among migrants but most of these studies focused on intra-ethnic comparisons. Utilisation of oral health care was also consistently lower among the migrants but several studies made intra-ethnic comparisons and overall very few studies assessed utilisation patterns. These findings point towards the available data on oral health of the migrants in Europe, which is mostly based on cross-sectional research designs. However, to establish temporal associations, we need to build on the available observations though longitudinal and/ or qualitative studies. In addition, this review also shows the influences of existing infrastructure of each country and their respective social set on the oral health of migrants. These findings serve as a platform for future research focusing on migrant oral health in order to assist policy makers to make targeted oral health programs and policies with culturally sensitive approach crucial for improving the oral health of migrants.

## Electronic supplementary material

Below is the link to the electronic supplementary material.

Supplementary file1 (DOCX 13 kb)

Supplementary file2 (DOCX 84 kb)

Supplementary file3 (DOCX 78 kb)

Supplementary file4 (DOCX 61 kb)

Supplementary file5 (DOCX 48 kb)
