# Supplementary material for: Oral Health Status, Oral Health Behaviours and Oral Health Care Utilisation Among Migrants Residing in Europe: A Systematic Review
Source: J Immigr Minor Health. 2020 Jul 19;23(2):373–88. doi: 10.1007/s10903-020-01056-9 (PMC7914188; doi:10.1007/s10903-020-01056-9)
Supplement: Supplementary file 1 — Supplementary file1 (DOCX 13 kb) [file 10903_2020_1056_MOESM1_ESM.docx]

**Appendix Table 1: Search Strategy**

**PUBMED**

*(“oral health” [Mesh] OR “dental caries”[Mesh] OR “caries”[all fields] OR “dental decay”[all fields] OR “periodontal disease”[Mesh] OR “periodontitis”[all fields] OR “tooth mobility”[all fields] OR “bleeding gums”[all fields] OR “gingivitis”[all fields] OR “halitosis”[Mesh] OR “bad breadth”[all fields] OR “toothache”[Mesh] OR “tooth pain”[all fields] OR “tooth erosion”[Mesh] OR “tooth wear”[all fields] OR “tooth diseases”[all fields] OR “oral hygiene”[Mesh] OR “dental prophylaxis”[all fields] OR “mouth wash”[Mesh] OR “dentifrices”[all fields] OR “oral rises”[all fields] OR “oral care”[all fields] OR “preventive dentistry”[Mesh] OR “cariogenic diet”[Mesh] OR “dietary sugars”[all fields] OR “chocolates”[all fields] OR “candy”[all fields] OR “carbonated drinks”[Mesh] OR “soda pops”[all fields] OR “soft drinks”[all fields] OR “energy drinks”[Mesh] OR “smoking”[Mesh] OR “pipe smoking”[all fields] OR “cigarette smoking”[all fields] OR “tobacco smoking”[all fields] OR “tobacco chewing”[Mesh] OR “tobacco consumption”[all fields] OR “smokeless tobacco”[Mesh] OR “oral tobacco”[all fields] OR “gutka”[all fields] OR “alcohol drinking”[Mesh] OR “alcohol consumption”[all fields] OR “binge drinking”[all fields] OR “dental clinics”[Mesh] OR “dentist”[Mesh] OR “dental assistant”[all fields] OR “dental receptionist”[all fields] OR “dental team”[all fields] OR “utilization”[all fields] OR “access”[all fields] OR “barriers”[all fields] OR “dental care”[Mesh]) AND (“migrants”[Mesh] OR “migrant workers”[all fields] OR “ethnic groups”[Mesh] OR “ethnicity”[all fields] OR “immigrants”[all fields] OR “minority”[all fields] OR “africans”[all fields] OR “asians”[all fields] OR “migrant children”[all fields] OR “migrant adolescents”[all fields] OR “migrant adults”[all fields] OR “migrants and transients”[Mesh]) AND(“Europe”[Mesh] OR “European union”[all fields] OR “European countries”[all fields] OR “united kingdom”[Mesh] OR “england”[all fields] OR “london”[all fields] OR “sewden”[Mesh] OR “scandanavian”[all fields] OR “nordic”[all fields] OR “norway”[Mesh] OR “finland”[all fields] OR “Denmark”[all fields] OR “netherlands”[Mesh] OR “belgium”[Mesh] OR “italy”[Mesh] OR “germany”[Mesh] OR “france”[Mesh] OR “austria”[Mesh] OR “greece”[Mesh] OR “spain”[Mesh])*

*Total= 4486 (10 March 2019)*

**EMBASE**

| #1 | 'dental caries'/exp OR 'dental caries' OR 'tooth disease'/exp OR 'tooth disease' OR 'periodontal disease'/exp OR 'periodontal disease' OR 'mouth hygiene'/exp OR 'mouth hygiene' OR 'mouth disease'/exp OR 'mouth disease' OR 'toothpaste'/exp OR 'toothpaste' OR 'carbonated beverage'/exp OR 'carbonated beverage' OR 'smoking and smoking related phenomena'/exp OR 'smoking and smoking related phenomena' OR 'tobacco use'/exp OR 'tobacco use' | 998,221 |
| --- | --- | --- |
| #2 | 'migrant' OR 'immigrant' OR 'ethnic group' OR 'ethnicity' OR 'minority group' OR 'black person' OR 'asian' | 446,846 |
| #3 | 'europe' OR 'european union' OR 'england' OR 'united kingdom' OR 'sweden' OR 'norway' OR 'austria' OR 'italy' OR 'greece' OR 'netherlands' OR 'germany' OR 'spain' OR 'denmark' | 16,264,076 |
| #4 | #1 AND #2 AND #3 | 14,997 |

*Total= 14,997 (16 April 2019)*
