# Supplementary material for: Oral Health Status, Oral Health Behaviours and Oral Health Care Utilisation Among Migrants Residing in Europe: A Systematic Review
Source: J Immigr Minor Health. 2020 Jul 19;23(2):373–88. doi: 10.1007/s10903-020-01056-9 (PMC7914188; doi:10.1007/s10903-020-01056-9)
Supplement: Supplementary file 2 — Supplementary file2 (DOCX 84 kb) [file 10903_2020_1056_MOESM2_ESM.docx]

**Appendix Table 2: Details of general characteristics of the studies**

| **Country** | **Year of Publication, Authors and Title of the article** | **Aim**  **(As reported)** | **Definition of ethnicity/ Migration status given- details if mentioned** | **Sampling method** | **Socio-economic Status (SES) of the migrants** | **Study Population**  **In age, sample size and migrant: host ratio, if given** | | **Design of the Study** |
| --- | --- | --- | --- | --- | --- | --- | --- | --- |
| **United Kingdom 1** | (2000) Robinson PG, et al [1]  *Dental caries and treatment experience of adults from minority ethnic communities living in the South Thames Region, UK* | To describe the dental caries and treatment experience of groups of people from minority ethnic communities living in the South Thames Region of England and to identify possible determinants of their oral health. | Self-assessed ethnicity  Individuals classified their own ethnicity from the list of Office of Censuses and Surveys categories.  Black Caribbean-141  Black African-134  Pakistani-123  Indian-190  Bangladeshi-78  Chinese/ Vietnamese-143  Others- 119 | Convenience sampling  Snowball sampling method was used.  Lists of ethnic minority community, groups were obtained from health authorities, health promotion units and councils for racial equality within the South Thames region. | Recorded as education level and employment. | Age | 16 years and above | Cross- sectional study  Inter-ethnic comparisons made  Comparisons with host population made from 1988 survey records |
|  |  |  |  |  |  | n  Sample size | 928 adults |  |
|  |  |  |  |  |  | Migrant/ Host  n (%) | Not clearly mentioned |  |
| **United Kingdom 2** | (2000) Newton JT, et al. [2]  *Self-assessed oral health status of ethnic minority residents of South London* | To determine the self-assessed oral health status of individuals from minority ethnic communities living in the South East of England.  *This study uses the same dataset of Robinson et al., 2000* | *This study used the same method as* Robinson et al., 2000.  Self-assessed ethnicity  Black Caribbean-141  Black African-142  Pakistani-143  Indian-224  Bangladeshi-101  Chinese/ Vietnamese-161  Others- 145 | *This study used the same method as* Robinson et al., 2000.  Convenience sampling.  Snowball sampling method was used. | *This study used the same method as* Robinson et al., 2000 | Age | 16 years and above | Cross- sectional study.  Inter-ethnic comparisons made.  No comparisons with host population made |
|  |  |  |  |  |  | n  Sample size | 1057 adults |  |
|  |  |  |  |  |  | Migrant/ Host  n (%) | Not applicable |  |
| **United Kingdom 3** | (2000) Gray M, et al [3]  *The oral health of South Asian five-year-old children in deprived areas of Dudley compared with White children of equal deprivation and fluoridation status.* | To investigate the oral health of five-year-old children from Indian, Pakistani or Bangladeshi ethnic groups (referred to as 'South Asian') living in deprived but fluoridated parts of Dudley and compare this with White children of equal deprivation and fluoridation status. | Visual method.  Ethnic origin of the children was assessed visually by the examiner using the methodology of Tod and Dodd (1985).  Indian, Pakistani and Bangladeshi (South Asian) children.  White-464  South Asian-79 | Convenience sampling  All children aged 5 years attending schools in the fluoridated part of Dudley health district were included in the initial sample. | ACORN index was used-  From here, ACORN classification code F children were selected. | Age | 5 year | Cross- sectional study.  Comparisons with host population made |
|  |  |  |  |  |  | n  Sample size | 543 children |  |
|  |  |  |  |  |  | Migrant/ Host  n (%) | Migrants-79 (14.6 %)  Host- 464  (85.4 %) |  |
| **United Kingdom 4** | (2001) Pau AKH, et al [4]  *Self-reported oral health status and oral-health related behaviours of a sample of Chinese elders in Inner London, UK: A pilot investigation* | To describe the self-reported oral health status and oral-health related behaviours of a sample of 54 Chinese elders aged 54 years and over, resident in Inner London. | Chinese elders  No information was provided on how Chinese ethnicity was defined. | Convenience sampling  The respondents were recruited from the membership of three luncheon clubs held in Chinese community centres. | Years of education was recorded. | Age | 54-87 years | Cross- sectional study.  No comparisons with host population made. |
|  |  |  |  |  |  | n  Sample size | 54 adults |  |
|  |  |  |  |  |  | Migrant/ Host n (%) | Not applicable |  |
| **United Kingdom 5** | (2001) Ahmed B, et al [5]  *Agreement between*  *normative and perceived orthodontic need amongst deprived multiethnic school children in London* | To use the aesthetic component (AC) of the IOTN to measure agreement between normative and perceived orthodontic need amongst school children from three different ethnic groups resident in London, UK. | Through records  The Registrar General classification was used to record ethnic background.  Whites-100  Blacks- 117  South Asians- 106  Others- 55 | Sampling technique not mentioned.  Children attending four state schools in North London were selected in order to ensure a wide cross-section of socio-economic and ethnic backgrounds. | Deprivation  scores, indicating socio-economic deprivation relative  to England | Age | 11-14 year | Cross- sectional study.  Comparisons with host population made. |
|  |  |  |  |  |  | n  Sample size | 378 children |  |
|  |  |  |  |  |  | Migrant/ Host  n (%) | Migrants-278  (73.6 %)  Host- 100  (26.4 %) |  |
| **United Kingdom 6** | (2003) Newton JT, et al [6]  *The self-assessed oral health status of individuals from White, Indian, Chinese and Black Caribbean communities in South-east England* | 1. To determine the level of self-assessed oral symptoms and the impact of such symptoms among individuals from four ethnic groups resident in South-east England. 2. To assess the relationship between self-assessed oral health status, age, gender, employment status, educational level and ethnicity. | Self-assessed ethnicity  Whites-100  Indians-100  Chinese-101  Black Caribbean- 65 | Network sampling | Employed and the level of education was assessed. | Age | 16 years and above | Cross- sectional study.  Comparisons with host population made. |
|  |  |  |  |  |  | n  Sample size | 366 adults |  |
|  |  |  |  |  |  | Migrant/ Host  n (%) | Migrants-266  (72.6 %)  Host- 100  (27.4 %) |  |
| **United Kingdom 7** | (2005) Dugmore CR, et al [7]  *The effect of socio-economic status and ethnicity on the*  *comparative oral health of Asian and White Caucasian 12-*  *year-old children* | 1. To investigate differences in oral health between 12-year-old children of different ethnic and socio-economic status resident in Leicestershire and Rutland. 2. To examine whether the intra-Asian differences previously found in the primary dentition of 5-year-olds continue into the permanent dentition. | Visual method  The ethnic origin of each child was assessed visually by the examiner according to criteria used by Todd and Dodd (1985).  National Statistics Census, 2001 gives the ethnic background in localities.  Caucasian- 1,379  South Asian-316  (Hindu, Sikh and Muslim) and Others | Random sampling  12 year old children attending all 62 secondary schools across Leicestershire and Rutland.  A 20% sample was randomly selected to represent 12 year old children attending all 62 secondary schools across Leicestershire and Rutland. | Townsend Index  Low (-5.2 to >-1.1)  Average (-1.0 to ->1.99)  High (2.0 to ->8.94). | Age | 12 year | Cross- sectional study.  Inter-ethnic comparisons made.  Comparisons with host population made. |
|  |  |  |  |  |  | n  Sample size | 1753 children |  |
|  |  |  |  |  |  | Migrant/ Host  n (%) | Migrants-374  (21.4 %)  Host- 1,379  (78.6 %) |  |
| **United Kingdom 8** | (2005) Alkhatib MN, et al [8]  *Ethnic variations in orthodontic treatment need in London*  *School children* | 1. To explore the need for orthodontic treatment in a multiethnic community. 2. To assess whether the need for orthodontic treatment in ethnic minorities differs from the white population based on the dental health component and on the aesthetic component. 3. To test the agreement between normative and perceived need for orthodontic care across all ethnicities. | No information was provided on how ethnicity was defined.  Whites-54%  Blacks-12%  Asians (Indians)- 25%  Chinese- 4%  Mixed- 6% | Not clear about the sampling method  14/27 schools in the two boroughs were selected using a one to one simple sampling technique.  The required sample size for each ethnic group was based on our pilot study, a total of 3500 children. | Not mentioned. | Age | 12-14 year | Cross- sectional  Study  Comparisons with host population made. |
|  |  |  |  |  |  | n  Sample size | 2788 children |  |
|  |  |  |  |  |  | Migrant/ Host  n (%) | Migrants-1283  (46 %)  Host- 1,505  (54 %) |  |
| **United Kingdom 9** | (2007) Conway DI, et al [9]  *Dental caries in 5-year-old children attending multi-ethnic schools in Greater Glasgow – the impact of ethnic background and levels of deprivation* | 1. To examine levels of dental caries of 5-year-olds attending multi-ethnic schools in Greater Glasgow. 2. To explore the effects of deprived backgrounds and ethnic identity on their dental health. | School records  Head teachers and class teachers of the children were asked to detail the child’s religion, ethnic background and the ability of the mother to speak and understand English from their school records.  Whites-335 (51.6%)  Pakistani-215 (33.1%)  Indian -24 (3.7%)  Chinese -7 (1.1%)  African -5 (0.8%)  Arab -7 (1.1%)  Mixed origin -28 (4.3%)  Unknown -26 (4.0%) | Random sampling  The Education Department supplied a list of all schools (n =21) in Greater Glasgow with a minority ethnic population of 25% and over.  All 21 schools approached returned the requested lists.  However, two schools were excluded as they had no minority ethnic children within the Primary 1 classes. | The influence of deprivation and ethnic origin was explored, with the DEPCAT categories split into three broad groups. | Age | 5- 6 years | Cross- sectional study.  Comparisons with host population made. |
|  |  |  |  |  |  | n  Sample size | 649 children |  |
|  |  |  |  |  |  | Migrant/ Host  n (%) | Migrants-314  (48.4 %)  Host- 335  (51.6 %) |  |
| **United Kingdom 10** | (2008) Hullah E, et al [10]  *Self-reported oral hygiene habits, dental attendance and attitudes to dentistry during pregnancy in a sample of immigrant women in North London* | To describe self-reported oral health, oral hygiene habits, frequency of visits to a dentist and factors associated with dental attendance among pregnant women at a North London Hospital, the majority of whom are immigrants. | No information was provided on how ethnicity was defined.  Black African- 63 (30.6%)  White European -36 (17.5%)  White British -26 (12.6%)  Turkish- 22 (10.7%)  Black Caribbean’s- 16 (7.8%)  Black British- 13 (6.3%)  Bangladeshi- 8 (3.9%)  Indians-7 (3.4%)  Sri Lankan- 4 (1.9%)  Arab- 2 (1%) | Convenience sampling  Women on the postnatal ward of North London Hospital were invited to complete a questionnaire within 3 days of delivery. | Office of Population Censuses and Surveys which divides the population into 5 social classes (I–V), depending on occupation, skill level and social standing in the community | Age | 28.2 ± 6.2 years | Cross- sectional study.  No comparisons with host population made. |
|  |  |  |  |  |  | n  Sample size | 206 women |  |
|  |  |  |  |  |  | Migrant/ Host  n (%) | Not applicable |  |
| **United Kingdom 11** | (2011) Reekie T. [11]  *The effect of South Asian ethnicity on satisfaction with primary cleft lip and or palate repair.* | To evaluate the effect of South Asian ethnicity on satisfaction with cleft lip and or palate repair, post primary repair. | Hospital records  South Asian was defined as a person who, irrespective of birthplace, would identify him/ herself as racially and ethnically originating from peoples indigenous to India, Pakistan, Bangladesh and Sri Lanka’.  Caucasian-95  Asian-15 | Registry based sampling  All the subjects attended, or still attend, the West Midlands, UK adult cleft clinic and had undergone primary cleft lip and palate repair. Their information was obtained from electronic patient records. | Townsend index was used to record material deprivation | Age | 16-65 Years | Cross- sectional study- Registry based.  Comparisons with host population made. |
|  |  |  |  |  |  | n  Sample size | 110 adults |  |
|  |  |  |  |  |  | Migrant/ Host  n (%) | Migrants-15  (13.7 %)  Host- 95  (86.3 %) |  |
| **United Kingdom 12** | (2013) Marcenes W, et al [12]  *Ethnic disparities in the oral health of three- to four-year-old children in East London* | To assess ethnic differences related to caries experience in three- to four-year-old nursery children living in three of the most deprived boroughs in the UK- Tower Hamlets, Hackney and Newham (East London). | School records although no information was given on how ethnicity was defined.  White British-10.94%  White Eastern European-1.94%  White Other-2.74% Black African-15.6% Black Other-7.30% Indian-7.00% Bangladeshi-30.11% Pakistani -6.36%  Asian Other-5.14% Middle Eastern -4.04% Mixed-2.86%  Others-1.40% Unclassified-4.63% | Clustered sampling  Three independent random samples of 3-4-year-old children in Hackney, Tower Hamlets and Newham were selected. | Deprivation scores based on the 2004 Index of Multiple Deprivation.  Almost all (99%) children in the sample lived in the two most deprived quintiles in England. | Age | 3-4 year | Cross- sectional study.  Comparisons with host population made. |
|  |  |  |  |  |  | n  Sample size | 1285 children |  |
|  |  |  |  |  |  | Migrant/ Host  n (%) | Migrants-1144  (89 %)  Host- 141  (11 %) |  |
| **United Kingdom 13** | (2013) Al-Haboubi M, et al [13]  *Inequalities in the use of dental services among adults in inner South East London* | 1. To explore whether there are inequalities in the use of dental services among adults residing in a socially deprived, ethnically.   diverse metropolitan area,   1. To explore satisfaction with services provided, 2. To explore public perceptions on possible areas for improvement of local services. | Self-assessed ethnicity adaptation of the  2001 UK Census, which included 15 possible categories under five main ethnic groups:  Whites-466  Blacks- 193  Asians-36 | Stratified multistage random sampling was used to select a representative, ethnically diverse sample residing in the three boroughs- Lambeth, Southwark and Lewisham boroughs. | Indicated by the social grade of the chief income earner, based on their current job or occupationemployment status, size of organization, and supervisory status. | Age | 16 years and above | Cross- sectional study.  Comparisons with host population made. |
|  |  |  |  |  |  | n  Sample size | 695adults |  |
|  |  |  |  |  |  | Migrant/ Host  n (%) | Migrants-229  (33 %)  Host- 466  (67 %) |  |
| **United Kingdom 14** | (2014) Choa RM,et al [14]  *Identifying the effect of cleft type, deprivation and ethnicity on speech and dental outcomes in UK cleft patients: A multi-centered study* | To study the two outcome measures (dmft and cleft speech characteristics) that might be significantly influenced by the demographics of the populations studied independent of the care provided. | Hospital records based on self-reported information collected by the hospital administration systems  Caucasian-224  Asian-21  Mixed-8  Other-21  Unknown-13 | Secondary data through registries  3 UK regional cleft centres, West Midlands, South West/South Wales, and Spires collect and compare outcome data annually for inter centre audit. | Deprivation measure Carstairs index, developed by Carstairs and Morris in the 1980s. | Age | 5-6 year | Cross- sectional study-  Registry based.  Comparisons with host population made. |
|  |  |  |  |  |  | n  Sample size | 287 children |  |
|  |  |  |  |  |  | Migrant/ Host  n (%) | Migrants- 63  (22 %)  Host- 224  (78 %) |  |
| **United Kingdom 15** | (2015) Delgado-Angulo EK, et al [15]  *Ethnic inequalities in dental caries among adults in East London* | 1. To determine whether there were ethnic disparities in dental caries among adults living in a deprived area of the UK and exposed to the same environmental factors. 2. To explore whether socioeconomic position (SEP) measures could explain ethnic differences in dental caries. | Self-assessed ethnicity  White British-565  White East European-72  White Others-88  Black African-282  Black Caribbean-88  Black Other-135  Pakistani-202  Indian-118  Bangladeshi-75  Asian Other-325  Mixed-28  Other-35 | A multi-stage stratified random sampling approach was used to select a representative sample of the ethnically diverse general non-institutionalized population in ONEL- Outer North East London.  (ELOHI) Study- 2009–10. | The IMD (Index of Multiple Deprivation) is a census area-level measure made up of seven domain indices of deprivation | Age | 16–65 years | Cross- sectional study.  Inter-ethnic comparisons made.  Comparisons with host population made. |
|  |  |  |  |  |  | n  Sample size | 2013 adults |  |
|  |  |  |  |  |  | Migrant/ Host  n (%) | Migrants-1448  (72 %)  Host- 565  (28 %) |  |
| **United Kingdom 16** | (2016) Delgado-Angulo EK, et al [16]  *Ethnic Inequalities in Periodontal Disease among British Adults* | 1. To determine whether there were ethnic disparities in periodontal disease among adults in East London (UK) 2. Whether SEP measures could explain ethnic differences in periodontal disease. | *This study uses the same methodology as ) Delgado-Angulo EK, et al.2015*  White British-533  White East European-63  White Others-85  Black African-274  Black Caribbean-84  Black Other-131  Pakistani-198  Indian-112  Bangladeshi-69  Asian Other-314  Mixed-28  Other-34 | *This study uses the same methodology as ) Delgado-Angulo EK, et al.2015*  A multi-stage stratified random sampling. | *This study uses the same methodology as ) Delgado-Angulo EK, et al.2015* | Age | 16–65 years | Cross- sectional study.  Inter-ethnic comparisons made.  Comparisons with host population made. |
|  |  |  |  |  |  | n  Sample size | 1925 adults |  |
|  |  |  |  |  |  | Migrant/ Host  n (%) | Migrants-1392  (72.4 %)  Host- 533  (27.6 %) |  |
| **United Kingdom 17** | (2017) Abdelrahim R, et al [17]  *Ethnic Disparities in Oral Health Related Quality of Life among Adults in London, England* | To determine whether there are ethnic disparities in oral health related quality of life among adults from London (England) and the role that socioeconomic factors play in that association. | Self-assessed ethnicity using an adaptation of the 2001 UK census:  White (British, Irish), Asian (Indian, Bangladeshi, Pakistani or other)  Black (Black African, Caribbean) or Other  White-478  Black-193  Asian-34 | A stratified multi-stage random sample was used to select a representative, ethnically diverse sample of the resident adult population of Lambeth, Southwark and Lewisham boroughs. | Social grade of the chief income earner, based on their current job or occupationemployment status, size of organization and supervisory status- 6 classes identified. | Age | 16 years and older | Cross- sectional study.  Comparisons with host population made. |
|  |  |  |  |  |  | n  Sample size | 705 adults |  |
|  |  |  |  |  |  | Migrant/ Host  n (%) | Migrants-227  (28.1 %)  Host- 478  (71.9 %) |  |
| **United Kingdom 18** | (2017) Arora G, et al [18]  *Ethnic differences in oral health and use of dental services: cross-sectional study using the 2009 Adult Dental Health Survey.* | To determine whether there are ethnic differences in adult oral health in the UK and, if so, whether these persist following adjustment for differences in sociodemographic factors, lifestyle, or use of dental services. | Self-assessed ethnicity from the records  Whites-10,435 (94.6%)  Indian-272 (2.5 %)  Pakistani or Bangladeshi-165  (1.5 %)  Black-187 (1.7 %).  Data were collected during two ten week periods: October to December 2009 and January to April 2010. | From the records- two stage cluster sampling  253 primary sampling units were identified in England and Wales and another 15 in Northern Ireland giving a total sample size of 13,400 households. Recruitment was not stratified by ethnic group and there was no boosted sampling of ethnic minority groups. 60% responded. | Postcode of residence was used to derive the Index of Multiple  Deprivation (IMD). | Age | 16 years and older | Cross- sectional study-  Registry based  Comparisons with host population made. |
|  |  |  |  |  |  | n  Sample size | 11,059 adults for interview and 6492 underwent examination |  |
|  |  |  |  |  |  | Migrant/ Host  n (%) | Migrants-624  (5.6 %)  Host- 10,435  (94.4 %) |  |
| **United Kingdom 19** | (2018) Weston-Price S, et al [19]  *A multi-variable analysis of four factors affecting caries levels among five-year-old children ; deprivation, ethnicity, exposure to fluoridated water and geographic region.* | To assess the role of four factors which have been posited to affect population caries levels: deprivation, exposure to fluoridated water, region and ethnicity, utilising a representative national sample of 5 year old children across England, employing multivariable regression to assess their individual contribution to oral health inequalities. | Through school records  Whites-90,426 (74.2%)  Asian-12,992 (10.7%)  Blacks-5,822 (4.8%)  Eastern European-917 (0.8%)  Mixed-5,558 (4.6%)  Other Ethnic Group-2,464 (2.0%)  Arabic / Turkish-264 (0.2%)  Ethnic group not provided-3,432 (2.8%) | Random sampling  As part of Public Health England’s National Dental Epidemiology  Programme (NDEP), standardised examinations of random samples of five-year-old children were undertaken in the 2014/15 academic year were taken. | The home postcodes of the volunteer children were used to assign national index of multiple deprivation (IMD) quintiles. | Age | 5 year old | Cross- sectional study.  Comparisons with host population made. |
|  |  |  |  |  |  | n  Sample size | 121,875 children |  |
|  |  |  |  |  |  | Migrant/ Host  n (%) | Migrants-31,449  (25.8 %)  Host- 90,426  (74.2 %) |  |
| **United Kingdom 20** | (2018) Delgado-Angulo E.K, et al [20]  *Ethnicity, migration status and dental caries experience among adults in East London.* | To assess the interrelationship between ethnicity, migration status and dental caries experience among adults in East London.  Analysis of data from the East London Oral Health Inequality (ELOHI) Study, which included adults 16-65 years old living in Waltham Forest, Redbridge and Barking and Dagenham in 2009-2010. | Self-assessed ethnicity  Using an adaptation of the UK Census 2001 categories; including 26 ethnic subgroups organised in 5 main groups (White, Asian, Black, Mixed and Other).  UK born (host population)-874 Foreign born (migrants)-1036 | A multistage stratified random sampling approach  55 addresses were randomly selected to yield 3193 addresses. A total of 457 addresses were excluded. The final sampling frame included 2528 valid addresses and 1437 households agreed to participate in the study. | Education and the National Statistics Socio-Economic Classification (NS-SEC) were the SEP indicators. | Age | 16 years and above | Cross- sectional study.  Comparisons with host population made. |
|  |  |  |  |  |  | n  Sample size | 1910 adults |  |
|  |  |  |  |  |  | Migrant/ Host  n (%) | Migrants-1036  (54.2 %)  Host- 874  (45.8 %) |  |
| **United Kingdom 21** | (2018) Rouxel P, et al [21]  *Socioeconomic and ethnic inequalities in oral health among children and adolescents living in England, Wales and Northern Ireland* | To assess the association between ethnicity, SEP and oral health differs in childhood and mid-adolescence. | From school records, which used parents’ reporting of family ethnic group when their child started at school, and was assessed using the 2011 UK census ethnic categories:  White British/Irish- 7081  Other White-302  Mixed White (White & Black Caribbean/ African/Asian)-250  Indian-142  Pakistani- 257 Bangladeshi- 174  Black African-171 Black Caribbean-164 | A multistage cluster random sampling | Free school meals eligibility (at the child level): a proxy for family level relative income deprivation | Age | 5, 8, 12 and 15 years | Cross- sectional study.  Comparisons with host population made. |
|  |  |  |  |  |  | n  Sample size | 8541  children |  |
|  |  |  |  |  |  | Migrant/ Host  n (%) | Migrants-1460  (17.1 %)  Host- 7081  (82.9 %) |  |
| **United Kingdom**  **Oral Cancer 1** | (2000) Khan FA,et al [22]  *Predictors of tobacco and alcohol consumption and their relevance to oral cancer control amongst people from minority ethnic communities in the South Thames health region, England* | To identify possible predictors of smoking, drinking and chewing behaviours of people from minority ethnic communities living in the South Thames Health region of England. | Self-assessed ethnicity  Indian-239  Bangladeshi-103  Black- Caribbean-162  Black –African-142  Chinese-169  Pakistani-48 | Network sampling method was used.  Lists of ethnic minority community, groups were obtained from health authorities, health promotion units and councils for racial equality within the South Thames region along with advertisements through community newsletters, newspapers and community radio stations. | Education was dichotomized by whether or not participants had received full-time education up to their 16th year or beyond.  Employment level was also assessed. | Age | Into 2 groups:  ≤ 45 years and ≥ 45 years | Cross- sectional study.  Inter-ethnic comparisons made.  No comparisons with host population made. |
|  |  |  |  |  |  | n  Sample size | 1113 participants |  |
|  |  |  |  |  |  | Migrant/ Host  n (%) | Not applicable |  |
| **United Kingdom**  **Oral Cancer 2** | (2000) Vora A.R, et al [23]  *Alcohol, tobacco and paan use and understanding of oral cancer risk among Asian males in Leicester* | 1. To determine use of alcohol, tobacco and paan among males from the various Asian communities in Leicester; 2. To assess their knowledge and attitudes towards oral cancer risk factors and prevention. 3. To determine any differences regarding habits and attitudes between first and second generation Asians. | Self-assessed ethnicity  Participants were classified as 2 generation, if they were born and lived in the United Kingdom for all of their life; or, if born outside UK had been living in the country before the age of 16 years and had received primary and secondary school education in UK.  Asian (Hindu, Sikh, Muslim, Jain- based on their religion) | Convenience sampling  Asian males were recruited from GP surgeries, sixth form colleges, places of worship and shopping centres in Leicester area. | Not Recorded | Age | 16 years and above | Cross- sectional study.  Inter-ethnic comparisons made.  No comparisons with host population made. |
|  |  |  |  |  |  | n  Sample size | 524 males |  |
|  |  |  |  |  |  | Migrant/ Host  n (%) | Not applicable |  |
| **United Kingdom**  **Oral Cancer 3** | (2001) Farrand P, et al [24]  *Prevalence, age of onset and demographic relationships of different areca nut habits amongst children in Tower Hamlets, London* | 1. The extent to which the areca nut habit is being continued within the South Asian population amongst children who are predominantly first or second generation UK born. 2. The extent to which the habit is extending amongst children with other ethnic backgrounds. | Self-assessed ethnicity  Bangladeshi-498  Asians-92  British-82  Pakistani-16  Others -16 | Convenience sampling  Children were recruited from 2 secondary schools within Tower Hamlets. | Tower Hamlets is rated as the 2^nd^ most deprived country, with the largest Bangladeshi population in the UK residing there. | Age | Between 11-15 Years | Cross- sectional study.  Inter-ethnic comparisons made.  No comparisons with host population made |
|  |  |  |  |  |  | n  Sample size | 704 children |  |
|  |  |  |  |  |  | Migrant/ Host  n (%) | Not applicable |  |
| **United Kingdom**  **Oral Cancer 4** | (2001) Pearson N, et al [25]  *Prevalence of oral lesions among a sample of Bangladeshi medical users aged 40 years and in Tower Hamlets, UK* | 1. To collect data on the prevalence of oral lesions among Bangladeshi medical care users aged 40 years and over. 2. To explore the relationship of oral disease status with tobacco smoking and paan chewing. | Self-assessed ethnicity  Bangladeshi | Convenience sampling  General medical Practices in Tower Hamlet that had a large attendance of Bangladeshi adults who were identified through consultation with local healthcare organizations and providers. | Not reported | Age | 40 years and above | Cross- sectional study.  No comparisons with host population made. |
|  |  |  |  |  |  | n  Sample size | 185 adults. |  |
|  |  |  |  |  |  | Migrant/ Host  n (%) | Not applicable |  |
| **United Kingdom**  **Oral Cancer 5** | (2001) Prabhu NT, et al [26]  *Betel quid chewing among Bangladeshi adolescents living in East London* | 1. To assess the prevalence of betel quid chewing habit amongst adolescents belonging to Bangladeshi backgrounds. 2. To explore possible determinants of this habit to help in informing health promotion and prevention measures targeting this community in particular in East London. | Self- assessed ethnicity was defined by selecting people from Bangladeshi community centres.  Bangladeshi | Convenience sampling  Using snowball sampling, Bangladeshi participants were recruited from 2 out of 4 randomly selected Bangladeshi community centres in East London. | Not recorded | Age | 12-18 years | Cross- sectional study.  No comparisons with host population made. |
|  |  |  |  |  |  | n  Sample size | 204 teenagers |  |
|  |  |  |  |  |  | Migrant/ Host  n (%) | Not applicable |  |
| **United Kingdom**  **Oral Cancer 6** | (2013) Csikar J,et al [27]  *Incidence of oral cancer among South Asians and those of other ethnic groups by sex in West Yorkshire and England, 2001–200* | To report the incidence of oral cancer in South Asians compared with other ethnic groups in West Yorkshire between 2001 and 2006. | Nam Pehchan (a name recognition package) was then used to assign ethnicity  Patients were classified as either South Asian (Indian, Pakistani, Bangladeshi and other South Asian) or other.  South Asian-138  Others-2019 | Secondary data collection  Cases of oral pharyngeal cancer diagnosed between 2001 and 2006 in West Yorkshire were identified by the Northern and Yorkshire Cancer Registry. | Not Mentioned | Age | Adults (age- not mentioned) | Cross sectional study- Registry based.  Inter-ethnic comparisons made. |
|  |  |  |  |  |  | n  Sample size | 2157 cases |  |
|  |  |  |  |  |  | Migrant/ Host  n (%) | Not applicable |  |
| **United Kingdom**  **Oral Cancer 7** | (2013) Siddique I, et al [28]  *The impact of a community-based health education programme on oral cancer risk factor awareness among a Gujarati community* | To explore oral cancer risk awareness and behaviour among first and second generation Muslim Guajarati in the UK. | Not reported how ethnicity was recorded  1^st^ generation Asians: foreign born citizens or residents who have immigrated and been naturalized in the UK.  2^nd^ generation Asians: 2^nd^ generation of a family to inhabit but the first to be naturally born in the UK. | Convenience sampling  Muslim Gujarati participants aged 16 years or over were voluntarily recruited at an annual health fair organized by a community welfare group in West Yorkshire. | Not Mentioned | Age | 16 years and above | Cross sectional study.  No comparisons with host population made. |
|  |  |  |  |  |  | n  Sample size | 96 adults |  |
|  |  |  |  |  |  | Migrant/ Host  n (%) | Not applicable |  |
| **United Kingdom**  **Oral Cancer 8** | (2016) Merchant R, et al [29]  *Oral cancer awareness in young South-Asian communities in London* | 1. To evaluate awareness of oral cancer amongst the young London South-Asians and identify any aspects of knowledge about oral cancer that are lacking. 2. To determine whether demographic factors or health-related behaviours are associated with knowledge of oral cancer. | Self-assessed ethnicity  Indian-152  Pakistani-32  Bangladeshi-13  Sri Lankan-3  Malaysian-1 | Convenience sampling  Participants were recruited from 3 community centres and places of worship across London with a high concentration of the South Asian residents (Upton Park, Wembley and Southall). | Education levels were recorded. | Age | 18-44 years | Cross sectional study.  Inter-ethnic comparisons made.  No comparisons with host population made. |
|  |  |  |  |  |  | n  Sample size | 201 adults |  |
|  |  |  |  |  |  | Migrant/ Host  n (%) | Not applicable |  |
| **Germany1** | (2002) Ugur ZA, et al [30]  *Utilisation of dental services among a Turkish population in Witten, Germany.* | 1. To describe the oral health status and the dental service use pattern of a typical Turkish population in a German industrial city. 2. To assess to what extent this pattern of use could be explained by selected socio-demographic and attitudinal variables and oral health status. | Not reported how ethnicity was recorded but self-assessed  Turkish population through the sampling procedure were obtained. | Convenience sampling  3 out of 6 Turkish clubs in this town randomly chosen and all club members were included (n=258).  2 out of 5 schools with maximum Turkish students were taken (n=98).  72 out of 288 households were included in the systematic sampling (n=176) | Not given | Age | 12 -55 years | Cross sectional study.  Comparisons with host population made through Second and third German  Oral Health Study (DMS II,1991 and DMS III, 1999) |
|  |  |  |  |  |  | n  Sample size | 532 participants |  |
|  |  |  |  |  |  | Migrant/ Host  n (%) | Not mentioned |  |
| **Germany2** | (2003) Kühnisch, J, et al [31]  *Comparative Study on the Dental Health of German and Immigrant 8- to 10-Years Olds in the Westphalian Ennepe-Ruhr District* | To assess the dental health status and the caries pattern of first permanent molars in German and immigrant children. | Not reported how ethnicity was recorded but self-assessed  Definition of migrant child: foreign nationality.  Germans-300  Migrants-69 | Sampling method not described. Sample is ca. 10% of all children in one school year in the given area ( Ennepe-Ruhr-Kreis). | Not mentioned | Age | 8 years (and 9.8 years at follow-up) | Longitudinal study.  Comparisons with host population made. |
|  |  |  |  |  |  | n  Sample size | 369 children |  |
|  |  |  |  |  |  | Migrant/ Host  n (%) | Migrants-69  (18.7 %)  Host- 300  (81.3 %) |  |
| **Germany3** | (2004) Van Steenkiste M, et al [32]  *Access to Oral Care and Attitudes to the Dentist by German and Turkish Parents* | To collect information on the attitude towards dentists and access to dental care by German and Turkish parents. | Self-assessed ethnicity parents were asked in a questionnaire.  A person was considered to belong to the Turkish cultural group if his/her nationality was Turkish or if they spoke Turkish to their children at home. | Stratified random sampling  25 out of 90 elementary schools were randomly chosen. From each school, 50% of all planned medical appointments for the children were randomly chosen. Parents showing up to these appointments were questioned. | Not mentioned | Age | Adults with no age group given | Cross- sectional study.  Comparisons with host population made. |
|  |  |  |  |  |  | n  Sample size | 778 adults |  |
|  |  |  |  |  |  | Migrant/ Host  n (%) | Migrants-109  (14.1 %)  Host- 669  (85.9 %) |  |
| **Germany4** | (2004) Van Steenkiste M,et al [33]  *Prevalence of Caries, Fissure Sealants and Filling Materials among German Children and Children of Migrants* | To collect information on dental health and dental care of German children and children of migrants in the Rems-Murr-district. | Self-assessed ethnicity  Children were asked which language they spoke at home and where their parents were from.  Children with only one foreign parent were classified as German. | All were included- no sampling  All first and fourth degree children in primary and special schools were examined in the Rems-Murr-district.  6-7 year old= 4094  9-10 year old= 4002 | Not mentioned | Age | 6 -7 year old and  9 - 10 year old | Cross- sectional study.  Comparisons with host population made |
|  |  |  |  |  |  | n  Sample size | 8096 children |  |
|  |  |  |  |  |  | Migrant/ Host  n (%) | Migrants-1494  (18.4 %)  Host- 6602  (81.6 %) |  |
| **Germany5** | (2007) Schenk, L, et al [34]  *Oral health behaviour of children and adolescents in Germany. First results of the German Health Interview and Examination Survey for Children and Adolescents (KiGGS)* | To determine the prevalence and the social factors influencing selected aspects of oral health behaviour based on data of the German Health Interview and Examination Survey for Children and Adolescents (KiGGS). | Not reported how ethnicity was recorded but self-assessed  Definition of migrant/ migrational background:  1.Children who emigrated from another country themselves and at least one parent was not born in Germany or 2. Child with both parents immigrants or without German citizenship. | Stratified random sampling  Two steps: first, 167 study locations (sample points) were chosen; second, subjects were selected from the official registers of residents of the local residents' registration offices. | Separate analysis for low, medium and high SES, based on education, professional qualifications, occupational status and household income statements. | Age | 0-17 years | Cross- sectional study.  Comparisons with host population made. |
|  |  |  |  |  |  | n  Sample size | 17.641 children |  |
|  |  |  |  |  |  | Migrant/ Host  n (%) | Not reported |  |
| **Germany6** | (2007) Heinrich-Weltzien R, et al [35]  *Dental health in German and Turkish school children--a 10-year comparison* | To assess the changes in dental health in 12- and 15-year-old Turkish immigrants and German students who were included in a school-based caries-preventive programme for ten years (1993-2003). | Not reported how ethnicity was recorded but self-assessed.  Definition not mentioned (but probably using official definition, stating that a person has a migrational background if he/she or one of his/her parents was not born in Germany) | Sampling method not described.  Sample is ca. 10% of all children in one school year in the given area (Ennepe-Ruhr-Kreis)  1993 group: 352 (12-year olds) and 402 (15-year olds).  2003 group: 405 (12-year olds) and 477 (15-year olds) | No different SES in the immigrant group. | Age | 12 and 15 year old | Cross- sectional study.  Comparisons with host population made. |
|  |  |  |  |  |  | n  Sample size | 1636 children |  |
|  |  |  |  |  |  | Migrant/ Host  n (%) | Migrants- 292  (17.8 %)  Host- 1344  (82.2 %) |  |
| **Germany7** | (2007) Bissar AR, et al [36]  *Dental health, received care, and treatment needs in 11- to 13-year-old children with immigrant background in Heidelberg, Germany* | To evaluate the caries experience, the provided care, and the unmet treatment need in 11- to 13-year-old schoolchildren with immigration background compared to children without migration experience. | Self-assessed ethnicity questionnaire was sent to the parents specifying the country of birth of both parents and of their child.  Migrant children- whose parents (one or both) were born outside of Germany. | Convenience sampling  5 secondary schools that appeared to have high proportions of enrolled children with an migrant background were selected.  5th, 6th, and 7th grades were chosen. | All these 5 schools were located in underprivileged areas of the city. | Age | 11-13 year old | Cross- sectional study.  Comparisons with host population made. |
|  |  |  |  |  |  | n  Sample size | 502 children |  |
|  |  |  |  |  |  | Migrant/ Host  n (%) | Migrants-241  (48 %)  Host- 261  (52 %) |  |
| **Germany8** | (2017) Erdsiek F, et al [37]  *Oral health behaviour in migrant and non-migrant adults in Germany: the utilization of regular dental check-ups* | To examine whether migrant and non-migrant adults differ in the utilization of annual dental check-ups, taking into account socioeconomic and demographic aspects as well as additional contextual factors. | Not reported how ethnicity was recorded but given in records under definition-Individuals were defined as migrants if they had migrated to Germany themselves or if at least one of their parents had migrated to Germany. | Secondary analysis using data from the cross-sectional telephone survey ‘German Health Update 2010’. | SES as a categorical variable, distinguishing between high, middle and low status. | Age | 18 years and above | Cross- sectional study- Registry based.  Comparisons with host population made. |
|  |  |  |  |  |  | n  Sample size | 21,741 participants |  |
|  |  |  |  |  |  | Migrant/ Host  n (%) | Migrants-3413  (15.7 %)  Host- 18,328  (84.3 %) |  |
| **Germany9** | (2017) Brzoska P, et al [38]  *Enabling and Predisposing Factors for the Utilization of Preventive Dental Health Care in Migrants and Non-Migrants in Germany.* | To examine the role of demographic, social, behavioural, and health-related factors influence the decision of migrants to seek preventive dental health care and how these factors differ from those in non-migrants. | Through telephone records  Migrants were defined as individuals who had migrated to Germany themselves or of whom at least one parent had migrated to Germany. | Secondary data from two telephone surveys German Health Update 2009 and German Health Update 2010. | SES- low, middle, and high; based on a measure summarizing vocational educational, occupational status, and net equivalent income. | Age | 18 years and above | Cross- sectional study- Registry based.  Comparisons with host population made. |
|  |  |  |  |  |  | n  Sample size | 41,220 participants |  |
|  |  |  |  |  |  | Migrant/ Host  n (%) | Migrants- 6183  (15 %)  Host- 35,037  (85 %) |  |
| **Germany10** | (2018) Aarabi G, et al [39]  *Oral health and access to dental care – a comparison of elderly migrants and non-migrants in Germany.* | To compare oral health, access barriers to dental care, oral health behaviour and oral hygiene behaviour of elderly German residents with and without immigration background. | Not mentioned how ethnicity was recorded but self-assessed  But migrants were defined as- born in a country other than Germany.  Migrants-61  Host population -51 | Convenience sampling  Patients were enrolled at the dental outpatients’ clinic at the University Medical Centre Hamburg-Eppendorf (N = 70) and at three dental offices located in the Hamburg downtown area (N = 42). | Residency within the Hamburg metropolitan area. | Age | More than 60 years | Cross- sectional study.  Comparisons with host population made. |
|  |  |  |  |  |  | n  Sample size | 112 participant |  |
|  |  |  |  |  |  | Migrant/ Host  n (%) | Migrants-61  (54.4 %)  Host- 51  (45.6 %) |  |
| **Sweden 1** | (2000) Hjern A, et al [40]  *Dental Health and Access to Dental Care for Ethnic Minorities in Sweden* | To describe access to dental treatment in relation to the socio-economic situation and dental health. Is there equity in access to dental treatment for ethnic minorities in Sweden? | Self-assessed ethnicity Country of birth was used as sampling criteria of minorities.  Settled in Sweden whilst aged 20–44 between 1980 and 1989 and still residing in Sweden at the time of this survey (1996).  Poland-534  Chile-548  Iran-312  Turkey-495  Sweden-2452 | Random sampling method  Statistics Sweden conducts an annual Survey of Living Conditions based on a representative Sample.  3–15 years in household of the respondents in all 4 minority group also included. | Lived in big cities, rented their homes, education level was recorded. | Age | 3-15 and  16–84 years | Cross- sectional study.  Comparisons with host population made |
|  |  |  |  |  |  | n  Sample size | 4341 participants |  |
|  |  |  |  |  |  | Migrant/ Host  n (%) | Migrants-1889  (43.5 %)  Host- 2452  (56.4 %) |  |
| **Sweden 2** | (2001) Hjern A, et al [41]  *Social inequality in oral health and use of dental care in Sweden*  Part of the study by Hiern A, et al 2000 | To describe the social distribution of oral health and access to dental health services in Sweden two decades after the introduction of the national dental insurance scheme.  Are there still signs of social inequality? Are there any indications that the increased patient charges have affected oral health and use of dental care in the general Swedish population? | *Part of the study by Hiern A, et al 2000*  Uses terms like Swedish and foreign born. The definition of foreign born is not given. | *Part of the study mentioned by Hiern A, et al 2000*  Same sample size, taken from the annual Survey of Living Conditions based on representative sample.  3-15 years and 25-64 years (n=7610+ 4315 children) + 6761 (Swedish residents) | Part of the study by Hiern A, et al 2000 | Age | 3-15 and  16–84 years | Cross- sectional study  Comparisons with host population made. |
|  |  |  |  |  |  | n  Sample size | 19,574 participants |  |
|  |  |  |  |  |  | Migrant/ Host n (%) | Not mentioned |  |
| **Sweden 3** | (2004) Stecksén-Blicks C, et al [42]  *Caries Experience and Background Factors in 4-Year-Old Children: Time Trends 1967–2002* | To examine caries and some related background factors in 4-year-old children in Umeå in 2002 and to make comparisons with some of the findings from the earlier studies.  *A series of cross-sectional studies of caries and background factors such as oral hygiene habits, use of fluorides, and intake of sugary products between meals in 4-year-old children has been carried out with the same methods and criteria in Umeå, Sweden, in 1967, 1971, 1976, 1980, 1987, 1992 and 1997.* | Self-assessed ethnicity  Migrant status was considered present in a child if at least one parent was born outside Sweden with a native language other than Swedish.  *Migrant’s status is mentioned, but very briefly.* | Random sampling  All children born during the third quarter of 1998 who lived in the catchment areas of three Public Dental Health Clinics in Umeå  (n= 182/202). | Not mentioned | Age | 4 Years | Cross sectional study.  Comparisons with host population made. |
|  |  |  |  |  |  | n  Sample size | 182 children |  |
|  |  |  |  |  |  | Migrant/ Host n (%) | Migrants-9 (4.9 %)  Host- 173  (95.1 %) |  |
| **Sweden 4** | (2005) Jacobsson B , et al [43]  *Dental caries and caries associated factors in Swedish 15-year-olds in relation to immigrant background* | 1. To evaluate caries prevalence and caries associated variables in 15-year-olds whose parents had immigrated to Sweden in relation to Swedish 15-year-olds 2. To examine differences in caries prevalence in immigrant adolescents based on their length of residence in Sweden. | Not reported how ethnicity was recorded. Both parents had been born outside Sweden and if the parents were non-Swedish citizens at birth. | Convenience sampling  All 15-year-old adolescents at one public school in the city of Jonkoping, Sweden were asked to participate in the study and were divided into two groups based on their background:  Migrants and host population (143 invited). | Social classification based on the occupation of the bread-winner. | Age | 15 years | Cross sectional study.  Comparisons with host population made. |
|  |  |  |  |  |  | n  Sample size | 117 adolescents |  |
|  |  |  |  |  |  | Migrant/ Host n (%) | Migrants-51 (44 %)  Host- 66  (56 %) |  |
| **Sweden 5** | (2008) Stecksén-Blicks C,et al [44]  *Caries prevalence and background factors in Swedish 4-year-old children – a 40-year perspective* | To perform a new cross-sectional study on 4-year-old children’s caries prevalence, oral hygiene habits, sugar consumption, general health, and medication, and to make comparisons with the earlier studies and to compare caries prevalence and background factors in immigrant and non-immigrant children.  (*continuation of previous study in 2004*) | Self-assessed ethnicity Parents reported in questionnaire.  Children who had at least one parent born outside Sweden with native language other than Swedish were classified as migrant children. | Random sampling  All children born during the third quarter of 2002 living in the catchments areas of 3 public dental health clinics in Umeå. | Not mentioned | Age | 4 years | Cross sectional study.  Comparisons with host population made. |
|  |  |  |  |  |  | n  Sample size | 218 children |  |
|  |  |  |  |  |  | Migrant/ Host n (%) | Migrants-16  (7.3 %)  Host- 202  (92.7 %) |  |
| **Sweden 6** | (2010) Julihn A, et al [45]  *Migration background: a risk factor for caries development during adolescence* | To investigate the influence of the migration background of both the child and the parents on approximal caries increment during the teenage period among Swedish adolescents. | From registers of Total Population Register kept by SCB.  Parents and child’s country of birth and child’s age at migration to Sweden were taken.  Sweden- 14,160  Western Europe- 140  Eastern Europe- 315  Asia- 595  Africa- 143  South America- 185 | Secondary data from registers  Data sources at the Public Health Care Administration in Stockholm as well as from Swedish National Registers at the Central Bureau of Statistics (SCB). | Family received a social-welfare allowance, as well as disposable family income, was obtained for each family from the Total  Enumeration Income Register. | Age | 13 years, followed till 19 years | Longitudinal register-based retrospective cohort study.  Comparisons with host population made. |
|  |  |  |  |  |  | n  Sample size | 15,538 adolescents |  |
|  |  |  |  |  |  | Migrant/ Host n (%) | Migrants-1378  (8.8 %)  Host- 14,160  (91.2 %) |  |
| **Sweden 7** | (2010) Mousavi SM, et al [46]  *Nasopharyngeal and hypopharyngeal carcinoma risk* *among immigrants in Sweden* | To study the risk of nasopharyngeal and hypopharyngeal carcinomas in the first generation immigrants to Sweden. | From registers of FCD Family Cancer Database in Sweden. Based on country of birth: first-generation- Migrants were defined as those born outside of Sweden without identified parents in the Database.  2660 cases of nasopharyngeal and hypopharyngeal carcinoma in the native Swedish population and 243 cases in migrants, respectively. | Secondary data  Family-Cancer Database (FCD) has 11.8 million individuals, about 1.8 million are migrants.  Start of follow-up was defined as the birth year, the date of migration or January 1st, 1958, whichever came latest. | SES-  1,2a,2b,3 (3- lowest SES) | Age | Median age at cancer diagnosis was 55 years in migrants | Registry based retrospective design  Comparisons with host population made. |
|  |  |  |  |  |  | n  Sample size | 2903 cases |  |
|  |  |  |  |  |  | Migrant/ Host n (%) | Migrants-243  (8.3 %)  Host- 2660  (91.7 %) |  |
| **Sweden 8** | (2014) Stecksén-Blicks C, et al [47]  *Caries and background factors in Swedish 4-year-old children with special reference to immigrant status* | To study the prevalence of caries and contributing factors in 4-year-old children in Umeå using the same methods and criteria as used in earlier studies and compares with data collected between 1980–2007 to reveal changes over time.  (*continuation of previous study in 2004, 2008*) | Self-assessed ethnicity Parents reported in questionnaire.  Children who had at least one parent born outside Sweden with native language other than Swedish were classified as migrant children. | Random sampling  Children born during the third quarter of 2008 and who lived in the catchment areas of three public dental health service clinics in Umeå. | Not mentioned | Age | 4-years | Cross sectional study.  Comparisons with host population made. |
|  |  |  |  |  |  | n  Sample size | 203 children |  |
|  |  |  |  |  |  | Migrant/ Host n (%) | Migrants-16 (7.8 %)  Host- 187  (92.2 %) |  |
| **Sweden 9** | (2016) Olerud E, et al [48]  *Oral health status in older immigrants in a medium-sized Swedish city* | To investigate oral health and oral care habits among this  group of older people made up of immigrants to Sweden from countries outside the Nordic region. | Not mentioned how ethnicity was recorded.  Iran and the Horn of Africa, Central Asia, the Near East and the Balkans. | Convenience sampling  Attendees at senior social centres for older migrants organized by the municipality or by associations in the city of Uppsala,  Sweden Staff | Not mentioned | Age | Less than 60 years | Cross sectional study.  No comparisons with host population made.  Gender based comparisons made among migrants |
|  |  |  |  |  |  | n  Sample size | 42 adults |  |
|  |  |  |  |  |  | Migrant/ Host n (%) | Not applicable |  |
| **Italy 1** | (2004) Ferro R,et al [49]  *Comparison of data on Early Childhood Caries (ECC) with previous data for Baby Bottle Tooth Decay (BBTD) in an Italian kindergarten population.* | To assess the prevalence of Early Childhood Caries (ECC) among preschool children attending nursery schools.  Compare the prevalence of ECC between migrant children and those who were native born Italian. | Self- assessed ethnicity Mother was asked to report the ethnicity.  Migrant children belonged to families from Africa, East Europe, South America and Asia who had come to Italy in the previous 10 years. | Sampling method not clear.  Preschool children attending private and public schools in Italy.  Sample was selected from the ongoing epidemiological survey “From mother to child”. | Not mentioned | Age | 1-6 years | Cross- sectional study.  Comparisons with host population made through survey of 1994.  (n=401) |
|  |  |  |  |  |  | n  Sample size | 1006 children |  |
|  |  |  |  |  |  | Migrant/ Host n (%) | Not mentioned |  |
| **Italy 2** | (2007) Ferro R, et al [50]  *Preschoolers’ dental caries experience and its trend over 20 years in a North-East Italian Health district.* | 1. To describe caries occurrence in preschool children in the Health District n. 15 of Veneto region by age, gender and immigrant status. 2. To plot primary dentition dental caries trend by age over a 20 year time span. | Self- assessed ethnicity Mother was asked to report the ethnicity  The migrant group was defined as children (2^nd^ generation) with mothers of non-western origins (participants from Eastern Europe, Asia, Africa, Turkey, South and Central America), whereas host population had mothers with a western background. | Random sampling  From Health division, district no. 15, a total of 41 kindergartens were randomly chosen. All the children present in each kindergarten on the examination days were included. | Not mentioned | Age | 3-5 years | Cross- sectional study  Comparisons with host population made |
|  |  |  |  |  |  | n  Sample size | 2524 children. |  |
|  |  |  |  |  |  | Migrant/ Host n (%) | Migrants-192  (7.6 %)  Host- 2332  (92.3 %) |  |
| **Italy 3** | (2007) Ferro R,et al [51]  *Oral health inequalities in preschool children in North-Eastern Italy as reflected by caries prevalence.* | To describe the caries occurrence in preschool children in Local Health Districts 15 and 7 of the Veneto region in North-Eastern Italy and to compare the collected data for subgroups (immigrant or indigenous) by age and gender. | Self- assessed ethnicity  Mother was asked to report the ethnicity.  The migrant group was defined as children with mothers of “non-western” origins whereas the host population had mothers with western background.  Western and non- western groups were defined by same criteria as mentioned by Ferro R, e al. 2007 | Random sampling  From Health division, district no. 15 and no. 7, a total of 57 kindergartens were randomly chosen- (41 in H.D. no. 15, and 16 in H.D. no. 7) | Not mentioned | Age | 3-5 years | Cross- sectional study.  Comparisons with host population made |
|  |  |  |  |  |  | n  Sample size | 3,401 children |  |
|  |  |  |  |  |  | Migrant/ Host n (%) | Migrants-277  (8.1 %)  Host- 3124  (91.9 %) |  |
| **Italy 4** | (2007) Ferro R, et al [52]  *Prevalence and severity of dental caries in 5- and 12-year old children in the Veneto Region (Italy).* | To evaluate the occurrence of dental caries among 5- and 12-year-old children in North eastern Italy and to compare dental status between immigrants and native-born children. | Self- assessed ethnicity  Mother was asked to report the ethnicity.  Migrants are referred to as foreign born (China, former Yugoslavia and countries of North Africa and Eastern Europe) | Random sampling  Children were randomly recruited-  5 year old attending nursery school (n=260) and 12-year-old children (n=862) attending public middle schools.  Sample was selected from the ongoing epidemiological survey “From mother to child” as in study by Ferro R. et al 2004. | Not mentioned | Age | 5 and 12 years | Cross sectional study.  Comparisons with host population made |
|  |  |  |  |  |  | n  Sample size | 1122 children |  |
|  |  |  |  |  |  | Migrant/ Host n (%) | Migrants-63  (5.8 %)  Host- 1058  (94.2 %) |  |
| **Italy 5** | (2018) Petti S, et al [53]  *Betel quid chewing among adult male immigrants from the Indian subcontinent to Italy* | To assess BQ chewing prevalence in first-generation immigrants from the Indian subcontinent to Italy.  *BQ- Betel Quid chewing habit.* | Not reported how ethnicity was recorded.  First generation males from India, Pakistan, Sri Lanka, and Bangladesh. | Convenience sampling  First generation males, residing in Esquilino district of Rome were contacted in open-air markets and parks, and those who voluntarily agreed to participate were interviewed. | Not mentioned | Age | 18 years and above | Cross sectional study.  No comparisons with host population made.  Intra ethnic comparisons also made. |
|  |  |  |  |  |  | n  Sample size | 211 adults |  |
|  |  |  |  |  |  | Migrant/ Host n (%) | Not required |  |
| **Norway 1** | (2005) Skeie MS, et al [54]  *Caries patterns in an urban preschool population in Norway* | To describe the prevalence, severity and distribution of caries in a preschool population in Oslo, Norway, and to compare the findings in subgroups according to immigrant status and age. | Not reported how ethnicity was recorded.  Migration was defined as- children with mothers of non-western origin (Eastern Europe, Asia, Africa, Turkey, South and Central America) | Random sampling  Preschool children were drawn from 7 different clinics in the Public Dental Health Service in Oslo | Different SES, but no categories mentioned | Age | 3 to 5 years | Cross- sectional study.  Comparisons with host population made. |
|  |  |  |  |  |  | n  Sample size | 775 children |  |
|  |  |  |  |  |  | Migrant/ Host n (%) | Migrants-88  (11.4 %)  Host- 687  (88.6 %) |  |
| **Norway 2** | (2006) Skeie MS,et al [55]  *Parental risk attitudes and caries-related behaviours among immigrant and western native children in Oslo*  (Part of the study mentioned by 2005 Skeie MS et al.) | 1. To report on the oral health status of a group of 3- and 5-yearold children of Oslo. 2. To describe differences in parental beliefs, attitudes and behaviours towards their children’s oral health, indicating different risks for dental disease among western native and immigrant children, with the help of self-administered questionnaire. | Self-assessed ethnicity Parents filled the questionnaire.  Migrant group: if the mother was of non-western origin which meant Eastern Europe, Asia, Africa, Turkey, South and Central America. | Convenience sampling  Two types of clinics were chosen for sample procedure-  Clinics with high proportion of migrant children in their catchment  area or Clinics with substantial spread concerning  socioeconomic location. | Educational level of both parents, and used as a proxy for socioeconomic status: High, medium and low social status. | Age | 3 and 5 years | Cross- sectional study.  Comparisons with host population made.  Intra ethnic comparisons also made. |
|  |  |  |  |  |  | n  Sample size | 735 children |  |
|  |  |  |  |  |  | Migrant/ Host n (%) | Migrants-81 (11 %)  Host- 654  (88.9 %) |  |
| **Norway 3** | (2008) Skeie MS, et al [56]  *Caries increment in children aged 3–5 years in relation to parents’ dental attitudes: Oslo, Norway 2002 to 2004.*  (Follow up study from Skeie MS, et al. 2005, 2006) | To assess the relationship between parents’ dental attitudes and the caries increment in their children from the age of 3 to 5 years. | Self- assessed ethnicity Parents filled the questionnaire.  Migrant Status required that the mothers were first-generation migrants. As mentioned in the study by Skeie MS, et al. 2006. | Convenience sampling  As followed from the study by Skeie MS, et al. 2006 | As mentioned in the study by Skeie MS, et al. 2006  Educational level of both parents used | Age | 3 and 5 years | Cross- sectional study: Follow up study by Skeie MS et al 2005,2006  Comparisons with host population made.  Intra ethnic comparisons also made. |
|  |  |  |  |  |  | n  Sample size | 304 children |  |
|  |  |  |  |  |  | Migrant/ Host n (%) | Migrants-31  (10.2 %)  Host- 273  (89.8 %) |  |
| **Norway 4** | (2010) Skeie MS,et al [57]  *Tracking of parents’ attitudes to their children’s oral health-related behavior–Oslo, Norway, 2002–04*  (Follow up study from Skeie MS, et al.2005, 2006, 2008) | 1. To investigate dental beliefs and attitudes of a diverse group of parents from their children when they were aged 3 and 5 years old. 2. To identify possible mediators for a group composed of the parents with the most negative dental attitudes. | *This is a follow up of study discussed above. From 2002 to follow-up in 2004.*  Migrants meant children whose mothers were first-generation migrants from Eastern Europe, Asia, Africa, Turkey, South and Central American origin. | Convenience sampling  As followed from the study by Skeie MS, et al. 2006, 2008 | Same as above | Age | 3-5 years | Cross- sectional study. Series of cross sectional, prospective design studies.  Comparisons with host population made.  Intra ethnic comparisons also made. |
|  |  |  |  |  |  | n  Sample size | 282 children |  |
|  |  |  |  |  |  | Migrant/ Host n (%) | Migrants-31  (10.9 %)  Host- 251  (89.1 %) |  |
| **Norway 5** | (2010) Wigen TI, et al [58]  *Caries and background factors in Norwegian and immigrant 5-year-old children* | 1. To assess the caries situation among 5-year-old children in a low caries population in Norway, including the distribution and severity of caries at tooth and surface level, 2. To study associations between caries in 5-year-olds and parents’ education, national origin, oral health behaviours and attitudes. | Self-assessed ethnicity  According to mother’s and father’s country of birth-origin.  Migrants included parents born in Turkey, Asia, Africa, South America, Central America and Eastern Europe. | Random sample obtained from chosen 32 dental clinics. | Not mentioned | Age | 5 years | Cross- sectional study.  Comparisons with host population made. |
|  |  |  |  |  |  | n  Sample size | 523 children |  |
|  |  |  |  |  |  | Migrant/ Host n (%) | Migrant-31  (10.9 %)  Host- 251  (89.1 %) |  |
| **Spain 1** | (2006) Almerich Silla JM, et al [59]  *Oral health survey of the child population in the Valencia Region of Spain (2004)* | The objective of this study was to determine the evolution of oral health indices in the child population of the Valencia Region over the six-year period from 1998 to 2004. | Self-assessed through definition-Children of non-Spanish nationality whose parents were foreign and who had been living in Spain for less than 6 years were counted as foreign. | Random sample obtained from 9 classes in schools in the province of Castellón, 16 in the province of Alicante and 35 in the province of Valencia. | Social class was determined in accordance with the classification proposed by Domingo and Martos, based on the occupations of the parents, taking the highest of these as the social class of the child. | Age | 6, 12 15 years | Cross- sectional study.  Comparisons with host population made. |
|  |  |  |  |  |  | n  Sample size | 1388 children |  |
|  |  |  |  |  |  | Migrant/ Host n (%) | Migrants-6 year 38  (7.6 %)  12 year 28  (6 %)  15 year 25 (6.4%)  Host- 6 year 471 (92.4 %) 12 year 450  (94 %)  15 year 376  (93.6 %) |  |
| **Spain 2** | (2007) Almerich-Silla JM, et al [60]  *Influence of immigration and other factors on caries in 12- and 15-yr-old children* | To analyse the influence of socio-economic variables and oral health behaviour related variables on caries prevalence and experience among 12- and 15-yr-old children in the Valencia region, with specific focus on immigrants. | Self-assessed ethnicity Parents were asked in  questionnaire.  Migrant status was classified such that children of non- Spanish nationality, whose parents were foreign and who had been living in Spain for less than 4 year, were considered migrants. | Random sampling of clusters (classes in  schools) was conducted, resulting in the selection of classes in 27 schools for observations on 12-yr-old children (n=478) and for observations on 15-yr-old children (n= 401) | Social class- High, medium and low based on parental education level | Age | 12 and 15 year | Cross- sectional  study.  Comparisons with host population made. |
|  |  |  |  |  |  | n  Sample size | 879 children |  |
|  |  |  |  |  |  | Migrant/ Host n (%) | Not mentioned |  |
| **Spain 3** | (2018) Muñoz‑Pino N, et al [61]  *Comparing Oral Health Services Use in the Spanish and Immigrant Working Population* | To compare the use of oral health services- and associated factors- between the immigrant and Spanish working populations, using data from the Spanish National Health Survey of 2011–2012 (SNHS 2011–2012).  *(Source of data is secondary)* | Self-assessed ethnicity  Eurostat**-** Immigrant is a person undertaking an immigration, which means the action by which a person establishes his or her usual residence in the territory of a Member State for a period that is, or is expected to be, of at least 12 months, having previously been usually resident in another Member State or a third country). | Secondary data from Ministry of Health and Social Service and Equity department.  Three-stage, stratified  sampling process was used. | Education level and occupation status was recorded. | Age | 16 years and above | Cross- sectional study.  Registry based design  Comparisons with host population made. |
|  |  |  |  |  |  | n  Sample size | 8591 adults |  |
|  |  |  |  |  |  | Migrant/ Host n (%) | Migrants-711  (8.3 %)  Host- 7880 (91.7 %) |  |
| **Spain 4** | (2019) Valcarcel Soria R, et al [62]  *Acculturation and Dental Caries Among Children in Spain.* | To explore whether different indicator of acculturation were associated with caries experience in children of Madrid, Spain. | Self-assessed ethnicity though questionnaire sent home based on generational status:  Foreign born children with both migrant parents (first generation) Spanish born children with one or both migrant parents (second generation)  Spanish born children with both Spanish born parent (host population).  Moroccans 32%  Ecuadorians 26%  Eastern European 17%  Spanish 25% | Convenience sampling  Children who attended the Dental Clinic of the European University of Madrid (Spain) between February 2014 and July 2015. | Mentioned | Age | 6 and 17 years | Cross- sectional study.  Comparisons with host population made |
|  |  |  |  |  |  | n  Sample size | 313 children |  |
|  |  |  |  |  |  | Migrant/ Host n (%) | Migrants- 234  (75 %)  Host- 79 (25 %) |  |
| **The Netherlands 1** | (2015) Duijster D, et al [63]  *Parental and family-related influences on dental caries in children of Dutch, Moroccan and Turkish origin.* | 1. To investigate the relationship between parental and family-related factors and childhood dental caries in a sample of 5- to 6-year-old children of Dutch, Moroccan and Turkish origin. 2. To explore the relationship of parental and family-related factors with social class and ethnicity. | Self-assessed ethnicity through questionnaire sent to the parents.  Children were classified as Moroccan or Turkish if (i) both their parents were first-generation migrants or (ii) if one parent was a first-generation migrant and one parent was a second-generation migrant. | Convenience sampling  Participants (5 and 6 year old) were recruited from a large paediatric dental care centre in The Hague, the Netherlands. | Mother’s education level was used as a proxy for SES | Age | 5 and 6 years | Cross sectional study.  **Cases** -children with (dmft score ≥4),  **Controls** dmft/DMFT = 0, referred to as ‘caries free’)  Comparisons with host population made |
|  |  |  |  |  |  | n  Sample size | 92 parent–  child dyads participated in the study, including  46 cases and 46 controls. |  |
|  |  |  |  |  |  | Migrant/ Host n (%) | Migrants-57  Host- 35 |  |
| **The Netherlands 2** | (2016) van der Tas JT, et al [64]  *Ethnic Disparities in Dental Caries among Six-Year-Old Children in the Netherlands* | 1. To investigate potential differences in caries prevalence of children from ethnic minority groups compared to native Dutch children. 2. To study the associations between ethnicity and dental caries, explained by differences in SES or oral health behaviour. | Self-assessed ethnicity Mothers were asked in questionnaire.  Defined as non-Dutch if one of the parents was born in another country than the Netherlands.  Surinamese-Hindustani-152  Surinamese-Creole-154  Turkish-402  Dutch Antillean-161 Moroccan-308  Cape Verdean-172 | All population was taken.  All pregnant women in their first trimester in Rotterdam between April 2002 and January 2006 were enrolled in the study. | Mother’s age, educational level of mother, household income, marital status, child’s sex, child’s age, child’s BMI | Age | 6 years | Cross sectional study.  Part of the larger study- Generation R Study.  Comparisons with host population made |
|  |  |  |  |  |  | n  Sample size | 4,306 children |  |
|  |  |  |  |  |  | Migrant/ Host n (%) | Migrant-1349 (31.4 %)  Host- 2957 (68.6 %) |  |
| **Denmark 1** | (2003) Sundby A, et al [65]  *Oral health status in relation to ethnicity of children in the*  *Municipality of Copenhagen, Denmark* | 1. To describe the occurrence of dental caries in children of specific ethnic minority groups at different ages, 2. To describe the living conditions of children and their oral health habits, 3. To analyse whether dental caries experience of the children may be affected by cultural and behavioural factors. | Self-assessed ethnicity Parents asked in the questionnaire.  Dominant ethnic groups living in Copenhagen:  Turkish (n=160)  Pakistani (n=119)  Albanian (n=98)  Somali (n=98)  Arabian (n=156)  Danish (n=163) | Convenience sampling method (to cover the predominant foreign Nationalities | The schools chosen for the study were mainly located in areas of low socio-economic status in the Municipality of Copenhagen | Age | 3 ,5, 7, 15 years | Cross- sectional study.  Comparisons with host population made. |
|  |  |  |  |  |  | n  Sample size | 794 children |  |
|  |  |  |  |  |  | Migrant/ Host n (%) | Migrant-586  (73.8 %)  Host- 163 (26.2 %) |  |
| **Denmark 2** | (2010) Christensen LB, et al [66]  *Oral health in children and adolescents with different socio-cultural and socio-economic backgrounds* | 1. To describe the occurrence and severity of dental caries in preschool and school children in Copenhagen 2. To relate these findings to the children’s socio-cultural and   socio-economic backgrounds. | Records of Statistics Denmark: Danes, foreigners from Western countries (Nordic countries, countries within the EU, USA, Canada and Australia) and others.  The others’ category was further subdivided into **migrants**- children born outside Denmark and with both parents having foreign citizenship or having been born outside Denmark. The category “**descendants**” children born in Denmark of parents who were both migrants and descendants, and who had kept their foreign citizenship. | Secondary data  Data from Copenhagen Public Dental Care Service (Records of dental examination- dmft) was linked to National Board of Health (Demographics and cultural backgrounds) 2006 | SES was obtained from Statistics  Denmark”, a central government agency for national  Statistics. | Age | 5, 7, 12 , 15 years | Registry based, cross sectional, retrospective study.  Comparisons with host population made. |
|  |  |  |  |  |  | n  Sample size | 12,706 children |  |
|  |  |  |  |  |  | Migrant/ Host n (%) | Migrants-458 (4 %)  Host- 9058  (72 %)  Descendant- 3113 (24 %) |  |
| **Greece 1** | (2011) Gatou T, et al [67]  *Dental caries prevalence and treatment needs of 5- to 12-year-old children in relation to area-based income and immigrant background in Greece.* | 1. To examine the levels of dental caries, the dental treatment needs and the oral hygiene level of 5–12-year-old children attending public kindergarten and primary schools in Greece, 2. To improve our understanding on the influence of certain socio-demographic characteristics such as age, gender, area of residence (urban ⁄ semi-urban), area-based income levels as well as immigrant origin, on their dental health | From school records  Migrant background defined as the child had his/her father or both the parents as immigrants. | Stratified random sampling  18 kindergartens and 45 primary schools were randomly selected (using a random numbers generator) from the total list of the schools, stratified by urban and semi-urban location | Income data was obtained from ministry of economics and finance based on household income statements of 2006 and matched to postcode area of school location | Age | 5-12 years | Cross- sectional study.  Comparisons with host population made. |
|  |  |  |  |  |  | n  Sample size | 5116 children |  |
|  |  |  |  |  |  | Migrant/ Host n (%) | Migrants-739  (14.4 %)  Host- 4377 (85.6 %) |  |
| **Greece 2** | (2017) Mantonanaki. M, et al [68]  *Socio-demographic and area-related factors associated with the prevalence of caries among preschool children in Greece* | To examine dental caries prevalence in preschool children attending public kindergartens in the Athens metropolitan area and to investigate the impact of area deprivation and immigration status on the dmft score. | From school records  A child was recorded as non-Greek or migrant if his/her father was born in South eastern or Eastern Europe, Asia or Africa.  Albania- 66.7%  Rest from East European countries, Middle East and India. | Random sampling  This study uses data collected between 2009 and 2011 during a larger cross-sectional investigation-  A random cluster sample of 4-6- year old children was selected from public kindergartens in Attica. | Area deprivation based on Geo-demographic System of Attica (GSA).  Most children belonged to the urban prosperity group while 20% were from the most deprived categories (blue collar and striving). | Age | 4-6 years | Cross- sectional study.  Comparisons with host population made. |
|  |  |  |  |  |  | n  Sample size | 683 children |  |
|  |  |  |  |  |  | Migrant/ Host n (%) | Migrants-78  (11.5 %)  Host- 605 (88.5 %) |  |
| **Austria 1** | (2014) Cvikl B, et al [69]  *Migration background is associated with caries in Viennese school children, even if parents have received a higher education.* | To evaluate the influence of migration background on the DMFT, including the educational level of parents and their children’s school type. | Self-assessed ethnicity Parents were asked through questionnaire.  Migrant- if the child or at least one parent was not born in Austria (persons with foreign background. | Random sampling  Schools all over Vienna were contacted, whereupon 117 agreed to participate.  School type 1- for higher education  School type-2- for labour market | Parental education- low educational  level (no education or compulsory schooling) and high/ medium educational level | Age | 12 years | Cross- sectional study.  Comparisons with host population made. |
|  |  |  |  |  |  | n  Sample size | 736 children |  |
|  |  |  |  |  |  | Migrant/ Host n (%) | Migrants-373  (50.7 %)  Host- 363 (49.3 %) |  |

**References**

1. Robinson PG, Bhavnani V, Khan FA, et al. Dental caries and treatment experience of adults from minority ethnic communities living in the South Thames Region, UK. Community Dent Health. 2000;17(1):41–47
2. Newton JT, Khan FA, Bhavnani V, et al. Self-assessed oral health status of ethnic minority residents of South London. Community Dent Oral Epidemiol. 2000;28(6):424–434
3. Gray M, Morris AJ, Davies J. The oral health of South Asian five-year-old children in deprived areas of Dudley compared with White children of equal deprivation and fluoridation status. Community Dent Health. 2000;17(4):243–245
4. Pau A.K.H, Croucher R. Self-reported oral health status and oral-health related behaviours of a sample of Chinese elders in Inner London, UK: A pilot investigation, Int J Health Promot Educ. 2001;39(3):80-85
5. Ahmed B, Gilthorpe MS, Bedi R. Agreement between normative and perceived orthodontic need amongst deprived multiethnic school children in London. Clin Orthod Res. 2001;4(2):65–71
6. Newton JT, Corrigan M, Gibbons DE, et al. The self-assessed oral health status of individuals from White, Indian, Chinese and Black Caribbean communities in South-east England. Community Dent Oral Epidemiol. 2003;31(3):192–199
7. Dugmore CR, Rock WP. The effect of socio-economic status and ethnicity on the comparative oral health of Asian and White Caucasian 12-year-old children. Community Dent Health. 2005;22(3):162–169
8. Alkhatib MN, Bedi R, Foster C, et al. Ethnic variations in orthodontic treatment need in London schoolchildren. BMC Oral Health 2005;27(5):8
9. Conway DI, Quarrell I, McCall DR, et al. Dental caries in 5-year-old children attending multi-ethnic schools in Greater Glasgow--the impact of ethnic background and levels of deprivation. Community Dent Health. 2007;24(3):161–165
10. Hullah E, Turok Y, Nauta M, et al. Self-reported oral hygiene habits, dental attendance and attitudes to dentistry during pregnancy in a sample of immigrant women in North London. Arch Gynecol Obstet. 2008;277(5):405–409
11. Reekie T. The effect of South Asian ethnicity on satisfaction with primary cleft lip and or palate repair. J Plast Reconstr Aesthet Surg. 2011;64(2):189–194
12. Marcenes W, Muirhead VE, Murray S, et al. Ethnic disparities in the oral health of three- to four-year-old children in East London. Br Dent J. 2013;215(2):E4
13. Al-Haboubi M, Klass C, Jones K, et al. Inequalities in the use of dental services among adults in inner South East London. Eur J Oral Sci. 2013;121(3 Pt 1):176–181
14. Choa RM, Slator R, Jeremy A, et al. Identifying the effect of cleft type, deprivation and ethnicity on speech and dental outcomes in UK cleft patients: a multi-centred study. J Plast Reconstr Aesthet Surg. 2014;67(12):1637–1643
15. Delgado-Angulo EK, Bernabé E, Marcenes W. Ethnic inequalities in dental caries among adults in East London. J Public Health (Oxf). 2016;38(2):e55–e62
16. Delgado-Angulo EK, Bernabé E, Marcenes W. Ethnic inequalities in periodontal disease among British adults. J Clin Periodontol. 2016;43(11):926–933
17. Abdelrahim R, Delgado-Angulo EK, Gallagher JE, et al. Ethnic Disparities in Oral Health Related Quality of Life among Adults in London, England. Community Dent Health. 2017;34(2):122–127
18. Arora G, Mackay DF, Conway DI, et al. Ethnic differences in oral health and use of dental services: cross-sectional study using the 2009 Adult Dental Health Survey. BMC Oral Health. 2016;17(1):1
19. Weston-Price S, Copley V, Smith H, et al. A multi-variable analysis of four factors affecting caries levels among five-year-old children; deprivation, ethnicity, exposure to fluoridated water and geographic region. Community Dent Health. 2018;35(4):217–222
20. Delgado-Angulo EK, Marcenes W, Harding S, et al. Ethnicity, migration status and dental caries experience among adults in East London. Community Dent Oral Epidemiol. 2018;46(4):392–399
21. Rouxel P, Chandola T. Socioeconomic and ethnic inequalities in oral health among children and adolescents living in England, Wales and Northern Ireland. Community Dent Oral Epidemiol. 2018;46(5):426–434
22. Khan FA, Robinson PG, Warnakulasuriya KA, et al. Predictors of tobacco and alcohol consumption and their relevance to oral cancer control amongst people from minority ethnic communities in the South Thames health region, England. J Oral Pathol Med. 2000;29(5):214–219
23. Vora AR, Yeoman CM, Hayter JP. Alcohol, tobacco and paan use and understanding of oral cancer risk among Asian males in Leicester. Br Dent J. 2000;188(8):444–451
24. Farrand P, Rowe RM, Johnston A, et al. Prevalence, age of onset and demographic relationships of different areca nut habits amongst children in Tower Hamlets, London. Br Dent J. 2001;190(3):150–154
25. Pearson N, Croucher R, Marcenes W, et al. Prevalence of oral lesions among a sample of Bangladeshi medical users aged 40 years and over living in Tower Hamlets, UK. Int Dent J. 2001;51(1):30–34
26. Prabhu NT, Warnakulasuriya K, Gelbier S, et al. Betel quid chewing among Bangladeshi adolescents living in east London. Int J Paediatr Dent. 2001;11(1):18–24
27. Csikar J, Aravani A, Godson J, et al. Incidence of oral cancer among South Asians and those of other ethnic groups by sex in West Yorkshire and England, 2001-2006. Br J Oral Maxillofac Surg. 2013;51(1):25–29
28. Siddique I, Mitchell DA. The impact of a community-based health education programme on oral cancer risk factor awareness among a Gujarati community. Br Dent J. 2013;215(4):E7
29. Merchant R, Gallagher JE, Scott SE. Oral cancer awareness in young South-Asian communities in London. Community Dent Health. 2016;33(1):60–64
30. Ugur ZA, Gaengler P. Utilisation of dental services among a Turkish population in Witten, Germany. Int Dent J. 2002;52(3):144–150
31. Kühnisch J, Senkel H, Heinrich-Weltzien R. Vergleichende Untersuchung zur Zahngesundheit von deutschen und ausländischen 8- bis 10-Jährigen des westfälischen Ennepe-Ruhr-Kreises [Comparative study on the dental health of German and immigrant 8- to 10-years olds in the Westphalian Ennepe-Ruhr district]. Gesundheitswesen. 2003;65(2):96–101
32. Van Steenkiste M. Zugang zu zahnärztlichen Leistungen und Einstellung zum Zahnarzt bei deutschen und türkischen Eltern [Access to oral care and attitudes to the dentist by German and Turkish parents]. Gesundheitswesen. 2004;66(2):93–101
33. van Steenkiste M, Becher A, Banschbach R, et al. Prävalenz von Karies, Fissurenversiegelungen und Füllungsmaterial bei deutschen Kindern und Kindern von Migranten [Prevalence of caries, fissure sealants and filling materials among German children and children of migrants]. Gesundheitswesen. 2004;66(11):754–758
34. Schenk L, Knopf H. Mundgesundheitsverhalten von Kindern und Jugendlichen in Deutschland. Erste Ergebnisse aus dem Kinder- und Jugendgesundheitssurvey (KiGGS) [Oral health behaviour of children and adolescents in Germany. First results of the German Health Interview and Examination Survey for Children and Adolescents (KiGGS)]. Bundesgesundheitsblatt Gesundheitsforschung Gesundheitsschutz. 2007;50(5-6):653–658
35. Heinrich-Weltzien R, Kühnisch J, Goddon I, et al. Zahngesundheit deutscher und türkischer Schüler--Ein 10-Jahresvergleich [Dental health in German and Turkish school children--a 10-year comparison]. Gesundheitswesen. 2007;69(2):105–109
36. Bissar AR, Oikonomou C, Koch MJ, et al. Dental health, received care, and treatment needs in 11- to 13-year-old children with immigrant background in Heidelberg, Germany. Int J Paediatr Dent. 2007;17(5):364–370
37. Erdsiek F, Waury D, Brzoska P. Oral health behaviour in migrant and non-migrant adults in Germany: the utilization of regular dental check-ups. BMC Oral Health. 2017;17(1):84
38. Brzoska P, Erdsiek F, Waury D. Enabling and Predisposing Factors for the Utilization of Preventive Dental Health Care in Migrants and Non-Migrants in Germany. Front Public Health. 2017;5:201
39. Aarabi G, Reissmann DR, Seedorf U, et al. Oral health and access to dental care - a comparison of elderly migrants and non-migrants in Germany. Ethn Health. 2018;23(7):703–717
40. Hjern A, Grindefjord M. Dental health and access to dental care for ethnic minorities in Sweden. Ethn Health. 2000;5(1):23–32
41. Hjern A, Grindefjord M, Sundberg H, et al. Social inequality in oral health and use of dental care in Sweden. Community Dent Oral Epidemiol. 2001;29(3):167–174
42. Stecksén-Blicks C, Sunnegårdh K, Borssén E. Caries experience and background factors in 4-year-old children: time trends 1967-2002. Caries Res. 2004;38(2):149–155
43. Jacobsson B, Wendt LK, Johansson I. Dental caries and caries associated factors in Swedish 15-year-olds in relation to immigrant background. Swed Dent J. 2005;29(2):71–79
44. Stecksén-Blicks C, Kieri C, Nyman JE, et al. Caries prevalence and background factors in Swedish 4-year-old children - a 40-year perspective. Int J Paediatr Dent. 2008;18(5):317–324
45. Julihn A, Ekbom A, Modéer T. Migration background: a risk factor for caries development during adolescence. Eur J Oral Sci. 2010;118(6):618–625
46. Mousavi SM, Sundquist J, Hemminki K. Nasopharyngeal and hypopharyngeal carcinoma risk among immigrants in Sweden. Int J Cancer. 2010;127(12):2888–2892
47. Stecksén-Blicks C, Hasslöf P, Kieri C, et al. Caries and background factors in Swedish 4-year-old children with special reference to immigrant status. Acta Odontol Scand. 2014;72(8):852–858
48. Olerud E, Hagman-Gustavsson ML, Gabre P. Oral health status in older immigrants in a medium-sized Swedish city. Spec Care Dentist. 2016;36(6):328–334
49. Ferro R, Besostri A, Meneghetti B, et al. Comparison of data on Early Childhood Caries (ECC) with previous data for Baby Bottle Tooth Decay (BBTD) in an Italian kindergarten population. Eur J Paediatr Dent. 2004;5(2):71–75
50. Ferro R, Besostri A, Olivieri A, et al. Preschoolers' dental caries experience and its trend over 20 years in a North-East Italian Health District. Eur J Paediatr Dent. 2007;8(4):199–204
51. Ferro R, Besostri A, Meneghetti B, et al. Oral health inequalities in preschool children in North-Eastern Italy as reflected by caries prevalence. Eur J Paediatr Dent. 2007;8(1):13–18
52. Ferro R, Besostri A, Meneghetti B, et al. Prevalence and severity of dental caries in 5- and 12-year old children in the Veneto Region (Italy). Community Dent Health. 2007;24(2):88–92
53. Petti S, Warnakulasuriya S. Betel quid chewing among adult male immigrants from the Indian subcontinent to Italy. Oral Dis. 2018;24(1-2):44–48
54. Skeie MS, Espelid I, Skaare AB, et al. Caries patterns in an urban preschool population in Norway. Eur J Paediatr Dent. 2005;6(1):16–22
55. Skeie MS, Riordan PJ, Klock KS, et al. Parental risk attitudes and caries-related behaviours among immigrant and western native children in Oslo. Community Dent Oral Epidemiol. 2006;34(2):103–113
56. Skeie MS, Espelid I, Riordan PJ, et al. Caries increment in children aged 3-5 years in relation to parents' dental attitudes: Oslo, Norway 2002 to 2004. Community Dent Oral Epidemiol. 2008;36(5):441–450
57. Skeie MS, Klock KS, Haugejorden O, et al. Tracking of parents' attitudes to their children's oral health-related behavior-Oslo, Norway, 2002-04. Acta Odontol Scand. 2010;68(1):49–56
58. Wigen TI, Wang NJ. Caries and background factors in Norwegian and immigrant 5-year-old children. Community Dent Oral Epidemiol. 2010;38(1):19–28
59. Almerich Silla JM, Montiel Company JM. Oral health survey of the child population in the Valencia Region of Spain (2004). Med Oral Patol Oral Cir Bucal. 2006;11(4):E369–E381
60. Almerich-Silla JM, Montiel-Company JM. Influence of immigration and other factors on caries in 12- and 15-yr-old children. Eur J Oral Sci. 2007;115(5):378–383
61. Muñoz-Pino N, Vives-Cases C, Agudelo-Suárez AA, et al. Comparing Oral Health Services Use in the Spanish and Immigrant Working Population. J Immigr Minor Health. 2018;20(4):809–815
62. Valcarcel Soria R, Bernabé E, Somacarrera Perez ML. Acculturation and Dental Caries Among Children in Spain. J Immigr Minor Health. 2019;21(4):699–705
63. Duijster D, de Jong-Lenters M, de Ruiter C, et al. Parental and family-related influences on dental caries in children of Dutch, Moroccan and Turkish origin. Community Dent Oral Epidemiol. 2015;43(2):152–162
64. van der Tas JT, Kragt L, Veerkamp JJ, et al. Ethnic Disparities in Dental Caries among Six-Year-Old Children in the Netherlands. Caries Res. 2016;50(5):489–497
65. Sundby A, Petersen PE. Oral health status in relation to ethnicity of children in the Municipality of Copenhagen, Denmark. Int J Paediatr Dent. 2003;13(3):150–157
66. Christensen LB, Twetman S, Sundby A. Oral health in children and adolescents with different socio-cultural and socio-economic backgrounds. Acta Odontol Scand. 2010;68(1):34–42
67. Gatou T, Koletsi Kounari H, Mamai-Homata E. Dental caries prevalence and treatment needs of 5- to 12-year-old children in relation to area-based income and immigrant background in Greece. Int Dent J. 2011;61(3):144–151
68. Mantonanaki M, Hatzichristos T, Koletsi-Kounari H, et al. Socio-demographic and area-related factors associated with the prevalence of caries among preschool children in Greece. Community Dent Health. 2017;34(2):112–117
69. Cvikl B, Haubenberger-Praml G, Drabo P, et al. Migration background is associated with caries in Viennese school children, even if parents have received a higher education. BMC Oral Health. 2014;14:51
