# Supplementary material for: Oral Health Status, Oral Health Behaviours and Oral Health Care Utilisation Among Migrants Residing in Europe: A Systematic Review
Source: J Immigr Minor Health. 2020 Jul 19;23(2):373–88. doi: 10.1007/s10903-020-01056-9 (PMC7914188; doi:10.1007/s10903-020-01056-9)
Supplement: Supplementary file 3 — Supplementary file3 (DOCX 78 kb) [file 10903_2020_1056_MOESM3_ESM.docx]

**Appendix Table 3: Details of Oral health status**

| **Author/ Title of the article** | **Variables Measured**  **(Dental caries, Periodontal diseases, Bleeding gums, Orthodontic problems, other oral problems)** | **Methodology Used**  **(Measuring Criteria Used)** | **Observed Findings**  **(As reported)** |
| --- | --- | --- | --- |
| (2000) Robinson PG, et al [1]  *Dental caries and treatment experience of adults from minority ethnic communities living in the South Thames Region, UK.*  (Predictors measured in Table-4) | Clinically examined dental caries   - Edentulousness - Untreated dental caries - No dental caries (DMFT = 0)/ Caries free - Mean DMFT | Dental status (Dental Caries) was recorded using the diagnostic criteria of The British Association for the Study of Community Dentistry (BASCD). | - Edentulousness: When compared to the migrants, host population (Southern England) showed highest levels of edentulousness (57%) in the age group of 55-65 years. Overall, migrants in this study had better dental health (in terms of proportions edentulousness or with 18 or more sound teeth) than host population (Southern England) - Untreated dental caries: Pakistanis (21%) and Chinese/ Vietnamese (21%) showed the highest levels of untreated dental caries closely followed by Indians (20%) whereas Black Caribbean showed the lowest level (11%) of untreated dental caries. - Caries free: Caries free rates were also highest among Bangladeshi (44 %), and least were observed among Black Caribbean (6%) - Mean DMFT: The mean DMFT was highest among Black Caribbean (13.6) and lowest was observed among Bangladeshis (2.8). |
| (2000) Newton JT, et al. [2]  *Self-assessed oral health status of ethnic minority residents of South London* | Self-reported oral health   - Subjective symptoms - Impact of oral and facial symptoms (Impact score)   *Questionnaire was validated*  *Part of the larger study by Robinson P.G, et al 2000* | Taken from the questionnaire: Subjective Oral Health Status Indicators (SOHSI): short version  An impact score with values of zero or one, indicating absence or presence of any degree of impact. | - Self- assessed symptoms, toothache was considered most important experience by Indians (27%), followed by Bangladeshi (20%), whereas this variable was least commonly reported by Black Africans (14%) and Black Caribbean’s (15%). - Bleeding gums were considered important by Black Africans (27%) and least important by Chinese (18%). - Occurrence of pain while eating certain foods- lowest frequency was reported by Chinese and Bangladeshi adults. - Presence of sensitivity: Highest reporting by Chinese and Bangladeshi group - Appearance of teeth: No statistical difference was reported by any ethnic group. - The impact score (% percentage reporting 1 or more impact item) was highest among Black Africans (33%) and Pakistani (33%). |
| (2000) Gray M, et al [3]  *The oral health of South Asian five-year-old children in deprived areas of Dudley compared with White children of equal deprivation and fluoridation status.* | Clinically examined dental caries   - d_3_t - mt - ft - d_3_mft - untreated decay (d_3_>0) - restorative index | Dental status (Dental Caries) was recorded using the diagnostic criteria of The British Association for the Study of Community Dentistry (BASCD).  d_3_mft index (care index) was used- caries extending to dentine. | - Untreated decay: The percentage of children with untreated decay (d_3_ > 0) was significantly higher among South Asian 5-year olds (43%) as compared to host population (White children) (28%) (p<0.01). - dmft: The mean d_3_mft was significantly higher in South Asian 5 year-olds (1.43) compared to host population (White children) (1.06) (p<0.05), showing significant higher d_3_t among South Asian children (1.19) compared to White children (0.80) (p<0.01). - Dental caries experience: In the primary dentition, South Asian children had 35% more dental caries experience than host population (p<0.05) |
| (2001) Pau AKH, et al [4]  *Self-reported oral health status and oral-health related behaviours of a sample of Chinese elders in Inner London, UK: A pilot investigation*  (Use, knowledge and behaviors discussed in Table 4) | Self-reported oral health   - Perceived oral and general health - Oral symptoms and the impact of oral conditions   *Questionnaire was not validated* | Interviews were carried out using structured questionnaire adapted from the one used in the Hong Kong Adult Dental Health Survey (1994). | - Perceived oral health: 33 subjects (72%) rated the conditions of their teeth as poor or fair. - 17 subjects (32%) had 20 or more teeth and 31 subjects (57%) reported that they wore dentures. - The most common oral conditions experienced were food packing (34 subjects or 63%), loose teeth (17 or 31 %) and sensitivity (17 or 31 %) followed by toothache (15 or 28%) and mouth ulcers (13 or 24%). |
| (2001) Ahmed B, et al [5]  *Agreement between*  *normative and perceived orthodontic need amongst deprived multiethnic school children in London* | Clinically examined Orthodontic status   - IOTN AC Index of Orthodontic Needs- (Aesthetic component) using VAS- Visual Analog Scale - Self- administered questionnaire | Malocclusion was assed using the IOTN AC scale by examiner  Malocclusion was assessed using a visual analogue scale by children  Questions asked were on perceived need of braces, importance of having straight teeth , hours of television viewing per day and perceived effect of  media on self-image | - Perceived need for braces, hours of television viewing, ethnic background and social class were found to be significant for varying thresholds. - Normative needs: Black children at thresholds of the IOTN AC scale (4–6) were less likely to concur with normative treatment need compared with white or South Asian children, suggesting they perceive their dentition differently to white children when compared with a calibrated professional’s score. - IOTN AC is better suited to whites and South Asians, both of whom share similar dento-facial features. Blacks have a different cephalometric relationship and thus the IOTN AC scale may not be as well suited to them. - Using IOTN AC this study demonstrates that at various points along the scale, different influences play a significant role in agreement/disagreement between normative and perceived needs, indicating that patient–clinician agreement regards orthodontic treatment is sensitive to several cultural factors. |
| (2003) Newton JT, et al [6]  *The self-assessed oral health status of individuals from White, Indian, Chinese and Black Caribbean communities in South-east England* | Self-reported oral health   - Ability to chew. - Ability to speak. - Oral and facial pain symptoms. - Other oral symptoms. - Eating impact scale. - Activities of daily living scale. - Worry/concern scale   *Questionnaire was validated* | Taken from the Subjective Oral Health Status Indicators (SOHSI), complete version | - Presence or absence of oral and facial pain symptoms was predicted by gender and ethnicity. Women and members of the Chinese and Indian ethnic groups were more likely to report oral and facial pain symptoms - Scores on the ‘eating impact’ scale were predicted by ethnicity and employment status. Indian and Chinese respondents reported the highest levels of impact and self-assessed oral symptoms. Reported impact was generally lowest among the respondents who were identified as Black Caribbean. - Individuals from the Chinese ethnic group reported greater impact of ‘ability to chew’ and ‘ability to speak. - Indian or Chinese reported greater impact of their oral condition upon eating compared to host population (White) - Being an Indian or Chinese was more commonly related to have ‘eating impact’.   *Levels of self-reported symptoms in the present study were lower than previously reported and comparable to previous studies in the majority ethnic population (Ref 2: Newton JT, Khan FA, Bhavnani V, et al 2000). Explanation- due to the instrument used. (SOHSI- short version vs complete version).* |
| (2005) Dugmore CR, et al [7]  .  *The effect of socio-economic status and ethnicity on the*  *comparative oral health of Asian and White Caucasian 12-*  *year-old children* | Clinically examined dental caries and dental erosion   - Dental caries - DMFT - % DMFT > 0 - Dental erosion | According to the British Association for the Study of Community Dentistry (BASCD) (Pitts et al., 1998), with caries diagnosed at the caries into dentine (D_3_) threshold.  The index for evaluating tooth erosion was the one used in the Children’s Dental Health in the UK 1993 study (O’Brien,1994) | - Host population children had significantly worse dental health than migrant Asian children. - Dental caries status: Mean DMFT (1.0 among Whites compared to 0.7 among Asians) and the proportions with active decay and decay experience (32% among Whites compared to 24% among Asians) were significantly higher in host White children, as was the prevalence of tooth erosion (62% among Whites compared to 48% among Asians) (p<0.01). - Deprivation standards: Similar situation was seen among Whites and Asians belonging to equally high deprivation status- Poorer oral health among White (1.6) compared to Asians (0.7) was observed (p<0.01). Similar observations were made for dental erosions with 67% White children experiencing erosion compared to 44 % of Asians with equal deprivation. - Intra-ethnically: Comparing the mean DMFT between Asian Muslims (1.1) and non- Muslims (0.6) showed that Muslims had higher decay compared to other. When Asian Non- Muslim children from all deprivation levels were compared in mean DMFT, it was observed that high deprivation children had 0.5, average deprivation showed 0.6 and low deprivation group had 1.0 mean DMFT, overall still lesser than Asian Muslim children (1.1) - Dental caries and Deprivation: There was a significant positive association between caries and deprivation for White children, but the reverse was true for non-Muslim Asians. White Low Deprivation children had significantly less tooth erosion, but erosion experience increased with decreasing deprivation in non-Muslim Asians. - Summary: Oral health is associated with ethnicity and linked to deprivation on an ethnic basis. The intra-Asian dental health disadvantage found in the primary dentition of Muslim children is perpetuated into the permanent dentition |
| (2005) Alkhatib MN, et al [8]  *Ethnic variations in orthodontic treatment need in London*  *School children* | Clinically examined orthodontic status   - IOTN AC Index of Orthodontic Needs- (Aesthetic component) - IOTN DHC (Dental Health Component) | Dental status was recorded using the diagnostic criteria of The British Association for the Study of Community Dentistry (BASCD).  10-point analogue scale with two pictures at each end of the scale representing IOTN AC scores of 1 and 10 was used.  IOTN needs- subjective and clinical grades | - Perceived needs: Using the IOTN AC index, almost half of children (48%) scored their teeth as 2 or 3 on the index (1-4 score means no treatment need). Less than 2% had severe scores (8–10). When assessing the ethnicity, children from Black ethnic group had the least perceived need for treatment. - Normative needs: Using the IOTN AC index, the examiner scored over two thirds (69%) of children's teeth as 2 or 3 on the IOTN AC index. Differences between ethnic groups were less obvious in the professional assessment; the majority of children from all ethnic backgrounds were assessed as having low need for treatment. - Using DHC: Children from Black ethnicity (72.9%) had less need for treatment than did their White peers (69%), whereas children of Chinese (62.1%) and Indian (65.2%) ethnicities had slightly more need for treatment. - Summary: In this study ethnicity did not influence orthodontic need for treatment based on clinical or aesthetic grounds, although, children of Indian and Chinese ethnicities had a slightly higher clinical need for treatment. However treatment need based on aesthetic index continues to vary in all ethnic groups from the professional aesthetic assessment |
| (2007) Conway DI, et al [9]  *Dental caries in 5-year-old children attending multi-ethnic schools in Greater Glasgow – the impact of ethnic background and levels of deprivation* | Clinically examined  dental caries   - d_3_mft index | Following BASCD guidelines and international epidemiological conventions, figures presented for “decay” only relate to dental caries which clinically appears to have penetrated into dentine (d_3_). | - BME and host population: When combined, all of the black and minority ethnic groups (BME) together (n = 288), showed a significantly (p < 0.001) higher severity of caries in the BME (mean d_3_mft = 3.8) compared with the host population (White counterparts) (mean d_3_mft = 2.3). Also, there was a significantly (p < 0.001) lower proportion of BME children (30%) who had no obvious decay compared to the white children (48%). - Pakistani and host population: There was a significantly (p < 0.001) higher severity of caries (mean d_3_mft = 4.1; 95% CI 3.6 to 4.6) in Pakistani children compared to the host population (White children) (mean d_3_mft = 2.3; 95%CI 1.9 to 2.62). Only 25% (95% CI 17 to 34) of the Pakistani children had no obvious decay, significantly lower (p < 0.001) than the host population (white contemporaries) (48%, 95% CI 39 to 58). Similarly, significantly (p < 0.001) more White (48%) than Pakistani (25%) children had no obvious caries. - Summary: The mean d3mft was higher in Pakistani children (4.0) compared to the host population (2.5) at all levels of deprivation (under the score 6-7). Hence, Pakistani ethnic origin was associated with significantly higher levels of dental caries (p < 0.001), after adjusting for socio-economic deprivation. |
| (2013) Marcenes W, et al [12]  *Ethnic disparities in the oral health of three- to four-year-old children in East London* | Clinically examined  dental caries   - dmft index | The dental examiner adhered to the BASCD diagnostic criteria for dental caries and oral sepsis. | - Number of teeth with caries experience (dmft): Children whose parents were White European (mean dmft = 2.5), Bangladeshi (mean dmft = 1.2) and Pakistani (mean dmft = 1.3) had significantly higher dmft scores than children who had White British parents (mean dmft = 0.6). Only 18 % of children with White British parents had caries experience, compared to 50% of children with White Eastern European parents and 31% in children with Bangladeshi parents. - Number of teeth with untreated caries into dentine: The findings related to untreated dental caries were similar to the caries experience findings. Importantly, a statistically significantly higher percentage of White Eastern European (43%) children had one or more untreated carious primary tooth into dentine compared to White British children (17%). As for the Asians, 29% of the Pakistani children had one or more tooth with carries extending into the dentine. - Overall, preschool children from a White Eastern European, Bangladeshi and Pakistani background are likely to experience significantly poorer oral health than their White British counterparts |
| (2014) Choa RM,et al [14]  *Identifying the effect of cleft type, deprivation and ethnicity on speech and dental outcomes in UK cleft patients: A*  *multi-centered study* | Clinically examined dental caries and cleft   - Dental caries - Cleft determination - The Cleft Audit Protocol for Speech augmented (CAPS-A) 2 is an assessment tool for use in inter-centre. CAPS-A includes ratings of both nasal airflow errors and cleft speech characteristics (CSCs) audit | Dental caries was scored by appropriately BASCD calibrated consultants in paediatric dentistry in each cleft team.  The cleft type determined at the time of the first operation was recorded for each patient and was subdivided into five groups: unilateral cleft lip and palate (UCLP), bilateral cleft lip and palate (BCLP), cleft palate only (CP), cleft lip and alveolus (CLA) and cleft lip alone (CL). | - Based on dental caries outcome: A statistically significant relationship was demonstrated between ethnicity and dental outcomes (p = 0.002), with Asian patients having a significantly worse outcome (higher d_3_mft ≥1) than patients in either the Caucasian or ‘Other’ groups. - This study also found a significant effect of ethnicity on dmft scores of children with a cleft lip and/or palate, with children of Asian origin having significantly higher dmft scores than children categorised as either Caucasian or ‘other’ origin. - Based on cleft outcome: Cleft speech characteristics were not found to be significantly related to either deprivation scores (p =0.49) or ethnicity (p = 0.15). - Significant relationships between the variables of cleft type, deprivation and ethnicity and the outcomes of dental caries (dmft) and speech (CSCs) can be seen. |
| (2015) Delgado-Angulo EK, et al [15]  *Ethnic inequalities in dental caries among adults in East London* | Clinically examined  dental caries   - DMFT index | Visual examination using criteria used in UK Adult Dental Health Survey. | - Dental caries: Significantly lower DMFT values were observed among Asian (Pakistani mean DMFT= 5.5, Indian mean DMFT= 7.4, Bangladeshi mean DMFT= 5.7) and Black (African mean DMFT= 4.9, Caribbean mean DMFT= 8.2) migrants than each of the two White groups (British mean DMFT= 13.47, Eastern European mean DMFT= 12.18). - DMFT components: By DMFT components, Asian and Black migrants had fewer filled and missing teeth than White groups (British and Eastern Europeans). However, there were no differences in the number of decayed teeth between any groups. - All Whites compared to all Blacks and all Asians: When adjusted for SES and demographics, it was observed that while Eastern European group had a significantly higher DMFT than host population (White British) (22 and 27% higher for White East European and White Other, respectively), it was significantly lower for all Black (African 52%, Caribbean 34% and Other 32% lower than White British) and Asian groups (Pakistani 49%, Indian 41%, Bangladeshi 47% and Other 30% lower than White British). - Overall, on an average, Asians and Blacks groups exhibited lower caries experience and ethnic disparities were independent of socioeconomic factors. |
| (2016) Delgado-Angulo EK, et al [16]  *Ethnic Inequalities in Periodontal Disease among British Adults* | Clinically examined  periodontal conditions   - Pocket depth (PD) of ≥ 4 mm - Loss of attachment (LOA) ≥ 4mm | Clinical examinations were based on the protocol and diagnostic criteria of the UK Adult Dental Health Survey | - Pocket depth: The number of teeth with PD≥4 mm was significantly different among ethnic groups. Asians had significantly more teeth with PD≥ 4 mm than host population (White British). More specifically, the number of teeth with PD≥4 mm was, on average, 1.70, 1.78, 2.13 and 1.65 times higher in Pakistani, Indian, Bangladeshi and Asian Others than in host population (White British), regardless of sociodemographic background. - Loss of attachment: On the other hand, the number of teeth with LOA≥4 mm varied significantly among age groups, education level and socioeconomic classification. Compared to host population (White British), the mean number of teeth with LOA≥ 4 mm was 1.55, 2.09 and 1.77 times higher in White Eastern Europeans, Black Africans and Bangladeshis, respectively. - The association of ethnicity with periodontal disease was moderated by education, but not by socioeconomic classification. |
| (2017) Abdelrahim R, et al [17]    Community Dent Health. 2017 Jun;34(2):122-127  *Ethnic Disparities in Oral Health Related Quality of Life among Adults in London, England* | Self-reported oral health  The short version of the Oral Health Impact Profile (OHIP-14) was completed using home interviews.  *Questionnaire was validated* | Home interviews using a structured interview guide was used to measure the adverse effects of oral conditions on individuals’ life in the past 12 months under-   - The prevalence of oral impacts - The extent of oral impacts - The severity of oral impacts | - There were crude disparities by ethnicity in the extent, but not in the prevalence or severity of oral impacts. - In unadjusted regression models, Black adults reported more items affected (RR: 1.67; 95% CI: 1.20-2.33) and Asian adults fewer items affected (RR: 0.27; 95% CI: 0.08-0.87) than their White counterparts. These differences were attenuated but remained significant after adjustment for demographic factors (sex, age group and borough of residence). - Black adults showed greater and Asian adults lower prevalence, extent and severity of oral impacts than White adults. However, significant differences were only found for the extent of oral impacts; Black adults reporting more and Asian adults fewer OHIP-14 items affected than their White counterparts. After adjustments for socioeconomic factors, Asian adults had significantly fewer OHIP-14 items affected than White adults (rate ratio: 0.28; 95%CI: 0.08-0.94). - The difference in extent of oral impacts between Black and White adults was fully attenuated whereas that between Asian and White adults was attenuated but remained significant after adjusting for social grade. - Overall, ethnic groups have similar or even better oral health related quality of life (OHRQoL) than white counterparts. All three subjective measures showed that Asian adults had fewer oral impacts on quality of life due to oral conditions than White and Black adults, although these differences were significant only for the extent of oral impacts. |
| (2017) Arora G, et al [18]  *Ethnic differences in oral health and use of dental services: cross-sectional study using the 2009 Adult Dental Health Survey.*  (Questionnaire discussed in Table 3) | Secondary records   - Oral examination - Self-reported oral health | Examination included assessment of the condition of teeth surfaces, root surfaces, spaces, aesthetics and dentures, as well as a basic periodontal examination to assess the periodontal condition | - Dental caries: Dental examination of a sub-group demonstrated fewer dental caries among South Asian participants. Thus, findings show that migrants have generally better oral health, defined by the presence of more teeth, and have had correspondingly fewer dental extractions. - Self-perceived oral health: Host population (Whites) and Indian participants were more likely to rate their own oral health as either good or very good, and were the least likely to report difficulties eating due to dental problems (71.7% and 72.8% respectively). After adjusting for the potential confounding effects of age, sex, educational qualifications, household tenure, area socioeconomic deprivation quintile and area of residence, South Asian participants remained significantly less likely than White participants, to report fillings, dental extractions and having less than 20 teeth. - Almost half of Pakistani/Bangladeshi participants rated their oral health as bad or poor in spite of being least likely to report missing teeth and dentures. |
| (2018) Weston-Price S, et al [19]  Community Dent Health. 2018 Nov 29;35(4):217-222  *A multi-variable analysis of four factors affecting caries levels among five-year-old children ; deprivation, ethnicity, exposure to fluoridated water and geographic region.* | Clinically examined dental caries  Comparisons were made between dental caries, fluoridation and deprivation status of ethnic and native children | A visual-only examination method was used to assess decayed, missing and filled  teeth (d3mft), with decay measured at the dentinal level, using BASCD criteria | - Dental caries: Higher mean counts of d_3_mft in those children with decay were seen in children from Asian and Eastern European families. - Caries experience: When holding all other factors constant five-year-old children from an Eastern European ethnic group had on average, over three times the odds of having caries experience (OR 3.42, 95% CI 1.47, 7.95) than children from the other groups. This compares with Asian/Asian British children who had, on average, odds of caries experience which are more than double those of children from a White ethnic group (OR 2.51, 95% CI 2.15, 2.94). - Interaction between fluoride and ethnicity: The fluoride and ethnicity interaction term of model 1 indicates that fluoride had a bigger proportional impact (benefit) on prevalence of caries in the Asian / Asian British ethnic group when compared to those from the reference White ethnic group (OR 0.80, 95% CI 0.71, 0.90). - Interaction between fluoride and dental caries: There was a significant differential effect of water fluoridation on the severity of caries in the Asian/Asian British group when compared to the White reference group, shown by the ethnicity/fluoridation interaction term for this group in model 2 (IRR 0.90, 95% CI 0.83, 0.97). - The study described here found that ethnicity was significantly associated with the presence and severity of decay after partial adjustment for deprivation, exposure to fluoridated water and region of residence on a large national data set. |
| (2018) Delgado-Angulo E.K, et al [20]  *Ethnicity, migration status and dental caries experience among adults in East London*  (Findings on questionnaire not presented) | Clinically examined  dental caries   - DMFT index | BASCD criteria | - Ethnicity and nativity status were associated with DMFT. Blacks and Asians had lower DMFT compared to the host population whereas eastern Europeans (white others) had higher DMFT compared to the host population. - Dental caries: Association of migration status with DMFT among migrant adults showed that age at arrival and length of residence were significantly associated with DMFT. The greater the age at arrival and the longer the residence in the UK the greater the DMFT (adjusted RR: 1.03 and 1.04 per additional year). The three-way interaction between ethnicity, age at arrival and length of residence was significant (P = .002). - Inter-ethnic comparisons: Within the same ethnic groups, there was difference in dental caries based on being born in the UK or not. For example, white migrants had greater caries experience than host population of the same ethnicity. On the other hand, every Asian and Black migrant had lower caries experience than UK-born Asians and Blacks. Moreover, all UK-born Asian and Black adults had lower caries experience than UK born White British. |
| (2018) Rouxel P, et al [21]  *Socioeconomic and ethnic inequalities in oral health among children and adolescents living in England, Wales and Northern Ireland* | Clinically examined  oral health indicators   - Dental caries - Periodontal diseases - Gingivitis - Plaque | CDHS 2013 adopted the International Caries Detection and Assessment  System for assessing first staging of caries process.  Periodontal health by combining the presence of some gum inflammation (gingivitis), the presence of plaque or the presence of calculus in more than 1 sextant. | - Predicted rate of decay: At age 5 and 8, there was strong evidence of ethnic and SEP differences in tooth decay. The predicted rate of decayed teeth for White British/Irish children aged 5 was 1.54 (95%CI 1.30-1.77). In contrast, the predicted rate for Indian and Pakistani children was about 2-2.5 times higher. The predicted rate of decay was lowest among Black African children at age 5. Furthermore, Black African children tended to have lower levels of decay at all ages. In contrast, very weak evidence of ethnic differences in decay among children aged 12 and 15 was seen. - Predicted rate of filled teeth- Other White children had higher predicted rates of filled teeth compared to the White British/Irish children. However, ethnic minority children at age 15 did not have significantly higher rates of filled teeth compared to White British/Irish children. Indeed, 15-year-old Bangladeshi children had significantly lower rates of filled teeth compared to White British/Irish children. - Periodontal status- Poor periodontal health was associated with ethnicity among children aged 5 and 12, with Bangladeshi children having the highest predicted probabilities of poor periodontal health. Bangladeshi children aged 5 also had the highest predicted probabilities of gingivitis and plaque. In contrast, at age 15, there were no significant ethnic differences in poor periodontal health, gingivitis and plaque. - Overall, risk of poor oral health for ethnic minority children significantly reduces from age 5 to age 15. The ethnic differences that are clearly shown among children aged 5 are no longer apparent among children aged 15. Association between deprivation and poor oral health remains the same across different children’s ages, and in the case of poor periodontal health, this association was stronger among older children. |
| (2001) Pearson N, et al [25]  *Prevalence of oral lesions among a sample of Bangladeshi medical users aged 40 years and in Tower Hamlets, UK* | Clinically examined  oral lesions  Presence or absence of oral lesions on the soft tissues of the mouth strongly associated with paan and tobacco habits, namely: oral cancer, leucoplakia, erythroplakia, lichen planus, oral submucous fibrosis and smokers’ keratosis. | Examinations were conducted using standardized criteria by the World Health Organization, Newton et al., 1962 and Rose et al., 1968: | - 40% of participants had some oral pathology. The most common lesion, leucoplakia, was found in 25% of participants (similar in males and females). - Smoking and lesion: In a multiple logistic regression analysis adjusting for sex, age and paan chewing with or without tobacco a statistically significant association was found between the presence of any type of oral pathology and smoking tobacco (55 % of current smokers had some type of oral pathology). The odds ratio showed that participants who smoked tobacco were 3.42 times (95 % CI, 1.36 to 8.65) more likely to have some oral mucosal pathology than participants who did not smoke tobacco. - Chewing tobacco and lesion: The odds ratio for participants who chewed paan with tobacco showed that they were 5.21 times more likely to have leucoplakia than those who did not. - Leucoplakia was most frequently found on the buccal mucosa (52.1 %), the labial commissures (18.8 %) and the tongue (12.5 %). Bangladeshi medical service users in Tower Hamlets have high levels of oral pathology, particularly in relation to leucoplakia, which was related to their paan with tobacco chewing habits. |
| (2013) Csikar J,et al [27]  *Incidence of oral cancer among South Asians and those of other ethnic groups by sex in West Yorkshire and England, 2001–2006* | Secondary data   - Oral and pharyngeal cancer cases - Including Thyroid - Excluding Thyroid - Lip and mouth cancers | Cases of oral and pharyngeal cancers diagnosed between 2001 and 2006 in West Yorkshire were obtained from the Northern and Yorkshire Cancer Registry and Information Service from their database and defined by the International Classification of Disease (ICD) codes, 10th revision (codes ICS C00-C14, C30-C32 and C73),  Lip and mouth cancers (ICD 10: C00–C08) | - Oral cancers including, and excluding thyroid- Rates among women were significantly higher in South Asians than among other ethnic groups in both England and in West Yorkshire whereas, men from other ethnic groups had significantly more oral cancers than South Asian patients. - Cancers of the lip and mouth- were significantly more common among women of South Asian origin than among women of other ethnic groups. Men from other ethnic groups again had significantly higher rates of oral cancer in England as a whole compared with men of South Asian origin. - Overall- South Asian patients were at higher risk of being diagnosed with oral cancer by 79% (ratio 1.79 (95% CI: 1.31–2.47)) compared with those from other ethnic groups within West Yorkshire, adjusted for age and sex. - The results show that South Asian women had a higher incidence of oral cancer in West Yorkshire and in England as a whole than women from other ethnic groups, and this excess may be linked to the use of smokeless tobacco and to diet. |
| (2002) Ugur ZA, et al [30]  *Utilisation of dental services among a Turkish population in Witten, Germany.*  (Findings on questionnaire in Table-3) | Clinically examined oral health     - Dental caries - Missing teeth - Gingival bleeding - Periodontal pockets | WHO assessment 1987 criteria.  The periodontal examination was performed using six-point checking around all teeth, except the third molars (4mm or greater pocket depth). | - Dental caries: Missing teeth increased rapidly with increasing age. While the highest component of DMFT index was FT in the age groups of 13-14, 15-24 and 25-34 years, after the age of 34 years the number of missing teeth was found to be higher than the other components of DMFT. However, while FT was the highest component of the index in Germans, the number of filled teeth was almost equal to the number of missing teeth in Turks. No important difference between the young Turkish and German groups according to caries experience. However in older age groups the Turkish group had a lower DMFT index, but also had a lower level of treatment. - Periodontal health: The highest number of periodontal pockets was observed between 35- 44 years. - Comparisons with Second German Oral Health Study: (DMS II, 1991) shows that the 13-14 year-old Germans had more decayed teeth (DT= 2.14) than the Turkish group. The mean number of missing (MT= 0.05) and filled teeth (FT= 2.95) was close to Turks in the same age group. - Comparisons with Third German Oral Health Study: (DMS III, 1999), in which the DMFT value for 12-year-old Germans was calculated as 1.4, indicated a decrease in caries level. The adult Turkish group of 35-44 years exhibited lower DMFT values compared to the DMS III population (1 1.2 vs. 16.9). |
| (2003) Kühnisch, J, et al [31]  *Comparative Study on the Dental Health of German and Immigrant 8- to 10-Years Olds in the Westphalian Ennepe-Ruhr District* | Clinically examined dental caries   - Number of teeth with initial caries - Number of teeth with fissure sealing | DMFT recorded using the diagnostic criteria set by WHO | - Dental caries: Caries prevalence in migrant children (1997: 0.9 DMFS/1999: 1.5 DMFS) was significantly higher compared to the German children (1997: 0.5 DMFS/1999: 0.8 DMFS), p<0.05. Migrants had significantly more initial caries (0.7 1997, 1.3 1999 in German children, 1.3 1997, 2.1 1999 in migrant children) - Fissure sealant: Migrants had less sealed fissures. 1997, the difference was not statistically significant. (0.6 vs. 1.1 in German children). 1999, it was significant (p<0.05) (0.8 vs. 1.7). |
| (2004) Van Steenkiste M,et al [33]  *Prevalence of Caries, Fissure Sealants and Filling Materials among German Children and Children of Migrants* | Clinically examined dental caries   - Number of teeth with fissure sealing - Filling material | dmft and DMFT-Index using the diagnostic criteria set by WHO  Remains of fissure sealants were also recorded as fissure sealant | - Dental caries (6-7 year old): dmft of 6 and 7 year-olds was 1.50 among Germans, 4.61 among German migrants from Russia, 4.02 among Turks, 4.05 among children from former Yugoslavia, 2.35 among Italians, 1.95 among Greeks and 3.76 among children of other nationalities. - Dental caries (9-10 year old): DMFT of 9 to 10 year-olds was 0.31 for Germans, German migrants from Russia 0.77, Turks 1.19, children from former Yugoslavia 1.32, Italians 0.64, Greeks 0.69 and children of other nationalities 0.57 - Caries free: In both age groups the proportion of caries-free children was highest among Germans and lowest among Turkish children - Fillings: The proportion of amalgam fillings to the total number of fillings was higher among 9 and 10 year-olds of Turkish, Italian or Yugoslavian origin than among other nationalities. More Germans had at least one fissure sealant (70.2%) than all other migrant groups (68.5-50.9% depending on the group) |
| (2007) Heinrich-Weltzien R, et al [35]  *Dental health in German and Turkish school children--a 10-year comparison* | Clinically examined dental caries   - DMFT index | DMFT recorded using the diagnostic criteria set by WHO | - Caries free: No significant increase in caries-free Turkish students in both age groups (1.2% increase). However, greatest increase in caries-free dentitions in 12- and 15-year old Germans attending grammar schools with 41.5% and 27% and secondary modern schools with 22.3% and 17%, respectively. - Dental caries: DMFT decrease for all German groups. This was significant for 12-year old Germans at grammar school (decline of 1.5 DMFT) and in 15-year olds at secondary modern schools (decline 2.5 DMFT), and at Grammar schools (decline 2.2 DMFT). P<0.05 - Missing teeth: Turkish students had most missing teeth M(T) at all times in both age groups - Strong polarization of dental caries was found in 2003(no separate analysis for Turkish vs. German students) |
| (2007) Bissar AR, et al [36]  *Dental health, received care, and treatment needs in 11- to 13-year-old children with immigrant background in Heidelberg, Germany* | Clinically examined dental caries and orthodontic problems   - Dental caries - Fissure sealants - Orthodontic treatment | DMFT recorded using the diagnostic criteria set by WHO    Fissure sealants were recorded regardless of whether they were complete or not.  Current or previous  orthodontic treatments were recorded regardless  of being removable or fixed appliances | - Caries free: In all age categories, the group of host children showed a remarkable higher proportion of individuals with a caries-free permanent dentition (DMFT = 0) than the migrants (65.5% vs 45.6%) - Dental caries: Mean DMFT values were significantly different between the two groups for all age categories combined (P< 0.01). In migrant children, 3.3% of the children had one or more missing teeth compared to host population (1.1%) . - Significant caries index: In each age category the values of the Significant Caries Index were higher in the migrant children compared to host population. - Restoration: The obtained unmet values indicated that restorative treatment needs were significantly higher (P< 0.001) among the migrant children than host population. - Sealants: The mean value of fissure sealants per child in migrants was significantly lower than the respective mean value in host population (P< 0.001). - Orthodontic needs: The proportion of migrant children who were provided with an orthodontic treatment (completed or not) was significantly lower than host population children: 31.7% vs. 48.3% (P < 0.001). |
| (2018) Aarabi G, et al [39]  *Oral health and access to dental care – a comparison of elderly migrants and non-migrants in Germany.*  (Findings on questionnaire in Table-3) | Clinically examined oral health   - Dental caries - Oral hygiene - Restoration/ filled component - Bleeding gums | Dmft index  Approximal plaque index (API)  The restoration index: calculated as % of filled teeth of the total number of filled and decayed teeth respectively. The lower the restoration index, the more teeth with treatment need were present.  Bleeding gums- Papillary Bleeding Index (PBI) was assessed to indicate a chronic inflammatory condition. | - Dental caries: DMFT and the number of missing teeth (M) were similar in migrants and host population. In contrast, migrants had on average at least three decayed teeth more and nearly four filled teeth less than host population. - Restoration: Restoration index was much lower in migrants compared to host population (44.0% vs. 79.4%, p < 0.001). - Oral hygiene: Oral hygiene was poorer in the migrants than host population indicated by significantly higher API and PBI values (API: 55.3% vs. 33.0%, p = 0.002; PBI: 46.3% vs. 30.5%, p = 0.016). About two thirds of the migrants and only one third of the host population had a PBI over 40%. - Predictors: The linear regression analyses confirmed that the migration background remained a predictor for having significantly more decayed teeth when controlled for further other predictors. - In the unadjusted logistic regression migration background was a significant predictor for poor oral hygiene; it was almost four times more likely (OR: 3.61) to find insufficient oral hygiene among migrants than host population. But this association decreases when adjusted with other co-variables such as age and sex. |
| (2000) Hjern A, et al [40]  *Dental Health and Access to Dental Care for Ethnic Minorities in Sweden.*  (Findings on dental visits in Table-3) | Self-reported oral health (interview based)   - Use of Prosthesis - Chewing problems - Any caries in teeth   *Questionnaire was not validated* | - Based on annual survey of living condition. | - Prosthesis: In the adult study population 15% of the Polish-born, 12% of the Chilean-born and the Turkish-born study group, and 6% of the Iranian-born study group, had a complete or partial prosthesis, compared with only 3% of the host population. - Chewing problems: Between 7% and 16% of the migrants reported chewing problems, compared with only 3% in the host population. - Dental caries in children: 33–42% of the migrant children were reported to ever have had caries, compared with 24% in the host population - Overall, migrants have poor reported oral health and hence a greater need for treatment compared to host population. |
| (2001) Hjern A, et al [41]  *Social inequality in oral health and use of dental care in Sweden.*  (Findings on dental visits in Table-3) | Self-reported oral health (interview)   - Use of Prosthesis - Chewing problems - Any caries in teeth   *Part of the study reported by Hjern et al in 2000* | - Based on annual survey of living condition.   *Same as above* | - Prosthesis: The odds of wearing a prosthesis were higher among migrants compared to host population (OR 1.8, CI-95% (1.5–2.2). and odds of - Problems with chewing: Also higher among migrants compared to host population; OR 2.1, CI-95% (1.6–2.8).compared to the rest of the population. - Dental caries: The odds of ever having had caries were higher in children with migrant parents compared to host population; OR 1.4 CI-95% (1.1–1.7). |
| (2004) Stecksén-Blicks C, et al [42]  *Caries Experience and Background Factors in 4-Year-Old Children: Time Trends 1967–2002*  (Findings from questionnaire in Table-3) | Clinically examined dental caries   - dmft index | Decayed, missed, and filled surfaces using a mirror and a probe were recorded with the same methods and criteria as in the earlier studies according to definitions described by Koch. | - 9% of the children were migrants and they had a significantly higher dmfs value than host population, 6.8 ± 5.5 and 1.5 ± 2.9, respectively (p < 0.001). - Note: dental caries decline began in 1967 and continued for 20 years and then levelled out. This may be due to tooth brushing with fluorides as a cries preventive program in children. |
| (2005) Jacobsson B , et al [43]  *Dental caries and caries associated factors in Swedish 15-year-olds in relation to immigrant background.*  (Findings from questionnaire in Table-3) | Clinically examined dental caries   - dmft index | Number of teeth extracted because of caries.  Prevalence of enamel caries on proximal surfaces (D_e_S _Proximal_)  Prevalence of dentine caries on proximal surfaces(D_d_S _proximal_)  Prevalence of filled proximal  Surfaces (FS _Proximal)_. | - Dental caries status: The proportions of adolescents free from dentine and enamel carious lesions on the proximal surfaces were similar among migrants and host population (24% and 26%, respectively) as were the proportions with enamel or dentine carious lesions or fillings. - However, migrants had significantly more proximal surfaces affected by enamel or dentine caries lesions than host population did (6.5 and 4.0 D_ed_ FS _Proximal_ respectively; p = 0.02), reflecting more surfaces with enamel carious lesions (D_e_ S _Proximal_) in the former group (5.5 compared with 3.3; p =0.02). - Age at migration: Migrant adolescents born in Sweden or arriving before 1 year of age (n=23) had a mean number of proximal carious lesions (D_ed_ Pr_oximal_) that was similar to that of host population (adolescents) [5 .0 (3.2-9.0) compared to 4.0 (2.9 - 5.3), NS]. In children arriving after 1 year of age, but before 7 years of age, (n=2o) the number of affected proximal surfaces increased to 6.4 (2.7- 8.2). If the child was more than 7 years old on arrival in Sweden (n=8), mean caries prevalence (D_ed_ FS_Proximal_) increased further to 10.8 (4.0 - 13.1). The ANOVA among the groups yielded a p-value of o.o6. Higher dental caries prevalence in newly arrived migrant adolescents is mainly at initial level (caries not advanced to dentine), similar to host population. |
| (2008) Stecksén-Blicks C,et al [44]  *Caries prevalence and background factors in Swedish 4-year-old children – a 40-year perspective.*  (Findings from questionnaire in Table-3) | Clinically examined dental caries   - dmft index | Decayed, missed, and filled surfaces using a mirror and a probe were recorded with the same methods and criteria as in the earlier studies according to definitions described by Koch. | - 16% of children were classified as migrant children. - 59% of migrant children had caries compared to 32% in the rest of the 2007cohort (P< 0.01).   *Part of series of cross sectional studies: (2004) Stecksén-Blicks C, et al.* |
| (2010) Julihn A, et al [45]  *Migration background: a risk factor for caries development during adolescence* | Secondary data on dental caries   - DMFT index | From the Total Population Register kept by the SCB | - Approximal caries: Of the cohort (n = 15,538), 38.7% (n = 6,006) were found to have approximal caries increment. Of these, 5,310 were born in Sweden, 63 in western Europe, 175 in eastern Europe, 305 in Asia, 81 in Africa, and 72 in South America, yielding an incidence proportion of 37.5, 45.0, 55.6, 51.3, 56.6, and 38.9%, respectively (highest mean approximal caries increment was found among children born in eastern Europe, Africa and Asia). - The bivariate logistic-regression analysis of approximal caries increment between 13 and 19 years of age, as a dependent variable, showed that most of the independent variables were statistically significantly associated with the outcome in the analysis, with the highest ORs seen for the following variables: e.g. - both parents born in Africa (OR 2.47; 95% CI = 1.93–3.17), both parents born in eastern Europe (OR 2.39; 95% CI = 2.06–2.78), etc. - Among children born abroad, the following categories exhibited a statistically significant increased risk for approximal caries increment were- mother born in Sweden/ father born abroad (OR 2.06; 95% CI = 1.05–4.07), father born in Sweden/mother born abroad (OR 1.54; 95% CI = 1.16–2.03), and both parents born abroad (OR 1.73; 95% CI = 1.26–2.38) - Parental migration should be considered as a risk factor for caries development during adolescence, irrespective of whether or not the adolescent was born in Sweden. - Summary: Migrant children exhibited a higher risk of increase of approximal caries increment during adolescence compared to host population, irrespective of whether or not the children were born in Sweden, especially among Eastern European children. |
| (2010) Mousavi SM, et al [46]  *Nasopharyngeal and hypopharyngeal carcinoma risk*  *among immigrants in Sweden* | Secondary data from records   - Nasopharyngeal carcinoma - Hypo pharyngeal carcinoma | The standardized incidence ratios (SIRs) were calculated as the ratio of observed to expected number of cases. | - Nasopharyngeal cancer: As compared to the native Swedish population, the risk of nasopharyngeal carcinoma was significantly higher in male (SIR = 35.6) and female (24.6) Southeast Asians, male (12.4) and female (34.7) North Africans, male (4.9) and female (10.9) Asian Arabs and other male Asians (6.2 to 6.7) .The incidence of nasopharyngeal carcinoma is higher in Southern China, Southeast Asia, North Africa and some part of Middle East than in other regions of the world. - Among migrants from the European countries, only the men from former Yugoslavian showed an increased risk (2.7). - Hypopharyngeal cancer: Only male migrants from the Indian Subcontinent had an increased risk (5.4) of hypopharyngeal carcinoma as compared to native Swedes. |
| (2014) Stecksén-Blicks C, et al [47]  *Caries and background factors in Swedish 4-year-old children with special reference to immigrant status.*  (Findings from questionnaire in Table-3) | Clinically examined dental caries   - dmft index | Decayed, missing and filled surfaces (dmfs) were recorded using the same methods and criteria as in the earlier studies according to definitions described by Koch. | - Dental caries trend over time: The proportion of children with caries in 2012 (22%) was statistically significantly lower (p < 0.05) than in 2007 (38%). Overall, the proportion of children with caries gradually decreased among host population between 2002–2007 and between 2007– 2012 (p < 0.01). A similar decrease was noted for immigrant children between 2002–2007 (p < 0.01), but not between 2007–2012 (p > 0.05). - Dental caries in migrants**:** More migrant children had caries (p < 0.01) than host population (58% vs 15%). The distribution of dmfs scores also had a statistically significant difference (p < 0.01) between migrant and host children.   *The caries prevalence had an obvious decline between 2007–2012, but this decline was limited to non-immigrant children*  *Part of series of cross sectional studies: (2004 )and (2008) Stecksén-Blicks C, et al.* |
| (2016) Olerud E, et al [48]  *Oral health status in older immigrants*  *in a medium-sized Swedish city.*  (Findings on questionnaire in Table-3) | Clinically examined oral health   - Dental caries - Number of teeth - Denture wearers - Plaques - Dry mouth - Periodontal health | DMFT in accordance with criteria described by Koch and Baume.  The number of teeth with a natural root was registered.  Full and partial dentures including implants  Plaque was registered  using one part of the index MPS (mucosal plaque score)  Salivation was measured using a simplified method. | - Number of teeth: The prevalence of edentulous subjects was high, approximately 17% of the elderly migrants had no natural teeth. The mean number of teeth among those with natural teeth was 22.3, and men had more teeth than women ( *p* <0.05). - Dentures: More common for women to have removable dentures compared with men (p <0.05). - Dental caries: The experience of caries in the group of elderly migrants was the same in men and women. The DFT index (decayed and filled teeth) was in median 5.5 to 6 and 75% of those with natural teeth had at least one tooth with a caries lesion. - Periodontal health: One quarter of the individuals had teeth with gingival pockets of 6 mm or deeper while 63% showed bleeding from the gingiva when probing. - Overall, majority had caries lesions, but fewer showed periodontal diseases. |
| (2004) Ferro R,et al [49]  *Comparison of data on Early Childhood Caries (ECC) with previous data for Baby Bottle Tooth Decay (BBTD) in an Italian kindergarten population.* | Clinically examined dental caries (Early childhood caries)   - dmft index | Caries was scored using WHO [1997] | Both ECC and S-ECC (severe form) were significantly higher in the immigrant group than in the native born children (p>0.001%). Prevalence of ECC is around 3 times more than native born and S-ECC is 6 times more frequent. |
| (2007) Ferro R, et al [50]  *Preschoolers’ dental caries experience and its trend over 20 years in a North-East Italian Health district.* | Clinically examined dental caries   - dmft index. | Dmft index for diagnose of dental caries occurrence at dentinal level. | - Dental caries: Migrants had statistically lower caries free population – 46% (n=89/192) compared to host population 77% (n=1801/2332) (p<0.000). Mean dmft was higher among migrants in 3, 4 and 5 year old, although not statistically significant. However, dmft percentage showed that among 3 year old, % dmft>0 were 34% compared to 15% among host children (p= 0.001). - In 5 years old children the prevalence decreased from 75% in 1984 to 32 % in 2004. - Ethnicity is a good predictor of oral health status (tooth decay: OR 3.9, CI 95% 2.91-5.35). |
| (2007) Ferro R,et al [51]  *Oral health inequalities in preschool children in North-Eastern Italy as reflected by caries prevalence.* | Clinically examined dental caries   - dmft index. | Diagnosis of dental caries at the tooth level (dmft index), according to the criteria recommended by the British Association for the Study of Community Dentistry (BASCD) | - Caries free: Migrants had statistically lower caries free population: 50% (n=138/277) compared to host population, with 78% (n=2437/3124) (p<0.000) - Dental caries: The overall prevalence of dentinal caries was: 15.4% in 3-year-olds, 24.2% in 4-year-olds and 31.1% in 5-year-olds. At the same ages, migrant children showed a significantly higher prevalence of caries compared with their counterparts (31.1% in 3 year old, 47.7% in 4 year old and 63.8% in 5 year old). - Predictors: Age and ethnicity are good predictors of oral health status: 4 year old and 5 year old children vs 3 year old showed an increased risk of caries occurrence (OR 1.7, IC 95% 1.35-2.11, and OR 2.5, IC 95% 2.01-3.10, respectively) as well as in migrants (OR 3.6, IC 95% 2.76-4.60). |
| (2007) Ferro R, et al [52]  *Prevalence and severity of dental caries in 5- and 12-year old children in the Veneto Region (Italy).* | Clinically examined dental caries   - dmft/ DMFT index. | Dental caries was diagnosed at the caries into dentine (D_3_) threshold using a visual method.  Care index percentages were also determined (ft/dmft x 100 for 5-year-olds, FT/DMFT x 100 for 12-year-olds). | - 5 year old: Migrant children were very low in this group (n=16) and hence no discussion was possible and were excluded. - 12 year old: The mean DMFT for (n=48) migrants (3.23 ± 1.17) was significantly higher (p<0.00 1) than that of 814 host children (1.33 ± 1.86); Care index for migrant children was notably lower (36.2% vs. 60.9%). - Also in 12 year old, the percentage of migrants with DMFT=0 was about one-third that for Italians (27.0% vs. 56.8%), migrants had four times as many decayed teeth (D_3_T, 2.04 vs. 0.52), only 10.4% had at least one sealed tooth (vs. 40.9%). |
| (2005) Skeie MS, et al [54]  *Caries patterns in an urban preschool population in Norway* | Clinically examined dental caries   - Carious exam: 5-point scale, dmft (d_1_-d_5_). | 5-graded caries diagnosis based on paper by Amarante et al, 1998  Dental caries was measured on d_1-5_ scale with radiographs (BW). | - 67% of carious surfaces were found in 10.7% of the children; the migrant group made up 42.2% of this high caries experience group. - The d-component (both on surface and tooth level) was significantly higher at both ages in the migrant groups compared to the host population (p<0.001) - The proportions of caries free 3 and 5 year old children in the host population were 84.1% and 52.5%, and 50.0% and 11.4% in the migrant groups respectively. - The frequencies of restorations and/or manifest lesions were 34.1% and 70.5% in the 3 and 5 year old migrant children. - Teeth extracted due to caries (n=12) were localized to eight in 5 year old children, two of them being migrants. |
| (2006) Skeie MS,et al [55]  *Parental risk attitudes and caries-related behaviors among immigrant and western native children in Oslo.*  (Findings on questionnaire in Table-3) | Clinically examined dental caries   - Carious exam:5-point scale, dmft (d_1_-d_5_). | Clinical exam- By dental hygienist with radiographs (BW). | - Dental caries was reported to be high in migrant group as compared to the host population in all the teeth and in 3 and 5 year old age groups. (p<0.001). No difference was observed within the migrant group (Muslim vs non-Muslims) - Caries experience: In bivariate analysis, it was observed that 3 year old migrant children were three times more likely to have caries experience (RR=3.0) and 5 year old migrant children nearly twice as likely compared to host population (RR=1.9). - The caries experience of the children of mothers who had lived in Norway for more than 5 years (n =51, mean age = 4.0 years) was 2.3 times as high as that of children of more recent arrivals (n = 14, mean age = 3.5 years) (p=0.008). - It was also seen that caries experience (d1-5mfs) was inversely related to the parents’ educational level for 3 year olds (r =- 0.20 for mothers and r=-0.20 for fathers). Among the 5 year old children, these correlations were r =- 0.25 for mothers and r =- 0.19 for fathers. |
| (2008) Skeie MS, et al [56]  *Caries increment in children aged 3–5 years in relation to parents’ dental attitudes: Oslo, Norway 2002 to 2004.*  (Findings on questionnaire in Table-3) | Clinically examined dental caries   - Carious exam: 5-point scale, dmft (d_1_-d_5_). | Same criteria used as seen in Skeie et al 2006 | - Comparison between 2002-2004: The caries prevalence of the 3-year olds who did not continue in the study, did not differ significantly at baseline from that of the study group (16.3% and 19.7% at d1-5mfs level, respectively, P = 0.574). - There were 13 migrant children (41.9% of the migrant group) with three or more new caries lesions at the d_3_-_5_mfs level. |
| (2010) Wigen TI, et al [58]  *Caries and background factors in Norwegian and immigrant 5-year-old children.*  (Findings on questionnaire in Table-3) | Clinically examined dental caries   - dmft index. | Surfaces were given a code according to status: sound (s), decayed (d), filled (f) or missing due to caries (m)-  Five caries graduations were recorded (d_1_–d_5_) | **Results for dental caries not discussed for migrant population-**   - Amongst migrants, the probability of child having dental caries was 9 times higher and probability of child having dentinal caries was 12 times higher, if both the parents had low education |
| (2006) Almerich Silla JM, et al [59]  *Oral health survey of the child population in the Valencia Region of Spain (2004)* | Clinically examined dental caries   - dmft/DMFT index - Sic Index - Periodontal health | Dental caries (dmft/ DMFT) hrough WHO criteria.  The Community Periodontal Index (CPI) was employed, using 3 scores (healthy, bleeding after probing and calculus detected) and examining only the 12 and 15-16 year-old children. | - Dental caries: Migrant children had DMFT as 2.43 at 12 years and 4.23 at 15-16 years, while their dft at 6 years was 3.15. Amongst the Spanish children, their DMFT was 1.07 at 12 years, 1.84 at 15-16 years, while dft at 6 years was 1.08 (p <0.005). This shows a far higher dental caries among migrants compared to the Spanish children. - Periodontal health was not compared between the two groups. |
| (2007) Almerich-Silla JM, et al [60]  *Influence of immigration and other factors on caries in 12- and 15-yr-old children.*  (Findings of oral health behaviours in Table- 3) | Clinically examined dental caries   - DMFT and SiC index | WHO criteria followed. 3 outcome variables were considered: the DMFT count, the prevalence of DMFT > 0, and the significant caries index (SiC) as defined by Brathall.  The SiC is calculated as the average DMFT for the one-third of the sample with the highest DMFT counts and provides a clear reflection of the situation within the group of individuals who have the highest caries experience. | - 12 year old: The DMFT count and caries prevalence of 12-yr-old migrant children was significantly higher (p = 0.009 and p = 0.001, respectively) than in 12-yr-old Spanish children. - 15 year old: The same significant differences (p = 0.001 and p = 0.025) were obtained in children of the 15-yr-old age-group. - Caries free: Only 28% of the 12-yrold and 23% of the 15-yr-old migrants were caries free (DMFT = 0), compared with 59% of the 12-yr-old and 46% of the 15-yr-old Spanish children - The SiC index was noticeably higher among the migrants than among the Spanish children, and the difference was highly significant at age 15 year. - Migrant status, intake of cariogenic foods, social class, and age were significantly associated with caries presence, and migrants were 2.8 times more likely to have caries than were Spanish children. |
| (2018) Muñoz‑Pino N, et al [61]  *Comparing Oral Health Services Use in the Spanish and Immigrant Working Population.*  (Findings of oral health service use in Table- 3) | Self-reported oral health (Questionnaire based)   - Self-reported dental decay - Tooth loss and gingival bleeding   *Questionnaire was validated* | Self- reported Oral health status was assessed. | - A greater proportion of self-reported dental caries was observed in migrant women (28%) than in Spanish women (23.9%). - The prevalence of extractions (lost teeth) was greater in the Spanish population (men: 69.9% and women: 69.4%) than in migrants (men: 63.8% and women: 55.9%). - The prevalence of gingival bleeding was greater in Spanish women (19.2%) than in migrant women (13.7%). |
| (2019) Valcarcel Soria R, et al [62]  *Acculturation and Dental Caries Among Children in Spain.*  (Findings of acculturation in Table- 3) | Secondary data (Clinically examined dental caries)   - Dmft index | Secondary data from the clinical records on dental caries status. | - Caries experience was the highest among first generation children (dft + DMFT = 8.2), those who arrived in Spain before 6 years of age (9.1), those with 10 or more years living in Spain (9.6), and those speaking other language than Spanish at home (8.8). - Generational status was significantly associated with children’s caries experience. First and second generation migrant children had, respectively, 15% (95% CI 3–28%) and 19% (9–31%) greater caries experience than Spanish-born children. These differences remained significant after adjusting for child factors (sex and age) in Model. - When the first and second generation migrant children were combined in a single groups, they had 13 % (4-24%) greater caries experience than Spanish born host children. |
| (2016) van der Tas JT, et al [64]  *Ethnic Disparities in Dental Caries among Six-Year-Old Children in the Netherlands* | Clinically examined dental caries   - dmft =0 - dmft= 1-3 - dmft >3 | The dmft index was obtained from intra-oral photographs.  On average, all teeth were captured on in total 10 photographs showing the occlusal, buccal, lingual and palatal sides of the teeth. | - Dental caries: Host population had the highest percentage of children without caries (77.1%). By contrast, in the group of Turkish (n = 402) and Moroccan (n = 308) children, only 41.5 and 40.9%, respectively, had a caries-free dentition. - Odds of dental caries: Children with a Surinamese-Hindustani, Surinamese-Creole, Turkish, Moroccan and Cape Verdean background had significantly higher odds of having dental caries than Dutch children. Turkish and Moroccan children had significantly higher odds of having mild caries (dmft 1–3: OR, 2.73; 95% CI, 2.09–3.57; OR, 2.85; 95% CI, 2.12–3.83, respectively) and even higher odds of having severe dental caries (dmft >3: OR, 8.90; 95% CI, 6.76–11.7; OR, 8.43; 95% CI, 6.21–11.4, respectively) than Dutch children. - Ethnicity and dental caries: The associations between Surinamese-Hindustani (OR, 2.36; 95% CI, 1.49–3.73), Turkish (OR, 5.12; 95% CI, 3.73–7.04), or Moroccan background and severe caries (OR, 5.12; 95% CI, 3.54–7.41) remained significant after correcting for SES factors. - Demographics and dental caries: Educational level of the mother and household income, indicators for the SES, were significantly higher in the Dutch group (n = 2,957) than in the migrants. |
| (2003) Sundby A, et al [65]  *Oral health status in relation to ethnicity of children in the*  *Municipality of Copenhagen, Denmark.*  (Findings from the questionnaire in Table-3) | Clinically examined dental caries   - dmft /DMFT index. - Severity of dental caries recorded zone wise. | Zone1- no tooth surfaces have caries experience; zone 2- caries experience in fissures or pits in molars and/or premolars;  Zone 3- approximal caries in canines and/or molars and/or premolars;  Zone 4- caries experience in incisors and/or smooth surfaces | - At age 3: Children of ethnic backgrounds had significantly higher dental caries experience (p<0.05), especially Pakistani, Albanian and Somalian children compared to host population. - At age 5: The same was true among children 5 years of age, with Turkish and Albanian children having significantly higher caries experience (p<0.05). - At age 7: Except for the Somali children, children of foreign nationalities had significantly higher prevalence as well as mean caries experience than did Danish children (p<0.01) - At age 15: Only Albanian teenagers had significantly higher caries experience (p<0.001) whereas other nationality teenagers had caries experience almost similar to host population. - Severity: Children of ethnic minority groups more often had severe dental caries than had Danish children of corresponding ages, statistically significant for Albania. At age 15, statistically higher severity of dental caries was seen among Turkish and Albanian adolescents (p<0.05). |
| (2010) Christensen LB, et al [66]  *Oral health in children and adolescents with different socio-cultural and socio-economic backgrounds* | Secondary data (Clinically examined dental Caries)   - Dmft index | Caries occurrence was recorded at the cavity level.    Caries prevalence was expressed as a percentage and caries experience was quantified by means of the dmfs/ DMFS index. | - Caries prevalence was found to be 30%, 51%, 57% and 63% among the 5-, 7-, 12- and 15-year-olds, respectively. In all age groups, the percentage of children with caries experience was higher among children with foreign ethnic backgrounds. Among 5- and 7-year-old children with non-Danish mothers, mean caries experience was three to four times higher than among children of Danish mothers. - Migration status of the mother: The odds ratio for children with caries was higher for mothers with migrant status than for descendant mothers (born in Denmark) (p < 0.001). - Multivariate logistic regression model for caries experience revealed that few children in the family, a high family income, a highly educated mother or a Danish mother still seemed to have a positive influence on children’s caries status (p < 0.001). |
| (2011) Gatou T, et al [67]  *Dental caries prevalence and treatment needs of 5- to 12-year-old children in relation to area-based income and immigrant background in Greece.* | Clinically examined oral health   - Dental caries - Oral Hygiene Index | DMFT recorded using the diagnostic criteria set by WHO.  The simplified Debris Index (DI-s) was also recorded. | - Primary teeth: Migrant background was significantly negatively correlated with all the assessed oral health outcomes (P < 0.001) with migrants caries experience in primary teeth being 75.2% compared to Greek children (46.5%). - Permanent teeth: Similarly in the permanent teeth, the caries experience among migrant children was statistically significantly high (45.7%) compared to Greek children (26.5%). - Higher age, migrant background, and living in lower income areas was consistently associated with higher odds of caries prevalence, both in the deciduous and permanent dentitions with odds ratios ranging from 2.65 to 4.40 for the ethnic background. - Overall, children with an migrant background have 1.68 to 4.34 higher likelihood to present higher dmft and DMFT values, higher unmet treatment needs and poorer oral hygiene levels compared to their Greek counterparts. |
| (2017) Mantonanaki. M, et al [68]  *Socio-demographic and area-related factors associated with*  *the prevalence of caries among preschool children in Greece* | Clinically examined oral health   - Dental caries - Oral hygiene levels - Dental caries experience - Treatment needs (UTN index) | Dental caries was diagnosed at the cavitation level (d_3_) threshold using a visual method.  Oral hygiene level was recorded first using the Simplified Debris Index (DI-s, Greene and Vermillion, 1964).  Dental caries experience was assessed using the WHO criteria and summarized as decayed, missing and filled teeth (dmft index). | - Dental caries: More than half of the non-Greek and over a third of the deprived children were affected with dental caries. Caries prevalence was higher in non-Greeks, deprived children (striving and blue collars categories) and boys by 34.4 % and 23.9%, 3.7% in comparison to Greeks, affluent children and girls. Only migrant status was significantly related to higher dmft scores (IRR=1.36, p<0,05). Overall, migrant children had a 31% lower probability of no detectable caries experience than Greeks. - Migrant status was strongly associated with dmft score with the mean predicted probability for non-Greek children having 1-2 carious teeth was 16% (95% CI12%, 19%) and 13% (95% CI8%, 17%) respectively. - Non-Greek children were 10% (95% CI7%, 14%) more likely to have one carious tooth, than Greek children, while the difference in the probability for two carious teeth was 12% (95% CI7%, 15%). |
| (2014) Cvikl B, et al [69]  *Migration background is associated with caries in Viennese school children, even if parents have received a higher education.*  (Findings of oral health questionnaire in Table- 3) | Clinically examined dental caries   - DMFT index - SiC index | DMFT and DMFS indices (WHO 1997 criteria) were used.  The SiC Index (Significant  Caries Index), the mean DMFT of the one-third of the group with the highest DMFT scores, and the necessity of treatment due to dental decay (DT score) were subsequently calculated using those values. | - Dental caries: The mean DMFT index was 2.33 and 1.50 (p < 0.001), the mean DMFS index was 3.51 and 2.15 (p < 0.001), the mean SiC index was 4.93 and 3.54 (p < 0.001), respectively among children with a migration background and children without migration background. - Treatment needs: The need for treatment was 2.4 times higher in children with a migration background compared to children without migration background, demonstrated by the mean DT of 0.83 and 0.34, respectively (p < 0.001). - Tooth loss as a result of caries was observed 3.5 times more frequently in children with a migration background compared to children without migration background as shown by the reflecting MT values of 0.07 and 0.02, respectively (p = 0.010). |
