# Supplementary material for: Oral Health Status, Oral Health Behaviours and Oral Health Care Utilisation Among Migrants Residing in Europe: A Systematic Review
Source: J Immigr Minor Health. 2020 Jul 19;23(2):373–88. doi: 10.1007/s10903-020-01056-9 (PMC7914188; doi:10.1007/s10903-020-01056-9)
Supplement: Supplementary file 4 — Supplementary file4 (DOCX 61 kb) [file 10903_2020_1056_MOESM4_ESM.docx]

**Appendix Table 4: Details of oral health behaviours and utilization**

| **Author/ Title of the article** | **Variables Measured And**  **Methodology Used**  **(Measuring Criteria Used)** | **Observed Findings**  **(As reported)** |
| --- | --- | --- |
| (2000) Robinson PG, et al [1]  *Dental caries and treatment experience of adults from minority ethnic communities living in the South Thames Region, UK.* | Predictors of DC were measured in this study (Combination of self-completed questionnaire and interview assistance)   - Demographics - Tooth brushing behaviours - Number of sugar exposures - Tobacco/ Alcohol consumption - Duration of stay in UK   *Piloting of the questionnaire mentioned but its validity not mentioned* | - The most consistent predictors of the presence of caries or dental treatment experience (i.e. DMFT ≥1.0) showed that age (1.56;OR 1.30-1.88), visit UK dentist (1.59;OR 1.21-2.10) and duration of stay in UK (1.03;OR 1.01-1.06) were statistically significant for the whole sample*.* - Two other variables were significant in the model for the whole sample: seeking advice about dental problems (OR: 1.49 and 95% CI: 1.17-1.90) and drinking alcohol (OR:1.31 and 95% CI:1.02-1.67). - Dental attendance: 90% of the sample claimed to visit the dentist at least once a year compared to reported attendance of South England being 47%. |
| (2001) Pau AKH, et al [4]  *Self-reported oral health status and oral-health related behaviours of a sample of Chinese elders in Inner London, UK: A pilot investigation* | Interview   - Socio-demographic characteristics - Use of dental services and barriers to use - Oral health behaviours - Knowledge and traditional health beliefs.   *Piloting of the questionnaire and its validity mentioned* | - Dental attendance pattern: 17 subjects (31%) had visited a dentist in the previous 6 months. A total of 49 subjects (91 %) had experienced language difficulties in their use of health services with 36 subjects (67%) having language difficulties all the time. - Reason for dental visit: Of 32 subjects who had visited a dentist in the previous 3 years the main reasons were toothache (13 or 24%), problems with dentures (12 or 22%) and routine examination (7 or 13%). - Dental knowledge: 44% (n=24) thought that poor oral hygiene could cause tooth decay and 41% (n=22) said that tooth brushing can prevent dental caries. - Oral hygiene practices: 74% (N=40) brushed their teeth twice daily and most common cause for tooth decay was reported as sweets by 56% (n=30) and gum diseases happened most commonly due to traditional practice of ‘HOT AIR’ as reported by 51% (n=31). - Summary: Self-perceived overall health was found to be associated with self-perceived condition of teeth. Migration and ethnicity may have an impact on dental care utilisation. |
| (2008) Hullah E, et al [10]  *Self-reported oral hygiene habits, dental attendance and attitudes to dentistry during pregnancy in a sample of immigrant women in North London* | Interviews   - Past dental attendance - Reasons for dental attendance - Maternal age - Parity and socio-economic group   *Piloting of the questionnaire or its validity: not mentioned* | - General finding: 74.2% of the mothers were not born in the UK and 38.3% were Black African. - Dental attendance: 34% of women questioned reported regular attendance for dental care with the mothers from socioeconomic groups 4 and 5 being more likely to be non-frequent attenders. The average time since their last visit to a dentist was 21.1± 4.2 months. Of the sample group, 34% had never experienced dental pain and 13% were smokers. - Oral hygiene practices: The majority reported good oral hygiene habits such as brushing their teeth more than once daily (74%) and using mouthwash (51%). - Awareness on available dental services: When questioned if they knew of the availability of free dental care in pregnancy and for 12 months after delivery, 26% admitted that they were unaware of such a concession, although 15% of this group had been pregnant previously in the UK and yet were still unaware of the provision of free dental care. - Dental problems: 44% of women reported gum problems during pregnancy such as bleeding gums when brushing their teeth and 36% felt that the condition of their teeth had deteriorated during pregnancy. - Summary: Pregnancy did little to change their attitudes to dental care. There appears no difference in attitudes to dental care between migrant and British born pregnant women. |
| (2011) Reekie T. [11]  *The effect of South Asian ethnicity on satisfaction with primary cleft lip and or palate repair.* | Secondary data (Questionnaire)  Satisfaction with Appearance questionnaire (SWA), developed especially for cleft patients by the Psychology Special Interest Group of the Craniofacial Society of GB and Ireland, to assesses satisfaction with overall appearance  *Questionnaire was validated* | - Satisfaction with cleft surgery: South Asians were found to be less satisfied with many aspects of their cleft surgery. Satisfaction with nose and speech were found to be significantly less in the South Asian group compared with the host population (Caucasian group) (mean difference >20, p < 0.01). - When age was adjusted, overall trends were seen as- age increases, the satisfaction score decreases (nose= -0.625 and speech= -0.797). - Multiple linear regression showed that ethnicity was still significant when adjusted for age, gender, cleft type and deprivation score. |
| (2013) Al-Haboubi M, et al [13]  *Inequalities in the use of dental services among adults in inner South East London* | Interviews   - Demographics - Use of dental services - Reason for their last dental visit - Type of dental service used - Satisfaction with the care received - Improvements, if any.   *Questionnaire was validated* | - Dental visits: 69% of respondents reported having visited the dentist in the last 2 years, irrespective of their sex, age, ethnicity and social grade. In the fully adjusted model, women and Asian people were 14% and 21% more likely to have visited the dentist in the last 2 years than were men and White people, respectively. Asian people were also 28% more likely to have visited the dentist in the last 2 years than were Black people. - Recommendations: Staff (37%), costs (25%), and opening hours (18%) were the commonest areas where participants made recommendations for improvements to local dental services. Greater availability of NHS dentists (32%), greater affordability of dental care (16%), and greater flexibility in opening hours (11%) were mentioned elaborately. - Satisfaction: Among the 475 participants who visited a dentist in the last 2 years, 90% were satisfied with the quality of care provided during that visit. |
| (2017) Arora G, et al [18]  *Ethnic differences in oral health and use of dental services: cross-sectional study using the 2009 Adult Dental Health Survey.* | Interviews   - Lifestyle - Use of dental services - Type of dental services - Oral hygiene practices | - Sugar consumption: Host population (Whites) were least likely to add sugar to hot drinks (35.4% vs 71/5% in Pakistani/ Bangladeshi: p<0.001) but they consumed sweets and cakes significantly more frequently than migrants (16.9% and 30.3% respectively compared to 3.2 and 8.6% respectively among blacks: p<0.001) - Black participants consumed sweets and cakes least frequently. However, they were the most frequent consumers of fizzy drinks (30.% compared to other ethnic groups: p<0.001). - Use of dental services: Use of private sector dentists was most common among host population (Whites), and least common among the combined Pakistani/Bangladeshi group (28.3% vs 11.5%: p<0.001). - Dental hygiene: Compared with host population (Whites), South Asian participants were less likely to have used dental hygiene products (such as dental floss or mouthwash); - Dental visits: Migrants attended routine dental clinic visits less frequently and were more likely to report that they only attended the dentist if they suffered symptoms (p<0.001) - Smoking: was higher among host population compared to other migrants. |
| (2000) Khan FA,et al [22]  *Predictors of tobacco and alcohol consumption and their relevance to oral cancer control amongst people from minority ethnic communities in the South Thames health region, England* | Questionnaire   - Alcohol use - Tobacco smoking - Chewing tobacco   *Questionnaire was pre-tested and validated* | - Self-reported alcohol and tobacco use: In the Black-Caribbean group (n=162) 68% of respondents reported alcohol use and 18% smoked tobacco. In contrast, only 3% of the Bangladeshi group (n=103) said they drank alcohol. Chewing tobacco was common amongst the Indian, Bangladeshi and the Black-African groups, however no significant relationship was found between chewing and the Black-African group. - Predictors of tobacco smoking: In the Black- Caribbean group younger people were more likely to smoke. - Predictors of alcohol consumption: In the Black-African group, those who had been in a full-time education beyond the age of 16 years were more likely to consume alcohol. In the Indian group alcohol consumption was predicted by being male, employed and being born in the UK. - Predictors for chewing tobacco: In the Indian group, people who were educated beyond the age of 16 years were more likely to chew products containing tobacco. In the Bangladeshi group, where those who were less educated were more likely to chew tobacco products. - Overall, men were more likely than women to smoke tobacco. Chewing of pan and tobacco was common in the South Asian communities and alcohol consumption was high among the Black–Caribbean group. Those factors were predicted by education, employment, gender and being born in the UK. |
| (2000) Vora A.R, et al [23]  *Alcohol, tobacco and paan use and understanding of oral cancer risk among Asian males in Leicester* | Questionnaire   - Alcohol use - Tobacco consumption - Paan chewing - Areca nut use - Paan Masala use - Knowledge of oral cancer risks - Prevention methods   *Piloting of the questionnaire or its validity: not mentioned* | - Alcohol use: Alcohol use was principally found in the Sikh and Hindu communities, with highest alcohol consumption among 1st generation Asians being observed in the Sikh community (p<0.001). The Muslim community was found to have very low alcohol usage, with 92% of 1st generation Muslims and 94% of 2nd generation Muslims claiming not to drink any alcohol. Alcohol use was also low in the Jain community. - Tobacco use: Among 1st generation males, the high use of tobacco observed among Muslim males compared with the other groups was significant (p<0.001). However, the decrease in the number of 2nd generation Muslim males smoking tobacco compared with their elders was significant (p<0.05). - Paan chewing: Highest numbers of regular paan users were found in the 1st generation Muslim community, with 25% of the sample group claiming to chew paan regularly. This is in contrast to the low usage of paan among 1st generation Sikhs, where only 2% were regular paan chewers. The chewing of paan is also prevalent among 2nd generation Hindus, Muslims and Jains, with high numbers of regular users among Muslim and Jain males. - Areca Nut use: Among 1st generation males, highest numbers of areca nut chewers were found among the Jain community, where 28% chewed areca nut regularly. Among the 2nd generation sample, highest use was by Muslim males where 17% regularly chewed areca nut; compared with 13% of Hindu and 12% of Jain males. - Paan Masala use: The use of paan masala among 1st generation males, either on a regular or occasional basis, was noted in 15% of Muslim, 10% of Hindu and 8% of Jain males .Among 2nd generation males, a similar picture of use was observed. - Combined Habit: Among 1st generation males, 9% of Hindu males combined all 3 habits of tobacco, alcohol and chewing paan or areca nut. 27% of Muslim males combined their chewing habit with tobacco smoking. A high proportion of Sikhs (55%) drank alcohol as their only habit. Among the 2nd generation group, a similar picture of combining habits was noticed. - Oral Cancer Awareness- Among 1st generation Asian males, approximately 50% had heard of oral cancer, except for the Sikh group where only 17% of the sample group had heard of the condition (p<0.001). 75% of 2nd generation Jains had heard of oral cancer, compared with 43–48% in the other 2nd generation groups (p<0.1). A greater proportion of 2nd generation Sikhs (48%) had heard of oral cancer compared with their first generation elders (17%) (p<0.01). |
| (2001) Farrand P, et al [24]  *Prevalence, age of onset and demographic relationships of different areca nut habits amongst children in Tower Hamlets, London* | Questionnaire   - Areca nut habit - Type of habit- - Areca nut chewing alone - Paan masala - Mistee paan - Betel quid   *Piloting of the questionnaire or its validity: not mentioned* | - Prevalence: 77% of the sample reported having used at least one habit. There was no difference in the prevalence of having used any of the habits between the ethnic populations studied (P = 0.7) - Ever used (type): areca nut alone is the most commonly used habit, with over 50% of boys and 70% of girl reporting use at some time. Pan masala is least commonly used, with less than 30% of the sample having used it at any time. Approximately 40% of the sample have used betel-quid and mistee pan at some time. - Ever used (Sex): Analyses of the effect of sex on habit individually revealed that the prevalence of ever-used areca nut alone was higher in girls than boys (P < .001) but that there were no sex differences in having used betel-quid, pan masala or mistee pan. - Current users: Of those currently engaged in an areca nut habit, 44% use one habit, 24% use two, 20% use three and 13% use all four habits. - Age at first use: For boys, the highest period of risk for engaging in the habit of areca nut alone, betel quid and mistee pan is between the ages of 5 and 10 in girls, the rate of uptake for areca nut alone, betel-quid and mistee pan remains at a largely constant rate between 5 and 12. - Frequency of use within current users: With the exception of pan masala in boys, the frequency of engaging in any of the habits is consistently between around 3 and 5 episodes per week for boys and girls. Pan masala however is used around 10 times a week by boys and it can be seen that boys used all habits more frequently than girls. |
| (2001) Pearson N, et al [25]  *Prevalence of oral lesions among a sample of Bangladeshi medical users aged 40 years and in Tower Hamlets, UK* | Questionnaire   - Habits related to paan chewing and tobacco smoking .   ‘Paan chewing'- was defined as chewing a mixture of betel leaf, areca nut and lime either with or without tobacco.  *Questionnaire was piloted and validated* | - Paan chewing was high among participants: 88 % among females and 69 % among males. - Tobacco was added to paan by 55 % of females and 30 % of males. - 53% of males and 5 % of females were tobacco smokers. - Overall, tobacco was used in some form by 60 % of females and 71 % or males. - Oral mucosal lesions were observed in 40% of participants. The most common lesion was found to be leukoplakia with a prevalence of 25%. - Significant relationships were found between smoking and the presence of oral pathology and between paan chewing with tobacco and the presence of leukoplakia. |
| (2001) Prabhu NT, et al [26]  *Betel quid chewing among Bangladeshi adolescents living in East London* | Questionnaire   - Betel quid chewing behaviours (frequency, age, ingredients) - Attitudes and perceptions about betel quid - Tobacco smoking   *Piloting of the questionnaire or its validity: not mentioned* | - Prevalence: 28% chewed betel quid, with no difference between males and females. The median age of starting quid habit was 9 years. 86% of chewers had started this habit in England. - Frequency of the chewers: 52% chewed betel quid most days, whereas 97% said they chewed at least every month. 81% chewed with their parents and also got betel quid to chew from their homes. - Ingredients: Most used betel leaf and nut. 7% added tobacco (all above 16 years) and few used pan masala. - Attitude: 38% of chewers had contemplated giving up quid habit, but only 5 people had given up in the past two months. Most had a negative attitude towards appearance of the mouth while chewing. - Predictive model for chewing behaviours: Adolescents who chewed pan with betel quid were more likely to agree that, it tasted good, had less negative attitude on the appearance of the teeth, were less likely to agree that it can cause cancer and were more likely to come from families where parents had lower education level. |
| (2013) Siddique I, et al [28]  *The impact of a community-based health education*  *programme on oral cancer risk factor awareness among*  *a Gujarati community* | Questionnaire   - Use of Tobacco, alcohol, paan, areca nut, paan masala and gutka. pan, gutka, etc - Knowledge on oral cancer   *Piloting of the questionnaire or its validity: not mentioned* | - Alcohol: Due to religious prohibition, Gujarati Muslims have very low declared alcohol consumption levels. 98% of first generation and 91% of second generation Gujarati Muslims denied alcohol intake. - Tobacco consumption: The difference in the proportions reporting tobacco consumption in 1^st^ generation Muslim Gujarati males (54%) compared with 2^nd^ generation males (26%) was 0.30 (p = 0.03) which was statistically significant. The difference in the proportions reporting tobacco use among 2^nd^ generation males (26%) compared with 2^nd^ generation females (14%) was 0.09 (p = 0.44). There was no regular tobacco use among first generation females. - Other product use: 1^st^ generation Gujarati Muslim males had the highest proportion of regular sopari users (33%), which was greater than their female counterparts (p = 0.11) and significantly greater than males in the 2^nd^ generation (p = 0.003). There was complete absence of regular Gutka use in Gujarati Muslims except among 1^st^ generation males (42%). After tobacco, Gutka consumption was the most common habit among this group of respondents. - Before and after: Significantly more first generation males and females correctly identified all oral cancer risk factors after the health education intervention compared with baseline (difference 0.40, 95% CI 0.23 to 0.57, p = <0.001). Significantly more second generation males and females correctly identified all oral cancer risk factors after the health education intervention compared with baseline (difference 0.45, 95% CI 0.28 to 0.61, p = <0.001) |
| (2016) Merchant R, et al [29]  *Oral cancer awareness in young South-Asian communities in*  *London* | Questionnaire   - Health-related behaviours - Use of tobacco, gutkha, paan with betel nut and alcohol (current or - previous) - Dental attendance - Knowledge and beliefs about oral cancer - Attendance pattern at a doctor or dentist   *Piloting of the questionnaire or its validity: not mentioned* | - Health related risks: Over half (58%) of the sample reported one or more health-related risk factors for oral cancer and 42% used tobacco, gutkha or paan in combination with alcohol. Of the 201 participants, 82 (41%) stated they had smoked cigarettes; 79 being current smokers (4 cigarettes/day) - Use of products: 10 participants (5%) reported that they chewed tobacco (those who currently chewed tobacco did so on average three times per day). 31 (15%) currently chewed (n=29), or used to chew (n=2) gutkha. 61 (30%) chewed paan with betel nut. Over half the sample (117, 58%) drank alcohol. - Dental Attendance: 8 of participants (4%) visited a dentist in the last year and36 (18%) in the past two years, with another 18% reporting never having visited a dentist. - Knowledge: Ethnicity and religion were associated with levels of knowledge of mouth cancer. ‘Low ‘ knowledge was associated with respondents of Pakistani origin and Bangladeshi origin (Pakistani=69%, odds ratio OR 0.41; Bangladeshi=85%, OR 0.16), Irrespective of the elevated risk of oral cancer, there was low awareness of the risk factors. Over half the respondents did not know that smoking Hukka, chewing paan, or betel nut increased risk of oral cancer. - Multivariable analyses indicated that chewing paan with betel nut (OR=4.08, 95%CI=1.58-10.59, p<0.01), and time since last visit to a dentist (OR=4.90, 95%CI=2.13-11.28, p<0.01) were independently associated with respondents level of knowledge of mouth cancer; the former positively and the latter negatively. |
| (2002) Ugur ZA, et al [30]  *Utilisation of dental services among a Turkish population in Witten, Germany.* | Interview   - Demography - Level of education - Proficiency in German language (The self-assessed proficiency in the German language was classified as low, middle or good) - Perceived condition of oral health - Use of dental services (People who made regular visits every year to have their teeth examined were described as the regular users of dental services otherwise irregular users of services) | - Dental attendance: Nineteen subjects (3.6 %) had never been to a dentist. The highest percentage (55.2 %) of regular users was observed in the youngest group. However, no statistically significant differences regarding the use of dental services were noted between the groups. - Type of dental care: All subjects in the study were covered by the German health insurance. Subjects who received dental care in Germany had a higher proportion of regular users in comparison to subjects who used dental services only in Turkey or both in Turkey and Germany (p<0.001). - Education and gender: Significant differences were observed between the different user groups and most selected individual factors. Thus, for higher level of education and German language, the proportion of regular users was higher, whereas no association was found for sex (p<0.001) - Dentist nationality: Nationality of the dentist in Germany was not associated with dental care pattern. Preferring Germany for dental care and perceiving better oral health were associated with more regular use of dental services (p<0.001). - Language proficiency: Logistic regression showed that a person with a good or middle level of German language was 2.21 times more likely to use dental services regularly (1.07, 4.57). If the subjects preferred to receive dental care in Germany (Odds Ratio= 1.99), there was an increased probability of having a regular utilisation habit (0.48, 2.24). The odds of regular use with respect to self-assessed oral health computed as 1.89 (1.08, 3.31). - Comparison to German data, DMS II indicated no significant difference between the age groups concerning utilization. |
| (2004) Van Steenkiste M, et al [32]  *Access to Oral Care and Attitudes to the Dentist by German and Turkish Parents* | Questionnaire based   - 11 items from the dental belief score (DBS) - 1 additional belief item: My dentist is good with kids - Questions regarding whether parents knew which treatments are payed for by the statutory health insurance, where there is an additional charge and which treatment is to be paid for privately - Access to dental care   *Questionnaire was validated but piloting not mentioned.* | - Attitude towards dentist/dental belief: Significant difference for all 12 questions between German and Turkish parents with worse perception of dentists among Turkish parents for all 12 questions (p<0.001) - Awareness (Financial questions): German parents were better informed about different costs and answered more correctly for 5/6 questions (p<0.01 /p<0.001 depending on the question) - Access to dental care: 8.2% German parents and 20.2% Turkish parents agreed that it was a problem for them to make an appointment at the dentists. (p >0.001). 12.2% German parents and 16.5% Turkish parents agrees that it was better if they could go to the dentists without an appointment. (p >0.001) - Barriers: 79.5% German parents and 81.2% Turkish parents disagreed that they were afraid to go to the dentists because of unknown costs. Agreement was similar in both groups (10.0% G; 9.2% T, p=0.13) |
| (2007) Schenk, L, et al [34]  *Oral health behaviour of children and adolescents in Germany. First results of the German Health Interview and Examination Survey for Children and Adolescents (KiGGS)* | Questionnaire   - Frequency of tooth-brushing - Frequency of dental visits - Use of pharmaceutical preparations for caries prevention   *Piloting of the questionnaire or its validity: not mentioned* | - Daily brushing: 29 % of participants brushed their teeth only once daily or less frequently. There was a SES gradient (low: 39 %, middle: 28 %, high: 22 %). This frequency of brushing was more frequently found in children with a migration background (45 %) than in those without a migration background (26 %). It was higher for migrants in all SES groups (low SES: 51% vs 34% for Germans, medium SES: 41% vs 21%, high SES: 32% vs 21%) - Dental attendance: 16.4% of migrants stated that they visited the dentist less than once a year, vs. 6.1% host population (p<0.05) - Use of dental products: 5.3% of migrants used pharmaceutical preparations for caries prevention, vs. 8.4% of host population (p<0.05) |
| (2017) Erdsiek F, et al [37]  *Oral health behaviour in migrant and non-migrant adults in Germany: the utilization of regular dental check-ups* | Telephone interview   - Demographics - Migration status - Utilization of dental check-ups in the 12 months prior to the interview   *Piloting of the questionnaire or its validity: not mentioned* | - Migrant respondents, more often had a lower socioeconomic status and were more often insured through statutory health insurance than host population and they lived significantly more often in West Germany and in urban areas. - Utilization of dental prevention was overall lower among migrants (72.6%) than host population (79.8%), corresponding to a crude odds ratio (OR) of 0.67. |
| (2017) Brzoska P, et al [38]  *Enabling and Predisposing Factors for the Utilization of Preventive Dental Health Care in Migrants and Non-Migrants in Germany.* | Telephone interview   - Predisposing factors (gender, age, SES) - Enabling factors (type of insurance, place of residence)   *Piloting of the questionnaire or its validity: not mentioned* | - Dental utilization: Migrants were at an about 36% lower chance of utilizing regular dental check-ups than host population, corresponding to an 8% point lower likelihood of utilization (OR = 0.64; AME = −0.081). Differences are partly explained by the influence of predisposing and enabling factors (OR = 0.69; AME = −0.065). Younger age, being male, lower SES, a non-statutory health insurance, and poor social support were associated with poor utilization of regular dental check-ups. - Although older individuals without migration background were less likely to utilize dental check-ups than younger individuals, it were older individuals among migrants who were more likely to utilize this form of dental prevention than younger respondents |
| (2018) Aarabi G, et al [39]  *Oral health and access to dental care – a comparison of elderly migrants and non-migrants in Germany.* | Interviews   - 18 questions corresponding to the German Oral Health Survey (DMS) IV. - Data on the migration history included country of birth, nationality and year of immigration. - Current smoking status was assessed. - Utilization of dental care services   *Piloting of the questionnaire or its validity: not mentioned* | - Reason for dental visits: In both groups, the most frequently indicated reason for a dentist consultation was prevention and/or check-up. - Dental attendance: Significantly more Germans than migrants reported that they would have a regular dentist that they visit (82.4% vs. 55.7%). 88.2% of Germans had visited a dentist at least once within the last year, while this was the case for only 68.9% of the migrants. - Oral hygiene aids: More non-migrants than migrants used additional oral hygiene aids aside from toothbrush and toothpaste. Specifically, inter-dental brushes were used by three quarters of the non-migrants, whereas only a third of migrants used these brushes regularly. - Barriers: Two thirds of the migrants reported difficulties in receiving dental treatment. The main reasons for this were cost concerns and language barriers, followed by anxiety. |
| (2000) Hjern A, et al [40]  *Dental Health and Access to Dental Care for Ethnic Minorities in Sweden* | Interviews   - Last dental treatment - Any longstanding illness - Impaired work capacity - Any caries in teeth   *Piloting of the questionnaire or its validity: not mentioned* | - Dental attendance: Adult migrants more often had been without dental treatment during the 24 months preceding the interview: 23% of the Chilean-born and the Turkish-born, 17% of the Iranian sample and 13% of the Polish-born compared with 8% of the Swedish born. - Dental problems: Women in the migrants group as well as the host population more often reported having problems of chewing as well as having been in dental treatment during the previous 24 months. - Summary: Respondents with poor education, economic stress and experience of unemployment more often reported problems of chewing and less often reported having received dental treatment during the previous 24 months in the host population as well as the migrants and their children more often were reported to have had caries. |
| (2001) Hjern A, et al [41]  *Social inequality in oral health and use of dental care in Sweden* | Interviews   - Use of Prosthesis - Chewing problems - Last dental treatment - Any caries in teeth   Past of the study by Hjern et al (2000)  *Piloting of the questionnaire or its validity: not mentioned* | - The odds of not having been in dental treatment during the 24 months were higher among migrants compared to host population (OR 1.6, ) CI-95% (1.3–2.0) .   *This study demonstrates that there is considerable social inequality in oral health and use of dental care in Sweden two decades after the introduction of a national dental insurance scheme* |
| (2004) Stecksén-Blicks C, et al [42]  *Caries Experience and Background Factors in 4-Year-Old Children: Time Trends 1967–2002* | Questionnaire   - Tooth brushing habits - Sugar consumption - Fluoride use   *Piloting of the questionnaire or its validity: not mentioned* | - Oral hygiene: Only 2 children had their teeth brushed twice or more per day (13%) compared to 52% among migrants (p <0.01). No significant difference was found in any of the variables reflecting sugar consumption. - Dental information: In the present study, only 50% of the migrant parents had attended dental health information meetings when their child was 2 years of age compared to 81% of the host population. - Sugar consumption: Increased between 1987-1997 in 4 year old. But since 1997-2002, no significant changes in consumption were noted. |
| (2005) Jacobsson B , et al [43]  *Dental caries and caries associated factors in Swedish 15-year-olds in relation to immigrant background.* | Questionnaire   - Oral Hygiene habits - Sugar consumption - Age of migration   *Piloting of the questionnaire or its validity: not mentioned* | - Oral hygiene: Among the migrants, 45 adolescents ( 88%) reported that they brushed their teeth with fluoride toothpaste at least twice a day compared to 65 (98%) among the host population. 6 migrants who brushed their teeth only once a day had more than twice as many proximal surfaces affected by caries as migrants who brushed twice a day [11.3 (3.2 -19.5) and 5.8 (4.0 -7.6), respectively; p=o.o4]. - Sugar consumption: The higher intake of snack products among migrants reflected a more frequent intake of various snack items. As a consequence of their higher intake frequency of snack products, their average daily estimated sugar intake was nearly twice as high [256 (189 - 324) grams/day] as among host population [144 (n8 -170) grams/day, p=o.oo3]. |
| (2008) Stecksén-Blicks C,et al [44]  *Caries prevalence and background factors in Swedish 4-year-old children – a 40-year perspective* | Questionnaire   - Oral hygiene practices - Dental attendance - General health info   *Piloting of the questionnaire or its validity: not mentioned* | - Oral hygiene: The frequency of tooth brushing was statistically significantly lower in migrant children (P < 0.01) compared to host population. - Sugar consumption: 18% of the migrant children were given sugared drinks every day compared to 3% in host population (P< 0.01), and sweets every day was given 6% of the migrant children compared to 0% in host children (P> 0.05). Drinking at night was more common in migrant children (P< 0.01). - Dental health information: 68% of the parents to migrant children had participated in dental health information at 2 years of age of the child compared to 79% of host population (parents) (P> 0.05).   *Since 2002, there has been a concurrent decrease in sugar in take in children between-meal products and increased tooth brushing frequency. In 2007, the caries distribution was skewed and migrant background had a significant association with caries prevalence and unfavourable health habits.*  *Part of series of cross sectional studies: (2004 ) Stecksén-Blicks C, et al.* |
| (2014) Stecksén-Blicks C, et al [47]  *Caries and background factors in Swedish 4-year-old children with special reference to immigrant status* | Questionnaire   - Medical conditions - Oral hygiene practices - Frequency of sugary snacks and drinks   *Piloting of the questionnaire or its validity: not mentioned* | - General health: The proportion of migrant children (16%) was not significantly different from that observed in 2007. In 12% of the children, a medical condition was reported, asthma and allergies being the most common. The host population had a significantly higher prevalence (p < 0.01) of asthma or allergies than migrant children had (12.3% vs 3%). - Oral hygiene: In 2012, 67% of the children received help with tooth brushing according to recommendations (twice a day), 22% received help once a day and 11% seldom or irregularly received help; there were no significant differences compared to 2007 (p > 0.05), but migrant background was associated with a statistically significantly lower frequency of tooth brushing (p < 0.05). - Sugar consumption: Dietary sugar intake did not correlate with dental caries in host population, but it was positively correlated with caries prevalence in migrant adolescents. The migrant children had a significantly higher intake of ice cream, sweets and chocolate drinks (p < 0.05). 18% of migrant children consumed ice cream three or more times per week compared with 5% of host children. Drinking at night was significantly (p < 0.05) more common in migrant children than in host population (children) (21% and 8%, respectively).   *Part of series of cross sectional studies: (2004 )and (2008) Stecksén-Blicks C, et al.* |
| (2016) Olerud E, et al [48]  *Oral health status in older immigrants in a medium-sized Swedish city* | Questionnaire   - Oral habits - Dental visits   *Piloting of the questionnaire or its validity: not mentioned* | - Dental attendance: More than half of the participants reported that they had visited the dentist in the last two years. Approximately the same proportion stated that they had problems with teeth or dentures. - Oral hygiene: Of the participants who had natural teeth, two-thirds reported that they brushed their teeth twice a day or more frequently, and all said that they used fluoride toothpaste. |
| (2018) Petti S, et al [53]  *Betel quid chewing among adult male immigrants from the Indian subcontinent to Italy* | Interview   - Attitudes and perceptions toward betel quid chewing   *Questionnaire was validated* | - Demographics: Most subjects were of Pakistani origin, 44% were routine smokers and started smoking in their country; 15% were alcohol drinkers, with 3.3% heavy drinkers above the threshold level of two daily drinks. - Combination with BQC: BQ chewing prevalence was close to 40%; most subjects were daily chewers of BQ with tobacco; 30% were chewers and smokers, and almost 80% of them had started smoking before starting the chewing habit. - Beliefs: Almost two-thirds of chewers believed that BQC helps to relieve stress, while only a smaller proportion of non-chewers (17%) stated that BQC would lead to stress relief. - Perception: Proportion of chewers who were aware that chewing is an unhealthy habit was higher than in non-chewers (62.5% vs 48.1%), while knowledge about oral carcinogenicity of BQC was lower among chewers (41.2% vs 46.6%). - Summary: The attitudinal factors significantly associated with chewing were being a routine smoker, being born to parents who were also chewers, the perception that chewing is good for health and that it helps to relieve stress. |
| (2006) Skeie MS,et al [55]  *Parental risk attitudes and caries-related behaviours among immigrant and western native children in Oslo* | Questionnaire to the parents.   - Parental indulgence - Attitude to hygiene - Attitude to diet - Caries experience   *Questionnaire was validated* | - Parents: When fathers of WN group were involved in answering the questionnaire (12.9% of the group), the d_1-5_mfs index was statistically significantly lower than when mothers alone responded (among 3-year olds: t = 2.33, d.f. -279, P = 0.021; among 5-year olds: t = 2.32, d.f. -108, P = 0.022). - Attitude to hygiene: Host parents had better attitude compered to migrants, more negative among Muslim parents (p<0.001). 12.4% of the host parents had caries promoting attitude to oral hygiene compared with 28.8% of the migrants. Within the migrant group, 47.4% (18/38 responders) of Muslims had this negative attitude against 8.6% of the parents without Muslim background (3/35 responders), a difference which was found to be statistically significant (x2= 13.38, d.f. -1, P < 0.001). - Attitude to diet- Migrant parents more often responded to be indulgent than those the host population (migrant: 45.1% vs. host population: 7.8%). The percentage of Muslims with this attitude was statistically significantly higher than that among the rest of the migrants (38.9% = 14/36 responders) vs. (8.6% = 3/35 responders; x2= 8.96, d.f. - 1,P= 0.003). - Predictors: Education level of mothers was statistically lower in Muslim groups than other remaining migrant group (p=0.028) although caries experience of children was not significantly related to mother’s education level. |
| (2008) Skeie MS, et al [56]  *Caries increment in children aged 3–5 years in relation to parents’ dental attitudes: Oslo, Norway 2002 to 2004* | Questionnaire to the parents.   - Parental indulgence - Attitude to hygiene - Attitude to diet - Caries experience   Follow up of study by Skeie MS et al 2006.  *Questionnaire was validated* | - Category of ‘Parental indulgence’: There were statistically significantly more children with migrant background than host population in this category with higher number of Muslim children. - Comparison between 2002-2004: Findings do not vary much except for- The parents who had negative attitude towards dental health in 2002 and 2004 had their children most affected by dental caries in migrant group. The combination of negative ‘Attitude to Diet’ (2002) and ‘Parental Indulgence’ (2002) could to some extent predict severe caries increment the two next years (from 3 to 5 years of age) with sensitivity 0.65 (95% CI: 0.52–0.76) and specificity 0.63 (95% CI: 0.60–0.66). The frequency of negative ‘Attitude to Hygiene’ was found to be lower in 2004 than in 2002 (P < 0.001), and a similar reduction was also traced in the other two parental attitudes, ‘Parental Indulgence’ and ‘Attitude to Hygiene’.   *Parental dental attitudes seem to be important for caries increment in young children, especially when negative attitudes regarding children’s dental health are relatively more frequent among migrants than host population.* |
| (2010) Skeie MS,et al [57]  *Tracking of parents’ attitudes to their children’s oral health-related behavior–Oslo, Norway, 2002–04* | Questionnaire same as above two studies   - Parental indulgence - Attitude to hygiene - Attitude to diet   Follow up of study by Skeie MS et al 2006, 2008 | - Prevalence of parental attitudes at baseline and follow-up: The distribution of negative attitudes among parents followed longitudinally showed that negative attitudes were consistently more frequent in the migrant group compared with the host population. In 2002, “Parental Indulgence” differed significantly between the two groups (x2 = 21.86; df = 1; p < 0.0001), while in 2004 “Parental Indulgence” (x2 = 70.43; df = 1; p < 0.0001). Similarly, “Attitude to Hygiene” (x2 = 14.60; df = 1; p < 0.0001) were significantly different. - Change in trends since 2002: 50 (71.4%) of the 70 host parents of 3-year-old children in 2002 who had a negative “Attitude to Hygiene” showed positive attitudes 2 years later. The small size of the migrant group (n = 31) did not justify detailed subgroup analyses.. - Risk attitude: Bivariate relationships, measured by the odds ratio (OR) between chosen independent variables and assignment to the group, showed significance for “Migrant Status” [OR 4.3; 95% confidence interval (CI) 1.9–9.9], “Education” (OR 4.2, CI 2.0–8.7), “Parental Dental Attendance” (OR 3.2, CI 1.4–6.9) and “Birth Order” (OR 2.1, CI 1.0–4.0), but not for “Single Parent” and “Mother’s Age”. - Parents not highly educated or with a non-Western background were most likely to be assigned to the “attitudinal risk group” and 87% of the migrants did not have a higher education. |
| (2010) Wigen TI, et al [58]  *Caries and background factors in Norwegian and immigrant 5-year-old children* | Questionnaire to the parents.   - Demographics - Oral health behaviour - Attitude to dental health   *Questionnaire was validated* | - Social determinants: More than half the children lived in families in which both parents had high education and the majority (87%) had two parents of western origin. The parents of 75% of the children reported favourable oral health behaviours, and parents of 90% of the children reported favourable attitudes towards oral health. - Predictors: Having one or both parents of non-western origin, one or both parents with low education, parents not willing to encourage tooth brushing and parents who brushed their own teeth once a day or less were the variables which showed the strongest association with caries experience (d_1–5_mft > 0) and dentine caries experience (d_3–5_mft > 0) at the age of five. - Adjusted for the other variables included in the analysis, having one or both parents of non-western origin (OR = 3.6), both parents (OR = 1.9) and one parent (OR = 1.7) of low educational level was statistically significantly associated with a higher probability of having caries experience (d_1–5_mft > 0) at the age of five. |
| (2007) Almerich-Silla JM, et al [60]  *Influence of immigration and other factors on caries in 12- and 15-yr-old children* | Questionnaire to the parents.   - Oral health related behaviours - Toothbrush frequency - Frequency of intake of cariogenic foods   *Questionnaire was validated* | - Oral hygiene: 12-yr-old children who did not brush their teeth daily, as they exhibited a higher DMFT count than those who did (P = 0.03), and in 15-yr-old children with a daily or almost daily intake of cariogenic foods, who also had a higher prevalence of caries (P = 0.011). - Predictors: Migration, age, daily tooth brushing, and intake of cariogenic foods between meals were variables that showed significant association, in a multiple linear regression model, with the DMFT count as the dependent variable. Of all the predictive variables, migration status presented the highest standardized beta coefficient (0.21), signifying a strong contribution to the model. |
| (2018) Muñoz‑Pino N, et al [61]  *Comparing Oral Health Services Use in the Spanish and Immigrant Working Population* | Questionnaire   - Health service utilization - Migration status - Sociodemographic variables   *Questionnaire was validated* | - Dental attendance: The migrant working population registered a lower proportion of oral health visits within 1 year prior (men: 33.6% and women: 41.7%) compared to the Spanish population (men: 45.0% and women: 50.2%). - Related to education level, those with the greatest prevalence of oral health service use more than 1 year prior were those with secondary education, with the greatest difference in prevalence in the migrant population (men: 74.9 and women: 75.5%), compared to the Spanish population (men: 69.9% and women: 64.0%). - SES: Also, there was greater prevalence of oral health service use 1 year or more prior in the manual social class, with a greater proportion among the migrant population (men: 80.0% and women: 79.5%), compared to the Spanish population (men: 59.2% and women: 53.6%). - Summary: The multivariate logistic regression models for oral health service use, which adjusts the principal independent variable “migration status” by age, shows that migrants have a greater probability of oral health service use 1 year or more prior than Spaniards (men OR 1.63; IC95% 1.30–2.05, women OR 1.41; IC95% 1.13–1.76). |
| (2019) Valcarcel Soria R, et al [62]  *Acculturation and Dental Caries Among Children in Spain.* | Questionnaire  Acculturation: measured using four proxy measures:  generational status, age at arrival (before age 6 years vs.  at age 6 years or older), length of residence in Spain (less  than 10 years vs. 10 or more years) and language spoken at  home (Spanish only, Spanish and other language, and other  language only)  *Questionnaire validity not mentioned* | - By age at arrival, first-generation migrant children arriving in Spain before 6 years of age had 34% (17–53%) greater caries experience than Spanish-born children. - By length of residence, first-generation migrant children with 10 or more years in Spain had 38% (18–61%) greater caries experience than Spanish-born children. - By language spoken at home, children in families speaking Spanish and other language and children in families speaking other language only had, respectively, 31% (14–50%) and 25% (9–83%) greater caries experience than those in Spanish-speaking families. - Overall, inequalities in dental caries between migrant and host children were evident (favouring the host population). First-generation migrant, those who arrived in Spain at an early age, who have been living in Spain for a longer time and who spoke a language other than Spanish had more teeth with caries experience. |
| (2015) Duijster D, et al [63]  *Parental and family-related influences on dental caries in children of Dutch, Moroccan and Turkish origin.* | Questionnaire   - Oral health behaviours-tooth brushing frequency, the age tooth brushing was started, parental involvement with tooth brushing. - Frequency of consumption of sugary foods and drinks between meals. - LoC-parental beliefs and attitudes associated with children’s oral health behaviours - Parenting practices- The self-report Alabama Parenting Questionnaire (APQ) was used. Parenting practices were also observed using Structured Interaction Tasks (SIT).   *Questionnaire was validated* | - Dental self-efficacy: A higher dental self-efficacy was significantly associated with a more internal LoC (Pearson’s r = 0.41). Dental self-efficacy and LoC were also moderately correlated with several (observed) parenting dimensions, including positive involvement, encouragement and problem-solving. In particular, strong associations were found for encouragement with problem-solving and coercion (r = 0.59 and r = 0.59, respectively), and for problem-solving with interpersonal atmosphere (r = 0.60). Overall, Dutch parents had better dental self-efficacy compared to migrant parents. - LoC: Parents’ LoC was significantly more internal in controls than in cases. Dutch parents had a more internal LoC (32.7 ± 5.8) compared to Moroccan parents (29.0 ± 7.4, P = 0.004) and compared to Turkish parents (25.5 ± 7.9, P < 0.001). - Sugar consumption: In terms of oral health behaviours, cases reported more frequent consumption of sugary foods between meals compared to controls (although this was only a trend, P = 0.06).   *Overall, parents of children from higher social classes had a higher dental self-efficacy and a more internal LoC. They also showed higher levels of positive involvement and encouragement, better problem-solving and a better interpersonal atmosphere during interactions with their child, and they were less likely to show coercive behaviors.* |
| (2003) Sundby A, et al [65]  *Oral health status in relation to ethnicity of children in the*  *Municipality of Copenhagen, Denmark* | Questionnaire   - Demographics: parental education, occupation, marital status, Danish language competence - Living conditions and oral hygiene practices.   *Pre tested questionnaire but b validity not mentioned* | - Oral hygiene: Migrant parents started brushing their child’s teeth later compared to Danish parents; the same parents also stopped helping their children clean the teeth at a younger age. Among 7-year-olds, 84% of the Danish children had help from adults in daily tooth brushing whereas only 32% of the Pakistani children, 40% of the Arabian children and 48% of the Turkish children had daily help in tooth brushing from parents. - Attitude towards oral health: 35% of the parents of Arabian 3-year-old children held the attitude that kindergartens were responsible for their child’s dental health. - Sugar consumption: Migrant children consumed sweets or sweetened food relatively often and this was particularly seen for children aged 3 years. Among 3–5-years-old children, 28% of the Danish children had fruit syrup every day or several times a day as compared to 50% of the Albanian children |
| (2014) Cvikl B, et al [69]  *Migration background is associated with caries in Viennese school children, even if parents have received a higher education.* | Questionnaire   - Migration background - Education level of parents   (Through ‘Statistics Denmark’ institution of the government) | - Migration background and education level: A significant interaction was found between the migration background and the educational level of parents (p = 0.045). The mean DMFT index was 2.18 and 1.87, respectively, among low and high/ medium educational level. These data suggest that migration background as well as the educational level of parents affects the caries status of their children. - Data suggest that children with a migration background are at higher risk of acquiring caries than the host population even if their parents have a higher educational level. |
