# Supplementary material for: Oral Health Status, Oral Health Behaviours and Oral Health Care Utilisation Among Migrants Residing in Europe: A Systematic Review
Source: J Immigr Minor Health. 2020 Jul 19;23(2):373–88. doi: 10.1007/s10903-020-01056-9 (PMC7914188; doi:10.1007/s10903-020-01056-9)
Supplement: Supplementary file 5 — Supplementary file5 (DOCX 48 kb) [file 10903_2020_1056_MOESM5_ESM.docx]

**Appendix table 5: Critical appraisal of all the studies for quality of study design (7 components) using AXIS tool (detailed)**

| **Year of publication and Authors** | **1** | **2** | **3** | **4** | **5** | **6** | **7** |
| --- | --- | --- | --- | --- | --- | --- | --- |
|  | **Study design** | **Sample size**  **justification** | **Sample frame** | **Risk factors and outcomes** | **Discussion** | **Funding or conflict of interest** | **Ethical approval or Consent form** |
| (2000) Robinson PG, et al [1] | ● | ○ | ○ | ● | ● | FS-Mentioned  CI- Not declared | ● |
| (2000) Newton JT, et al. [2] | ● | ○ | ○ | ● | ● | FS-Mentioned  CI- Not declared | ● |
| (2000) Gray M, et al [3] | ● | ○ | ○ | ● | ● | FS-Mentioned  CI- Not declared | ○ |
| (2001) Pau AKH, et al [4] | ● | ○ | ○ | ● | ● | FS-Mentioned  CI- Not declared | EA- Not mentioned  CF- Implicit |
| (2001) Ahmed B, et al [5] | ● | ○ | ○ | ● | ● | FS-Mentioned  CI- Not declared | ○ |
| (2003) Newton JT, et al [6] | ● | ○ | ○ | ● | ● | FS-Mentioned  CI- Not declared | ○ |
| (2005) Dugmore CR, et al [7] | ● | ● | ● | ● | ● | FS-Mentioned  CI- Not declared | ● |
| (2005) Alkhatib MN, et al [8] | ● | ○ | ○ | ● | ● | FS-Mentioned  CI- Not declared | ● |
| (2007) Conway DI, et al [9] | ● | ● | ● | ● | ● | ○ | ● |
| (2008) Hullah E, et al [10] | ● | ○ | ○ | ● | ● | ○ | ○ |
| (2011) Reekie T. [11] | ● | ○ (Secondary data from one cleft clinic) | ○ | ● | ● | ● | ● |
| (2013) Marcenes W, et al [12] | ● | ● | ● | ● | ● | ○ | ● |
| (2013) Al-Haboubi M, et al [13] | ● | ● | ● | ● | ● | FS-Not mentioned  CI- Declared as none | ● |
| (2014) Choa RM,et al [14] | ● | ○ (Secondary data from three cleft clinic) | ○ | ● | ● | ● | ● |
| (2015) Delgado-Angulo EK, et al [15] | ● | ● | ● | ● | ● | FS-Mentioned  CI- Not declared | ● |
| (2016) Delgado-Angulo EK, et al [16] | ● | ● | ● | ● | ● | FS-Mentioned  CI- Not declared | ● |
| (2017) Abdelrahim R, et al [17] | ● | ● | ● | ● | ● | ○ | ● |
| (2017) Arora G, et al [18] | ● | ● | ● | ● | ● | ● | ● |
| (2018) Weston-Price S, et al [19] | ● | ● | ● | ● | ● | ○ | EA- Not mentioned  CF- Taken |
| (2018) Delgado-Angulo E.K, et al [20] | ● | ● | ● | ● | ● | ○ | ● |
| (2018) Rouxel P, et al [21] | ● | ● | ● | ● | ● | ○ | ● |
| (2000) Khan FA,et al [22] | ● | ● | ○ | ● | ● | ○ | ● |
| (2000) Vora A.R, et al [23] | ● | ○ | ○ | ● | ● | ○ | ● |
| (2001) Farrand P, et al [24] | ● | ● | ○ | ● | ● | ○ | EA- Not mentioned  CF- Taken |
| (2001) Pearson N, et al [25] | ● | ○ | ○ | ● | ● | ○ | ● |
| (2001) Prabhu NT, et al [26] | ● | ○ | ○ | ● | ● | ○ | ● |
| (2013) Csikar J,et al [27] | ● | ● | ● | ● | ● | ○ | Not applicable |
| (2013) Siddique I, et al [28] | ● | ○ | ○ | ● | ● | ○ | ○ |
| (2016) Merchant R, et al [29] | ● | ○ | ○ | ● | ● | ○ | ● |
| (2002) Ugur ZA, et al [30] | ● | ○ | ○ | ● | ● | ○ | EA- Not mentioned  CF- Taken |
| (2003) Kühnisch, J, et al [31] | ● | ○ | ○ | ● | ● | ○ | ○ |
| (2004) Van Steenkiste M, et al [32] | ● | ● | ● | ● | ● | ○ | ○ |
| (2004) Van Steenkiste M,et al [33] | ● | ● | ● | ● | ● | ○ | ○ |
| (2007) Schenk, L, et al [34] | ● | ● | ● | ● | ● | FS-Mentioned  CI- Not declared | ● |
| (2007) Heinrich-Weltzien R, et al [35] | ● | ○ | ● | ● | ● | FS-Mentioned  CI- Not declared | ○ |
| (2007) Bissar AR, et al [36] | ● | ○ | ○ | ● | ● | FS-Mentioned  CI- Not declared | ● |
| (2017) Erdsiek F, et al [37] | ● | ○ | ● | ● | ● | ● | EA- Not mentioned  CF- Taken |
| (2017) Brzoska P, et al [38] | ● | ● | ○ | ● | ● | ● | ● |
| (2018) Aarabi G, et al [39] | ● | ○ | ● | ● | ● | ● | EA- Not mentioned  CF- Taken |
| (2000) Hjern A, et al [40] | ● | ● | ● | ● | ● | FS-Mentioned  CI- Not declared | ● |
| (2001) Hjern A, et al [41] | ● | ● | ● | ● | ● | FS-Mentioned  CI- Not declared | ○ |
| (2004) Stecksén-Blicks C, et al [42] | ● | ● | ● | ● | ● | FS-Mentioned  CI- Not declared | EA- Not mentioned  CF- Taken |
| (2005) Jacobsson B , et al [43] | ● | ● | ○ | ● | ● | FS-Mentioned  CI- Not declared | EA- Mentioned  CF- Not taken |
| (2008) Stecksén-Blicks C,et al [44] | ● | ● | ● | ● | ● | FS-Mentioned  CI- Not declared | EA- Not mentioned  CF- Taken |
| (2010) Julihn A, et al [45] | ● | ● | ● | ● | ● | FS-Mentioned  CI- Not declared | EA- Not mentioned  CF- Taken |
| (2010) Mousavi SM, et al [46] | ● | ● | ● | ● | ● | ○ | EA- Not mentioned  CF- Not applicable |
| (2014) Stecksén-Blicks C, et al [47] | ● | ● | ● | ● | ● | ● | EA- Not mentioned  CF- Taken |
| (2016) Olerud E, et al [48] | ● | ○ | ○ | ● | ● | FS-Mentioned  CI- Not declared | ● |
| (2004) Ferro R,et al [49] | ● | ○ | ○ | ● | ○ | ○ | ○ |
| (2007) Ferro R, et al [50] | ● | ○ | ● | ● | ● | ○ | ● |
| (2007) Ferro R,et al [51] | ● | ○ | ● | ● | ● | ○ | ● |
| (2007) Ferro R, et al [52] | ● | ○ | ● | ● | ● | ○ | EA- Not mentioned  CF- Taken |
| (2018) Petti S, et al [53] | ● | ○ | ○ | ● | ● | FS-Not mentioned  CI- Declared as none | ○ |
| (2005) Skeie MS, et al [54] | ● | ● | ● | ● | ● | FS-Mentioned  CI- Not declared | ● |
| (2006) Skeie MS,et al [55] | ● | ● | ○ | ● | ● | FS-Mentioned  CI- Not declared | ● |
| (2008) Skeie MS, et al [56] | ● | ● | ○ | ● | ● | FS-Mentioned  CI- Not declared | ● |
| (2010) Skeie MS,et al [57] | ● | ● | ○ | ● | ● | FS-Mentioned  CI- Not declared | ● |
| (2010) Wigen TI, et al [58] | ● | ● | ● | ● | ● | ○ | ● |
| (2006) Almerich Silla JM, et al [59] | ● | ● | ● | ● | ● | ○ | ○ |
| (2007) Almerich-Silla JM, et al [60] | ● | ● | ● | ● | ● | ○ | EA- Not mentioned  CF- Taken |
| (2018) Muñoz‑Pino N, et al [61] | ● | ● | ● | ● | ● | ● | ● |
| (2019) Valcarcel Soria R, et al [62] | ● | ● | ○ | ● | ● | FS-Not mentioned  CI- Declared as none | ● |
| (2015) Duijster D, et al [63] | ● | ● | ○ | ● | ● | FS-Mentioned  CI- Not declared | ● |
| (2016) van der Tas JT, et al [64] | ● | ● | ● | ● | ● | ● | ● |
| (2003) Sundby A, et al [65] | ● | ○ | ○ | ● | ● | ○ | ○ |
| (2010) Christensen LB, et al [66] | ● | ● | ● | ● | ● | ○ | ● |
| (2011) Gatou T, et al [67] | ● | ● | ● | ● | ● | ○ | EA- Mentioned  CF- Not mentioned |
| (2017) Mantonanaki. M, et al [68] | ● | ● | ● | ● | ● | ○ | ● |
| (2014) Cvikl B, et al [69] | ● | ● | ● | ● | ● | ○ | ● |
| **TOTAL (%)** | **69/69 =100%** | **41/69=59.4%** | **38/69=55.0%** | **69/69=100%** | **68/69=98.5%** | **9/69= 13.0%** | **39/69= 56.5%** |

Criteria satisfied ● Criteria not satisfied ○

**Appendix table 6: Critical appraisal of all the studies for quality of reporting (7 components) using AXIS tool (detailed)**

| **Year of publication and Authors** | **1** | **2** | **3** | **4** | **5** | **6** | **7** |
| --- | --- | --- | --- | --- | --- | --- | --- |
|  | **Aim** | **Target population** | **Statistical significance** | **Methods used reproducible** | **Basic results described** | **Results for given analysis reported** | **Limitations** |
| (2000) Robinson PG, et al [1] | ● | ● | ● | ○ (Clinical examination was correct, but questionnaire was neither piloted nor validated) | ● | ● | ● |
| (2000) Newton JT, et al. [2] | ● | ● | ● | ● | ● | ● | ● |
| (2000) Gray M, et al [3] | ● | ● | ● | ● | ● | ● | ● |
| (2001) Pau AKH, et al [4] | ● | ● | ● | ● | ● | ● | ● |
| (2001) Ahmed B, et al [5] | ● | ● | ● | ○(Clinical examination was correct, but questionnaire- not piloted or validated) | ● | ● | ○ |
| (2003) Newton JT, et al [6] | ● | ● | ● | ● | ● | ● | ● |
| (2005) Dugmore CR, et al [7] | ● | ● | ● | ● | ● | ● | ○ |
| (2005) Alkhatib MN, et al [8] | ● | ● | ● | ○( Sample frame not clear, questionnaire results not mentioned clearly) | ○ Results of questionnaire not mentioned | ○ Questionnaire results not mentioned | ○ |
| (2007) Conway DI, et al [9] | ● | ● | ● | ● | ● | ● | ○ |
| (2008) Hullah E, et al [10] | ● | ● | ○ no mention of any type of statistical method used | ○ (Questionnaire piloting and validity not mentioned) | ● | ● | ● |
| (2011) Reekie T. [11] | ● | ● | ● | ● | ○ (Results not described in text clearly) | ● | ○ |
| (2013) Marcenes W, et al [12] | ● | ● | ● | ● | ● | ● | ● |
| (2013) Al-Haboubi M, et al [13] | ● | ● | ● | ○ (questionnaire- not piloted) | ● | ● | ● |
| (2014) Choa RM,et al [14] | ● | ● | ● | ● | ● | ● | ● |
| (2015) Delgado-Angulo EK, et al [15] | ● | ● | ● | ● | ● | ● | ● |
| (2016) Delgado-Angulo EK, et al [16] | ● | ● | ● | ● | ● | ● | ● |
| (2017) Abdelrahim R, et al [17] | ● | ● | ● | ● | ● | ● | ● |
| (2017) Arora G, et al [18] | ● | ● | ● | ● | ● | ● | ● |
| (2018) Weston-Price S, et al [19] | ● | ● | ● | ● | ● | ● | ○ |
| (2018) Delgado-Angulo E.K, et al [20] | ● | ● | ● | ○ (Clinical examination was correct, but questionnaire- not validated) | ○ (Results on questionnaire not described) | ○ (Results on questionnaire not described) | ● |
| (2018) Rouxel P, et al [21] | ● | ● | ● | ● | ● | ● | ● |
| (2000) Khan FA,et al [22] | ● | ● | ● | ● | ● | ● | ● |
| (2000) Vora A.R, et al [23] | ● | ● | ● | ○ | ● | ● | ○ |
| (2001) Farrand P, et al [24] | ● | ● | ● | ○ | ● | ● | ● |
| (2001) Pearson N, et al [25] | ● | ● | ● | ● | ● | ● | ○ |
| (2001) Prabhu NT, et al [26] | ● | ● | ● | ○ | ● | ● | ● |
| (2013) Csikar J,et al [27] | ● | ● | ● | ● | ● | ● | ○ |
| (2013) Siddique I, et al [28] | ● | ● | ● | ○ | ● | ● | ● |
| (2016) Merchant R, et al [29] | ● | ● | ● | ○ | ● | ● | ● |
| (2002) Ugur ZA, et al [30] | ● | ● | ● | ○ (Clinical examination was correct, but questionnaire- not validated) | ● | ● | ● |
| (2003) Kühnisch, J, et al [31] | ● | ● | ● | ● | ● | ● | ○ |
| (2004) Van Steenkiste M, et al [32] | ● | ● | ● | ● | ● | ● | ● |
| (2004) Van Steenkiste M,et al [33] | ● | ● | ● | ● | ● | ● | ● |
| (2007) Schenk, L, et al [34] | ● | ● | ● | ● | ● | ● | ○ |
| (2007) Heinrich-Weltzien R, et al [35] | ● | ● | ● | ● | ● | ● | ○ |
| (2007) Bissar AR, et al [36] | ● | ● | ● | ● | ● | ● | ○ |
| (2017) Erdsiek F, et al [37] | ● | ● | ● | ● | ● | ● | ● |
| (2017) Brzoska P, et al [38] | ● | ● | ● | ● | ● | ● | ● |
| (2018) Aarabi G, et al [39] | ● | ● | ● | ● | ● | ● | ● |
| (2000) Hjern A, et al [40] | ● | ● | ● | ○ | ○ | ● | ○ |
| (2001) Hjern A, et al [41] | ● | ● | ● | ○ | ● | ● | ○ |
| (2004) Stecksén-Blicks C, et al [42] | ● | ● | ● | ○ (Clinical examination was correct, but questionnaire- not validated) | ● | ● | ○ |
| (2005) Jacobsson B , et al [43] | ● | ● | ● | ○ (Clinical examination was correct, but questionnaire- not validated) | ● | ● | ○ |
| (2008) Stecksén-Blicks C,et al [44] | ● | ● | ● | ○ (Clinical examination was correct, but questionnaire- not validated) | ● | ● | ○ |
| (2010) Julihn A, et al [45] | ● | ● | ● | ○ (Clinical examination was correct, but questionnaire- not validated) | ● | ● | ● |
| (2010) Mousavi SM, et al [46] | ● | ● | ● | ● | ● | ● | ○ |
| (2014) Stecksén-Blicks C, et al [47] | ● | ● | ● | ○ (Clinical examination was correct, but questionnaire- not validated) | ● | ● | ○ |
| (2016) Olerud E, et al [48] | ● | ● | ● | ● | ● | ● | ● |
| (2004) Ferro R,et al [49] | ● | ● | ● | ○ | ○ | ○ | ● |
| (2007) Ferro R, et al [50] | ● | ● | ● | ● | ● | ● | ○ |
| (2007) Ferro R,et al [51] | ● | ● | ● | ● | ● | ● | ○ |
| (2007) Ferro R, et al [52] | ● | ● | ● | ● | ● | ● | ○ |
| (2018) Petti S, et al [53] | ● | ● | ● | ● | ● | ● | ○ |
| (2005) Skeie MS, et al [54] | ● | ● | ● | ● | ● | ● | ○ |
| (2006) Skeie MS,et al [55] | ● | ● | ● | ● | ● | ● | ○ |
| (2008) Skeie MS, et al [56] | ● | ● | ● | ● | ● | ● | ○ |
| (2010) Skeie MS,et al [57] | ● | ● | ● | ● | ● | ● | ○ |
| (2010) Wigen TI, et al [58] | ● | ● | ● | ● | ● | ● | ○ |
| (2006) Almerich Silla JM, et al [59] | ● | ● | ● | ● | ● | ○ | ○ |
| (2007) Almerich-Silla JM, et al [60] | ● | ● | ● | ● | ● | ● | ○ |
| (2018) Muñoz‑Pino N, et al [61] | ● | ● | ● | ○ | ● | ● | ● |
| (2019) Valcarcel Soria R, et al [62] | ● | ● | ● | ○ | ● | ● | ● |
| (2015) Duijster D, et al [63] | ● | ● | ● | ● | ● | ● | ● |
| (2016) van der Tas JT, et al [64] | ● | ● | ● | ● | ● | ● | ● |
| (2003) Sundby A, et al [65] | ● | ● | ● | ● | ● | ● | ○ |
| (2010) Christensen LB, et al [66] | ● | ● | ● | ● | ● | ● | ○ |
| (2011) Gatou T, et al [67] | ● | ● | ● | ● | ● | ● | ○ |
| (2017) Mantonanaki. M, et al [68] | ● | ● | ● | ● | ● | ● | ○ |
| (2014) Cvikl B, et al [69] | ● | ● | ● | ● | ● | ● | ● |
| **TOTAL (%)** | **69/69=100%** | **69/69=100%** | **68/69=98.5%** | **47/69= 68.1%** | **64/69= 92.7%** | **65/69=94.2%** | **34/69= 49.2%** |

Criteria satisfied ● Criteria not satisfied ○

**Appendix table 7: Critical appraisal of all the studies for risk of bias (6 components) using AXIS tool (detailed)**

| **Year of publication and Authors** | **1** | **2** | **3** | **4** | **5** | **6** |
| --- | --- | --- | --- | --- | --- | --- |
|  | **Representativeness** | **Measures to address non-responders** | **Measures of risk factors and outcome variables** | **Concerns on non-response** | **Information on non-responders** | **Internally consistent results** |
| (2000) Robinson PG, et al [1] | ○ | ○ | ○ (No mention of questionnaire validity) but piloting was done | ● (83.4 % Response rate) | ○ | ● |
| (2000) Newton JT, et al. [2] | ○ | ○ | ● | ○dk  (Not mentioned) | ○ | ● |
| (2000) Gray M, et al [3] | ○ | ○ | ● | ○dk  (Not mentioned) | ○ | ● |
| (2001) Pau AKH, et al [4] | ○ | ○ | ● | ○dk  (Not mentioned) | ○ | ● |
| (2001) Ahmed B, et al [5] | ○ | ○ | ○ (No mention of questionnaire piloting or validity) | ○dk  (Not mentioned) | ○ | ● |
| (2003) Newton JT, et al [6] | ○ | ○ | ● | ○dk  (Not mentioned) | ○ | ● |
| (2005) Dugmore CR, et al [7] | ● | ○ | ● | ○dk  (Not mentioned) | ○ | ● |
| (2005) Alkhatib MN, et al [8] | ○ | ○ | ○ | ● (80% Response rate) | ○ | ○ |
| (2007) Conway DI, et al [9] | ● | ○ | ● | ● (90% Response rate) | ○ | ● |
| (2008) Hullah E, et al [10] | ○ | ○ | ○ | ○dk  (Not mentioned) | ○ | ● |
| (2011) Reekie T. [11] | ○ | ○ not applicable | ● | ○ not applicable | ○ not applicable | ● |
| (2013) Marcenes W, et al [12] | ● | ○ | ● | ○ (52.8 % Response rate) | ○ | ● |
| (2013) Al-Haboubi M, et al [13] | ● | ○ | ○ (questionnaire piloting) not done | ○ (56% Response rate) | ○ | ● |
| (2014) Choa RM,et al [14] | ○ | ○not applicable | ● | ○ not applicable | ○ not applicable | ● |
| (2015) Delgado-Angulo EK, et al [15] | ● | ● | ● | ○ (57 % Response rate) | ● | ● |
| (2016) Delgado-Angulo EK, et al [16] | ● | ● | ● | ○ (57 % Response rate) | ● | ● |
| (2017) Abdelrahim R, et al [17] | ● | ○ | ● | ○ (56% Response rate) | ○ | ● |
| (2017) Arora G, et al [18] | ● | ○ not applicable | ● | ● (60 % Response rate) | ○ not applicable | ● |
| (2018) Weston-Price S, et al [19] | ● | ○ | ● | ● (90 % Response rate) | ○ | ● |
| (2018) Delgado-Angulo E.K, et al [20] | ● | ○ | ○ (Results on questionnaire not described) | ○ (57% Response rate) | ○ | ○ (Results on questionnaire not described) |
| (2018) Rouxel P, et al [21] | ● | ○ | ● | ● (89% Response rate) | ○ | ● |
| (2000) Khan FA,et al [22] | ○ | ○ | ● | ○dk  (Not mentioned) | ○ | ● |
| (2000) Vora A.R, et al [23] | ○ | ○ | ○ | ○dk  (Not mentioned) | ○ | ● |
| (2001) Farrand P, et al [24] | ○ | ○ | ○ | ○dk  (Not mentioned) | ○ | ● |
| (2001) Pearson N, et al [25] | ○ | ○ | ● | ● (85% Response rate) | ○ | ● |
| (2001) Prabhu NT, et al [26] | ○ | ○ | ● | ● (70.3% Response rate) | ○ | ● |
| (2013) Csikar J,et al [27] | ● | ○ not applicable | ● | ○  (Secondary data) | ○ not applicable | ● |
| (2013) Siddique I, et al [28] | ○ | ○ | ○ | ○dk  (Not mentioned) | ○ | ● |
| (2016) Merchant R, et al [29] | ○ | ○ | ○ | ○dk  (Not mentioned) | ○ | ● |
| (2002) Ugur ZA, et al [30] | ○ | ○ | ○ | ● (96.7% Response rate) | ○ | ● |
| (2003) Kühnisch, J, et al [31] | ○ | ○ | ● | ○dk  (Not mentioned) | ○ | ● |
| (2004) Van Steenkiste M, et al [32] | ● | ○ | ● | ○dk  (Not mentioned) | ○ | ● |
| (2004) Van Steenkiste M,et al [33] | ● | ○ | ● | ○dk  (Not mentioned) | ○ | ● |
| (2007) Schenk, L, et al [34] | ● | ● | ● | ● (66.6% Response rate) | ● | ● |
| (2007) Heinrich-Weltzien R, et al [35] | ○ | ○ | ● | ○dk  (Not mentioned) | ○ | ● |
| (2007) Bissar AR, et al [36] | ○ | ○ | ● | ○dk  (Not mentioned) | ○ | ● |
| (2017) Erdsiek F, et al [37] | ● | ○ | ● | ○dk  (Not mentioned) | ○ | ● |
| (2017) Brzoska P, et al [38] | ○ | ○ | ● | ○dk  (Not mentioned) | ○ | ○ |
| (2018) Aarabi G, et al [39] | ○ | ○ | ● | ● (83% Response rate) | ○ | ● |
| (2000) Hjern A, et al [40] | ● | ○ | ● | ● (More than 60% response rate in all groups) | ○ | ● |
| (2001) Hjern A, et al [41] | ● | ○ | ● | ○dk  (Not mentioned) | ○ | ● |
| (2004) Stecksén-Blicks C, et al [42] | ● | ○ | ● | ○dk  (Not mentioned) | ○ | ● |
| (2005) Jacobsson B , et al [43] | ○ | ○ | ○ | ○dk  (Not mentioned) | ○ | ● |
| (2008) Stecksén-Blicks C,et al [44] | ● | ○ | ● | ○dk  (Not mentioned) | ○ | ● |
| (2010) Julihn A, et al [45] | ● | ○ | ● | ● (86% Response rate) | ● | ● |
| (2010) Mousavi SM, et al [46] | ● | ○ not applicable | ● | ○ not applicable | ○ not applicable | ● |
| (2014) Stecksén-Blicks C, et al [47] | ● | ○ | ● | ○dk  (Not mentioned) | ○ | ● |
| (2016) Olerud E, et al [48] | ○ | ○ | ○ | ○dk  (Not mentioned) | ○ | ● |
| (2004) Ferro R,et al [49] | ○ | ○ | ○ | ○dk  (Not mentioned) | ○ | ○ |
| (2007) Ferro R, et al [50] | ● | ○ | ● | ● (73% Response rate) | ○ | ● |
| (2007) Ferro R,et al [51] | ● | ○ | ● | ● (81% Response rate) | ○ | ● |
| (2007) Ferro R, et al [52] | ● | ○ | ● | ● (More than 80% response rate) | ○ | ● |
| (2018) Petti S, et al [53] | ○ | ○ | ● | ● (72% Response rate) | ○ | ● |
| (2005) Skeie MS, et al [54] | ● | ○ | ● | ● (86.1% Response rate) | ○ | ● |
| (2006) Skeie MS,et al [55] | ○ | ● | ● | ● (More than 60% response rate in both the groups) | ● | ● |
| (2008) Skeie MS, et al [56] | ○ | ● | ● | ● (More than 75% response rate in both the groups) | ● | ● |
| (2010) Skeie MS,et al [57] | ○ | ● | ● | ● (More than 75% response rate in both the groups) | ● | ● |
| (2010) Wigen TI, et al [58] | ● | ○ | ● | ○dk  (Not mentioned) | ○ | ● |
| (2006) Almerich Silla JM, et al [59] | ● | ○ | ● | ○dk  (Not mentioned) | ○ | ○ |
| (2007) Almerich-Silla JM, et al [60] | ● | ○ | ● | ○dk  (Not mentioned) | ○ | ● |
| (2018) Muñoz‑Pino N, et al [61] | ● | ○ | ● | ● (Response rate 71.06 %) | ○ | ● |
| (2019) Valcarcel Soria R, et al [62] | ○ | ○ | ○ | ● (Response rate 94%) | ○ | ● |
| (2015) Duijster D, et al [63] | ○ | ○ | ● | ○ (Response rate 34%) | ● | ● |
| (2016) van der Tas JT, et al [64] | ● | ○ | ● | ○ (Response rate 44.1%) | ○ | ● |
| (2003) Sundby A, et al [65] | ○ | ● | ● | ● (Response rate 63.5%) | ● | ● |
| (2010) Christensen LB, et al [66] | ● | ○ not applicable | ● | ● (Response rate 76%) | ○ not applicable | ● |
| (2011) Gatou T, et al [67] | ● | ○ | ● | ● (Response rate 65.8%) | ○ | ● |
| (2017) Mantonanaki. M, et al [68] | ● | ○ | ● | ○(Response rate 58%) | ○ | ● |
| (2014) Cvikl B, et al [69] | ● | ○ | ● | ○dk  (Not mentioned) | ○ | ● |
| **TOTAL (%)** | **36/69=52.1%** | **7/69= 10.1%** | **54/69= 78.2%** | **26/69= 37.6% raise no concerns** | **9/69= 13.0%** | **64/69=92.7%** |

Criteria satisfied ● Criteria not satisfied ○

**Appendix table 8: Summary of critical appraisal of all the studies**

|  | **Domain 1: Quality of study design** | **Domain 2: Quality of reporting** | **Domain 3: Risk of bias** |
| --- | --- | --- | --- |
| (2000) Robinson PG, et al [1] | Sample size- not justified  Sample frame- convenience sampling  CI- not mentioned | Results not reproducible- (Questionnaire validity not mentioned, but piloting was done) | Representativeness- convenience sample  Non-responders (information and measures) not mentioned. But no concerns (83%)  Measures of risk factors and outcome- not appropriate (Questionnaire validity not mentioned) |
| (2000) Newton JT, et al. [2] | Sample size- not justified  Sample frame- convenience sampling  CI- not mentioned | ● | Representativeness- convenience sample  Non-response (measures, concerns and information)- not mentioned |
| (2000) Gray M, et al [3] | Sample size- not justified  Sample frame- convenience sampling  CI, Ethical approval and consent- not mentioned | ● | Representativeness- convenience sample  Non-response (measures, concerns and information)- not mentioned |
| (2001) Pau AKH, et al [4] | Sample size- not justified  Sample frame- convenience sampling  CI, Ethical approval- not mentioned | ● | Representativeness- convenience sample  Non-response (measures, concerns and information)- not mentioned |
| (2001) Ahmed B, et al [5] | Sample size- not justified  Sample frame- not clear  CI, Ethical approval- not mentioned | Results not reproducible (Questionnaire validity or piloting of the questionnaire) not mentioned  Limitations- not mentioned | Representativeness- sampling method not clear  Non-response (measures, concerns and information)- not mentioned  Measures of risk factors and outcome- not appropriate (Questionnaire validity and piloting not mentioned) |
| (2003) Newton JT, et al [6] | Sample size- not justified  Sample frame- convenience sampling  CI, Ethical approval and consent - not mentioned | ● | Representativeness- convenience sample  Non-response (measures and concerns)- not mentioned  But information on non-responders given |
| (2005) Dugmore CR, et al [7] | CI- not mentioned | Limitations- not mentioned | Non-responders (information, concerns and measures) not mentioned. |
| (2005) Alkhatib MN, et al [8] | Sample size- not justified  Sample frame- not clear  CI- not mentioned | Results not reproducible- sample frame not clear, questionnaire results not mentioned  Limitations- not mentioned | Representativeness- sampling not clear  Non-response (measures and information)- not mentioned  But raises no concerns on non-responders (80 %)  Results not internally consistent as no mention of questionnaire results. |
| (2007) Conway DI, et al [9] | CI, FS - not mentioned | Limitations- not mentioned | Non-response (measures and information)- not mentioned  But raises no concerns on non-responders (90 %) |
| (2008) Hullah E, et al [10] | Sample size- not justified  Sample frame- convenience sampling  CI, FS, Ethical approval and consent - not mentioned | No statistical method mentioned  Results not reproducible- (Questionnaire validity or piloting not mentioned) | Representativeness- convenience sample  Non-responders (information, concerns and measures) not mentioned |
| (2011) Reekie T. [11] | Sample size- secondary data of one cleft clinic only  Sample frame- convenience sampling | Basic results- not described in text properly  Limitations- not mentioned | Representativeness- convenience sample  Non-response (measures, concerns and information)- not applicable in secondary data |
| (2013) Marcenes W, et al [12] | CI, FS - not mentioned | ● | Non-responders (information and measures) not mentioned.  But there are concerns (response rate is 52.8 %) |
| (2013) Al-Haboubi M, et al [13] | FS- not mentioned | Results not reproducible- (Questionnaire not piloted but valid questionnaire used) | Non-response (measures and information)- not mentioned  But raises concerns on non-responders (56%) |
| (2014) Choa RM,et al [14] | Sample size- secondary data of 3 clinics  Sample frame- convenience sampling | ● | Non- response (measures, concerns and information)- not applicable.. |
| (2015) Delgado-Angulo EK, et al [15] | CI- not mentioned | ● | Response rate is 57%. So raises concerns |
| (2016) Delgado-Angulo EK, et al [16] | CI- not mentioned | ● | Response rate is 57%. So raises concerns |
| (2017) Abdelrahim R, et al [17] | CI, FS - not mentioned | ● | Non-responders (information and measures) not mentioned.  But there are concerns (response rate is 56%) |
| (2017) Arora G, et al [18] | ● | ● | Secondary data, so non-responders not applicable. No concerns on non-responders (60% response rate) |
| (2018) Weston-Price S, et al [19] | CI, FS and Ethical approval - not mentioned | Limitations- not mentioned | Non-response (measures and information)- not mentioned  But raises no concerns on non-responders (90 %) |
| (2018) Delgado-Angulo E.K, et al [20] | CI, FS - not mentioned | Results not reproducible- (Questionnaire validity or piloting of the questionnaire) not mentioned.  Basic results- Results for questionnaire neither described nor reported  Limitations- not mentioned | Non-responders (information and measures) not mentioned.  But there are concerns (response rate is 57%)  Results are not internally consistent (Questionnaire findings not reported)  Risk factors and outcomes measured correctly but reported incompletely |
| (2018) Rouxel P, et al [21] | FS, CI - Not mentioned | ○ | Non-responders (information and measures) not mentioned  But no concerns (89 %) |
| (2000) Khan FA,et al [22] | Sample frame- convenience sample  FS, CI - Not mentioned | ● | Representativeness- convenience sample  non-response (measures, concerns and information)- not mentioned |
| (2000) Vora A.R, et al [23] | Sample size- not justified  Sample frame- convenience sampling  FS, CI- not mentioned | Limitations- not mentioned  Results not reproducible- (Questionnaire validity or piloting of the questionnaire) not mentioned | Representativeness- convenience sample  Non-responders (information, concerns and measures) not mentioned. |
| (2001) Farrand P, et al [24] | Sample frame- convenience sampling  FS, CI- not mentioned  Risk factors- not clearly mentioned as questionnaire validity/ plot not mentioned | Results not reproducible- (Questionnaire validity or piloting of the questionnaire) not mentioned | Representativeness- convenience sample  Non-responders (information, concerns and measures) not mentioned |
| (2001) Pearson N, et al [25] | Sample size- not justified  Sample frame- convenience sampling  FS, CI- not mentioned | Limitations- not mentioned | Representativeness- convenience sample  Non-responders (information and measures) not mentioned  But no concerns (85%) |
| (2001) Prabhu NT, et al [26] | Sample size- not justified  Sample frame- convenience sampling  FS, CI- not mentioned | Results not reproducible- (Questionnaire validity or piloting of the questionnaire) not mentioned | Representativeness- convenience sample  Non-responders (information and measures) not mentioned  But no concerns (70.3%) |
| (2013) Csikar J,et al [27] | FS, CI- not mentioned | Limitations- not mentioned | Non-response (measures, concerns and information)- not applicable in secondary data |
| (2013) Siddique I, et al [28] | Sample size- not justified  Sample frame- convenience sampling  FS, CI- not mentioned | Results not reproducible- (Questionnaire validity or piloting of the questionnaire) not mentioned | Representativeness- convenience sample  Non-responders (information, concerns and measures) not mentioned |
| (2016) Merchant R, et al [29] | Sample size- not justified  Sample frame- convenience sampling  FS, CI- not mentioned | Results not reproducible- (Questionnaire validity or piloting of the questionnaire) not mentioned | Representativeness- convenience sample  Non-responders (information, concerns and measures) not mentioned |
| (2002) Ugur ZA, et al [30] | Sample size calculations - not justified  Sample frame- convenience sample  Ethical approval, FS, CI - Not mentioned | Results not reproducible- (Clinical examination was done using correct index, but questionnaire validity or piloting of the questionnaire) not mentioned | Representativeness- convenience sample  Non-response (measures and information)- not mentioned  But no concerns (96.7%) |
| (2003) Kühnisch, J, et al [31] | Sample size calculations - not justified  Sample frame- not clear  Ethical approval, consent, FS, CI - Not mentioned | Limitations- Not mentioned | Representativeness- sampling method not clear  Non-response (measures, concerns and information)- not mentioned |
| (2004) Van Steenkiste M, et al [32] | Ethical approval, consent, FS, CI - Not mentioned | ● | Non-response (measures, concerns and information)- not mentioned |
| (2004) Van Steenkiste M,et al [33] | Ethical approval, consent, FS, CI - Not mentioned | ● | Non-response (measures, concerns and information)- not mentioned |
| (2007) Schenk, L, et al [34] | CI- not mentioned | Limitations- Not mentioned | ● |
| (2007) Heinrich-Weltzien R, et al [35] | Sample size calculations - not justified  Ethical approval, consent, CI - Not mentioned | Limitations- Not mentioned | Representativeness- sampling method not clear  non-response (measures, concerns and information)- not mentioned |
| (2007) Bissar AR, et al [36] | Sample size calculations - not justified  Sample frame- convenience sample  CI - Not mentioned | Limitations- Not mentioned | Representativeness- convenience sample  Non-response (measures, concerns and information)- not mentioned |
| (2017) Erdsiek F, et al [37] | Sample size calculations - not justified  Ethical approval - Not mentioned | ● | Non-response (measures, concerns and information)- not mentioned |
| (2017) Brzoska P, et al [38] | Sample frame- not representative of migrants | ● | Representativeness- not representative  Non-response (measures, concerns and information)- not mentioned |
| (2018) Aarabi G, et al [39] | Sample size calculations- not justified  Sample frame- convenience sample  Ethical approval - Not mentioned | ● | Representativeness- convenience sample  Non-response (measures and information)- not mentioned  But no concerns (83%) |
| (2000) Hjern A, et al [40] | CI not mentioned | Limitations- Not mentioned  Results not reproducible- (Questionnaire validity or piloting of the questionnaire) not mentioned  Basic results not described clearly in table. | Non-response (measures- not mentioned)  But no concerns (more than 60% response rate in all groups) and information on non-responders given. |
| (2001) Hjern A, et al [41] | FS, CI, Ethical approval and consent- Not mentioned | Limitations- Not mentioned  Results not reproducible- (Questionnaire validity or piloting of the questionnaire) not mentioned  Basic results not described clearly in table. | Non-response (measures, concerns and information on non-responders- not mentioned) |
| (2004) Stecksén-Blicks C, et al [42] | CI and Ethical approval - Not mentioned | Limitations- Not mentioned  Results not reproducible- (Clinical examination was done using correct index, but questionnaire validity or piloting of the questionnaire) not mentioned | Non-response (measures, concerns and information)- not mentioned. |
| (2005) Jacobsson B , et al [43] | Sample frame- convenience sample  CI and consent- Not mentioned | Limitations- Not mentioned  Results not reproducible- (Clinical examination was done using correct index, but questionnaire validity or piloting of the questionnaire) not mentioned | Representativeness- convenience sample  non-response (measures, concerns and information)- not mentioned |
| (2008) Stecksén-Blicks C,et al [44] | CI and Ethical approval - Not mentioned | Limitations- Not mentioned  Results not reproducible- (Clinical examination was done using correct index, but questionnaire validity or piloting of the questionnaire) not mentioned | Non-response (measures, concerns and information)- not mentioned. |
| (2010) Julihn A, et al [45] | CI and Ethical approval - Not mentioned | Results not reproducible- (Clinical examination was done using correct index, but questionnaire validity or piloting of the questionnaire) not mentioned | Non-response (measures- not mentioned)  But no concerns (more than 50% response rate) and information on non-responders given |
| (2010) Mousavi SM, et al [46] | FS, CI, Ethical approval - Not mentioned | Limitations- Not mentioned | Non-response (measures, concerns and information)- not applicable in secondary data |
| (2014) Stecksén-Blicks C, et al [47] | Ethical approval - Not mentioned | Limitations- Not mentioned  Results not reproducible- (Clinical examination was done using correct index, but questionnaire validity or piloting of the questionnaire) not mentioned | Non-response (measures, concerns and information)- not mentioned. |
| (2016) Olerud E, et al [48] | Sample size calculation- not justified  Sample frame- convenience sample  CI- not mentioned | ● | Representativeness- convenience sample.  Non-response (measures, concerns and information) not mentioned. |
| (2004) Ferro R,et al [49] | Sample size calculation- not justified  Sample frame- convenience sample  FS, CI, Ethical approval and consent- Not mentioned | Methods not reproducible  Results neither discussed nor reported properly | Representativeness- convenience sample.  Non-response (measures, concerns and information) not mentioned.  Risk and outcomes measures- not clearly reported |
| (2007) Ferro R, et al [50] | Sample size calculations- not justified  FS and CI not mentioned | Limitations- Not mentioned | Non-response (measures and information)- not mentioned.  But no concerns (73%) |
| (2007) Ferro R,et al [51] | Sample size calculations- not justified  FS and CI not mentioned | Limitations- Not mentioned | Non-response (measures and information)- not mentioned.  But no concerns (81%) |
| (2007) Ferro R, et al [52] | Sample size calculations- not justified  FS, CI and Ethical approval not mentioned | Limitations- Not mentioned | non-response (measures and information)- not mentioned.  But no concerns (More than 80% response rate) |
| (2018) Petti S, et al [53] | Sample size calculations- not justified  Sample frame- convenience sample  Ethical approval, FS, Consent - Not mentioned | Limitations- Not mentioned | Representativeness- convenience sample  non-response (measures and information)- not mentioned.  But no concerns (72%) |
| (2005) Skeie MS, et al [54] | CI not mentioned | Limitations- Not mentioned | non-response (measures and information)- not mentioned.  But no concerns (86.1%) |
| (2006) Skeie MS,et al [55] | CI not mentioned  Sample frame- convenience | Limitations- Not mentioned | Representativeness- convenience sample.  Non-response (measures)- not mentioned.  Information on non-responders given.  No concerns (more than 60% response rate in both the groups) |
| (2008) Skeie MS, et al [56] | CI not mentioned  Sample frame- convenience | Limitations- Not mentioned | Representativeness- convenience sample.  Non-response (measures)- not mentioned.  Information on non-responders given.  No concerns (more than 75% response rate in both the groups) |
| (2010) Skeie MS,et al [57] | CI not mentioned  Sample frame- convenience | Limitations- Not mentioned | Representativeness- convenience sample.  Non-response (measures)- not mentioned.  Information on non-responders given.  No concerns (more than 75% response rate in both the groups) |
| (2010) Wigen TI, et al [58] | FS and CI not mentioned | Limitations- Not mentioned | Risk factors measurement – not correct as questionnaire validity/ pilot not mentioned  Non-response (measures, concerns and information)- not mentioned. |
| (2006) Almerich Silla JM, et al [59] | FS, CI, Ethical approval and consent- Not mentioned | Basic results- not correct as pdl of migrants not compared to host.  Limitations- Not mentioned | Non-response (measures, concerns and information)- not mentioned. |
| (2007) Almerich-Silla JM, et al [60] | FS, CI and Ethical approval- Not mentioned | Limitations- Not mentioned | Non-response (measures, concerns and information)- not mentioned. |
| (2018) Muñoz‑Pino N, et al [61] | ● | Results not reproducible- (Questionnaire validity or piloting of the questionnaire) not mentioned | Non-response (measures and information)- not mentioned.  No concerns (71.06%) |
| (2019) Valcarcel Soria R, et al [62] | Sample frame- convenience  FS- Not mentioned | Results not reproducible- (Questionnaire validity or piloting of the questionnaire) not mentioned | Representativeness- convenience sample.  Non-response (measures and information) not mentioned, but no concerns on non-response (94%)  Risk factors measurement – not correct as questionnaire validity/ pilot not mentioned |
| (2015) Duijster D, et al [63] | Sample frame- convenience  CI not mentioned | ● | Representativeness of sample- convenience sampling.  Non-response (measures not mentioned, concerns are raised 34%, but information given) |
| (2016) van der Tas JT, et al [64] | ● | ● | Non-response (measures, concerns and information)- not mentioned. |
| (2003) Sundby A, et al [65] | Sample size- not justified  Sample frame- convenience sampling  FS, CI, Ethical approval and consent- Not mentioned | Limitations- Not mentioned | Representativeness of sample- convenience sampling.  Risk factors and outcomes measures- not correct as questionnaire validity/ pilot not mentioned.  Non-response (measures and information)- not mentioned.  But no concerns (63.5%) |
| (2010) Christensen LB, et al [66] | FS and CI- Not mentioned | Limitations- Not mentioned | Non-response (measures, concerns and information)- not applicable in secondary data |
| (2011) Gatou T, et al [67] | FS and CI- Not mentioned  Consent- Not mentioned | Limitations- Not mentioned | Non-response (measure and information)- not mentioned.  No concerns (65.8%) |
| (2017) Mantonanaki. M, et al [68] | FS and CI- Not mentioned | Limitations- Not mentioned | Non-response (measures and information)- not mentioned.  But raises concerns (58%) |
| (2014) Cvikl B, et al [69] | FS and CI- Not mentioned | ● | Non-response (measures, concerns and information)- not mentioned. |
| **TOTAL (%)** | **3/69= 4.34%** | **20/69= 28.9%** | **1/69= 1.4%** |

Criteria satisfied ● Criteria not satisfied ○
